# Alfalfa Cellulose Synthase Gene Expression under Abiotic Stress: A Hitchhiker’s Guide to RT-qPCR Normalization

**DOI:** 10.1371/journal.pone.0103808

**Published:** 2014-08-01

**Authors:** Gea Guerriero, Sylvain Legay, Jean-Francois Hausman

**Affiliations:** Department Environment and Agro-biotechnologies (EVA), Centre de Recherche Public, Gabriel Lippmann, Belvaux, Luxembourg; Iowa State University, United States of America

## Abstract

Abiotic stress represents a serious threat affecting both plant fitness and productivity. One of the promptest responses that plants trigger following abiotic stress is the differential expression of key genes, which enable to face the adverse conditions. It is accepted and shown that the cell wall senses and broadcasts the stress signal to the interior of the cell, by triggering a cascade of reactions leading to resistance. Therefore the study of wall-related genes is particularly relevant to understand the metabolic remodeling triggered by plants in response to exogenous stresses. Despite the agricultural and economical relevance of alfalfa (*Medicago sativa* L.), no study, to our knowledge, has addressed specifically the wall-related gene expression changes in response to exogenous stresses in this important crop, by monitoring the dynamics of wall biosynthetic gene expression. We here identify and analyze the expression profiles of nine cellulose synthases, together with other wall-related genes, in stems of alfalfa plants subjected to different abiotic stresses (cold, heat, salt stress) at various time points (e.g. 0, 24, 72 and 96 h). We identify 2 main responses for specific groups of genes, i.e. a salt/heat-induced and a cold/heat-repressed group of genes. Prior to this analysis we identified appropriate reference genes for expression analyses in alfalfa, by evaluating the stability of 10 candidates across different tissues (namely leaves, stems, roots), under the different abiotic stresses and time points chosen. The results obtained confirm an active role played by the cell wall in response to exogenous stimuli and constitute a step forward in delineating the complex pathways regulating the response of plants to abiotic stresses.

## Introduction

The study of biological phenomena requires several sensitive analytical techniques, which can convey detailed information at different depths of organismal complexity, namely tissular, metabolic, genomic. One such type of information is represented by gene expression changes, which provide clues about transcripts dynamics, e.g. in response to exogenous stimuli.

Currently one of the most reliable and reproducible methods to perform differential gene expression profiling is quantitative reverse transcription PCR (hereafter referred to as RT-qPCR), a method which is robust enough to quantify challenging targets, as microRNAs (miRNAs) e.g. [1]. However, accurate gene expression analyses rely on several critical aspects and experimental steps (namely RNA purity and integrity, genomic DNA contamination, reverse transcription) and, in the case of relative quantification, on the identification of suitable reference genes for data normalization [2]–[3]. Those are genes whose expression is stable and not subject to fluctuations across the different conditions tested. This feature is particularly critical, as the choice of inappropriate reference genes can significantly bias the results obtained and therefore lead to misinterpretations of biological events.

The use of RT-qPCR is particularly suitable to study the response of a set of genes in plants after the application of specific stresses e.g. [4]: being sessile organisms, plants are not capable of escaping from adverse environmental conditions and are therefore characterized by a very responsive transcriptional regulation, which results in phenotypic plasticity [5]–[7]. Abiotic stresses constitute serious threats for plants, as they can affect not only their development, growth, reproduction and productivity, but can be so detrimental to cause their death. Exogenous stresses unleash a cascade of reactions, which lead to plant response and resistance, usually by means of wall fortification.

Many studies in the literature have provided a comprehensive view of gene expression changes in different plant species in response to abiotic stresses and identified a list of suitable reference genes for data normalization e.g. [8]–[13]. These studies have also shown how the expression of reference genes can vary in different plant species and conditions and how important it is to validate their stability in the specific experimental set-ups used.

Despite the agricultural and economical importance of the legume crop *Medicago sativa* L. (a.k.a alfalfa, or lucerne), no study has so far tested suitable reference genes for expression analysis using RT-qPCR in this plant. Suitable reference genes have been identified in *Medicago truncatula* [14] and potential reference genes in alfalfa have been proposed by Yang et al. [15], however their suitability for RT-qPCR studies has, to our knowledge, never been validated so far.

Alfalfa is an experimentally valuable model: it is not only suitable for the study of symbiotic interactions e.g. [16], but has also been proposed as an excellent model system to study dicot cell wall development [17]. Its stem shows indeed 2 clearly-defined regions characterized by active elongation and lignification/thickening, which provide “snapshots” of the cell wall maturation process. Although the genome of alfalfa has not yet been sequenced, several studies have shown the suitability of using the genome of the closely related barrel medic (*M. truncatula*) [18] for molecular analyses. These studies have delivered valuable information concerning the regulation of wall polysaccharide biosynthesis in cultivars with contrasting cell wall composition [19].

The aim of the present study is to provide a time-course analysis of cell wall-related gene expression in response to different abiotic stresses in alfalfa stems. In particular we analyzed nine cellulose synthases (hereafter named *MsCesA*s), identified on the basis of the sequence homology with the orthologs from *M. truncatula* (*MtCesA*s), together with other genes linked to wall biogenesis (namely sucrose synthase, *SuSy*; phenylalanine ammonia lyase, *PAL*; cinnamyl alcohol dehydrogenase, *CAD*; cellulose synthase-like gene, *CslD4*). To perform a reliable gene expression analysis, accurate data normalization is mandatory, which prompted us to identify the most suitable reference genes for expression analysis. We chose as candidate reference genes a set of known and widely used genes which have been tested on *M. truncatula* [14], together with candidates proposed by Yang et al. [15] and Huis et al. [20].

We decided to extend our survey not only to stems, but also to other tissues (namely roots and leaves), in order to provide a list of genes to be used in tissue- and/or growth condition-specific studies in alfalfa and which can be tested in other legume crops too. Ten candidate reference genes were chosen and their reliability for RT-qPCR studies tested at different time-points in different tissues of *M. sativa* plants exposed to abiotic stresses. To further validate their suitability, we studied the expression of a stress-associated kinase (*SK1*) in the different tissues and growth conditions.

Despite the unanimously recognized role of plant walls as cellular structures sensing and responding to stimuli [21]–[24] and despite the economic significance of alfalfa, to our knowledge no study is yet available on the expression analysis of key wall biosynthetic genes (as *CesA*s) in response to different abiotic stresses in *M. sativa*. We here provide such a study and identify the main trends characterizing the response to abiotic stresses in alfalfa stems.

## Materials and Methods

### Plant growth and abiotic stress treatments

*Medicago sativa* L. seeds, variety Giulia (Italy), were inoculated with a peat-based inoculant (HiStick, Becker Underwood) according to the manufacturer’s instructions. Five seeds were sown per pot in 1 L containers filled with soil (50% topsoil, 25% potting soil, 25% sand). After 4 weeks of cultivation under controlled greenhouse conditions (photoperiod of 13 h light/11 h darkness, minimum temperature of 20°C, maximum 27°C), plants were moved to incubators (programmed to provide exactly the same light/dark cycles) for moderate cold and heat stress treatments, while those subjected to moderate salt stress were left in the greenhouse. For the cold stress condition, plants were grown at a constant temperature of 5°C; for the heat stress condition, they were grown at 28°C/32°C (night/day); for the salt stress treatment, plants were supplemented with 100 mM NaCl. A total of 3 biological replicates (each consisting of a pool of 15 plants) were used per treatment. For each time point studied (0- 24- 72- 96 h) a control group was always kept (i.e. plants grown without any treatment for 24- 72- 96 h), for appropriate comparisons.

### RNA extraction and cDNA synthesis

Sampled tissues (roots, leaves and whole stems) were ground to a fine powder in liquid nitrogen, using a mortar and a pestle. One hundred mg of finely ground sample were weighed on a balance and total RNA was extracted using the RNeasy Plant Mini Kit with the on-column DNase I treatment (Qiagen). The integrity of the extracted RNA was checked with an Agilent Bioanalyzer (all the RINs were >8) and the purity/concentration measured using a NanoDrop ND-1000 spectrophotometer (A260/280 and A260/230 ratios between 1.9 and 2.2). Subsequently, 1 µg of extracted RNA was retro-transcribed using the Superscript II cDNA Synthesis kit (Invitrogen), according to the manufacturer’s instructions.

### Identification of CesA genes from alfalfa

To identify and amplify putative *CesA* genes from *M. sativa*, initial data mining was performed on *M. truncatula* [25], a closely related species for which the genome is available. A total of nine putative *M. truncatula* cellulose synthase proteins (hereafter indicated MtCESAs) were identified. BLASTp searches were performed against non-redundant protein databases of *Arabidopsis thaliana* and *Populus trichocarpa* from the National Centre for Biotechnology [26] to check the percentage of identity of the identified sequences. To amplify the orthologous genes from alfalfa, primers were designed on the identified *MtCesA*s genes (listed in Table S1). Three full-length *CesA* genes from alfalfa were identified and designated *MsCesA3*, *MsCesA4* and *MsCesA7-A* [GenBank: KJ398155, KJ398156, KJ398157; Fig. S1], on the basis of their phylogenetic kinship, while partial sequences were obtained for the other *MsCesA*s (Figs. S1 and S2). The phylogenetic tree was built by aligning the amino acid regions of CESAs from *M. truncatula*, *M. sativa*, *P. trichocarpa* and *A. thaliana* encompassing the U1–U4 regions, the QXXRW motif and the HVR2 region, which allows class discrimination [27], using MUSCLE [28]. Phylogeny was analyzed using PhyML [29]. The maximum-likelihood phylogenetic tree was rendered using TreeDyn [30]. Microarray data for *M. truncatula CesA*s were retrieved at [31] and electronic fluorescent pictographic (eFP) representations at [32].

### Quantitative real-time PCR and statistical analysis

For quantitative real-time PCR analysis, 10 ng cDNA were used as template. The cDNA was amplified using the MESA GREEN qPCR MasterMix Plus.

Low ROX (Eurogentec) on a ViiA 7 Real-Time PCR System (Applied Biosystems) in a final volume of 25 µl.

The reactions were performed in technical triplicates and repeated on the above-described 3 biological independent replicates. The PCR conditions consisted of an initial denaturation at 95° for 10 min, followed by 45 cycles of denaturation at 95° for 15 sec, annealing/extension at 60° for 60 sec.

A dissociation kinetics analysis was performed at the end of the experiment to check the specificity of the annealing.

Ten candidate reference genes were analyzed, namely actin, tubulin, ubiquitin-conjugating protein 13 (UBC13), cyclophilin (cyclo), elongation initiation factor 4A (eif4A), elongation initiation factor 5A (eif5A), translation initiation factor IIA (TFIIA), glyceraldehyde-3P dehydrogenase (GAPDH), actin-depolymerizing protein (ADF1), poly(A) binding protein 4 (PAB4). Their stability was evaluated using NormFinder [33] and geNorm^PLUS^ [2], two of the most commonly used software, which rank candidate reference genes on the basis of their stability. The software geNorm^PLUS^ performs a pairwise comparison and computes the M-value, i.e. the variation of a gene compared to all the remaining candidates, while NormFinder computes first the intra-group and subsequently the inter-group expression variability of a candidate reference gene [33]–. NormFinder calculates both a single best gene (best gene) and an optimal gene pair (best pair); the best pair might display compensating expression in the different experimental groups. The candidate reference genes primers for actin, tubulin, GAPDH were designed using the sequences from *M. truncatula* [GenBank: XM_003621971, XM_003603622, XM_003595990]. The primers for the other reference genes were designed using the sequences of the candidate housekeeping genes reported by Yang et al. [15], which show an average RPKM-normalized value higher than 10 and the lowest coefficient of variation identified with RNAseq. The list of primers used to perform RT-qPCR analyses is shown in Table S2. The RT-qPCR primers for *CAD* and *CslD4* (Table S2) were designed on the sequences from *M. truncatula* genes (probesets Mtr.8985.1.S1_at and Mtr.45005.1.S1_at, respectively) [19], while those for *SuSy* and *PAL* (Table S2) were designed on the reported sequences from alfalfa (probeset Msa.2902.1.S1_at) [19] and [GenBank: CAA41169]. Primers were designed using Primer3Plus [35] and analyzed with OligoAnalyzer 3.1 [36]. The primers size was 20 bp, the amplicon sizes were between 70–150 bp (Table S2), the %GC was between 40–60% and Tm 60°C. The primers used were not intron spanning. Primer efficiencies were tested and are reported in Table S2. All the amplicons were verified by sequencing on an Applied Biosystems 3500 Genetic Analyser using the BigDye Terminator v3.1 Cycle Sequencing and the BigDye XTerminator Purification kits, according to the manufacturer’s instructions.

The results relative to the expression of the target genes were analyzed using the software qBase^PLUS^ version 2.5 (Biogazelle, [37]) and normalized taking into account the most stable reference genes (as indicated in the text). The expression levels of the genes detected in the different tissues and conditions analyzed are here expressed as “Normalized relative expression”. A one-way ANOVA (with Tukey’s HSD post-hoc test) was performed on the log_2_ transformed calibrated normalized relative quantities (CNRQs), using IBM SPSS Statistics (version 19), after having checked the normal distribution of the data with a Kolmogorov-Smirnov test.

Hierarchical clustering was generated with Cluster 3.0 [38] and visualized with Java TreeView [39], available at [40].

## Results

### Stability of putative reference genes in different tissues of *M. sativa* subjected to abiotic stresses

**Figure 1:**
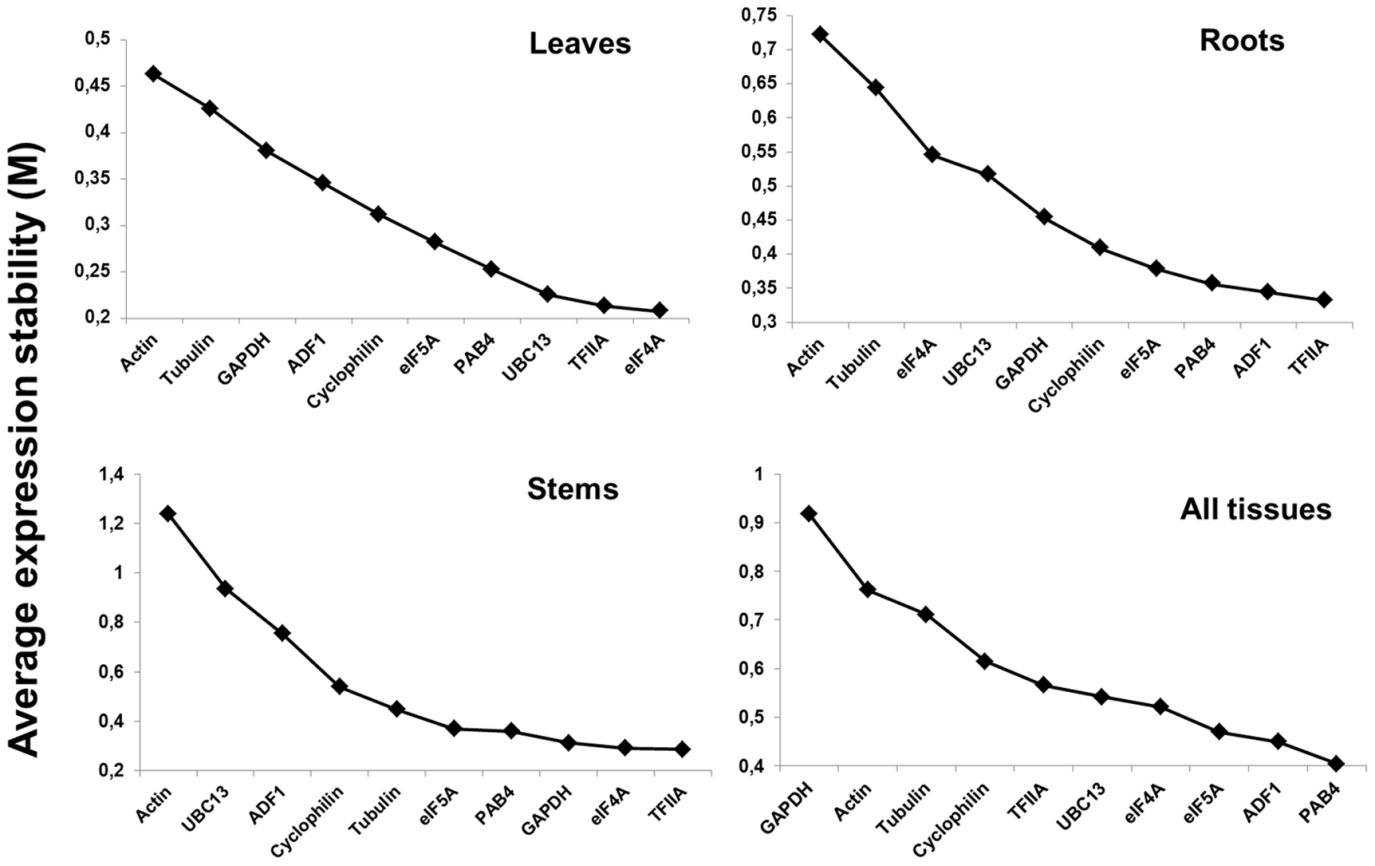
Candidate reference genes in alfalfa. Ranking of ten candidate reference genes in different tissues of *M. sativa* according to the parameter M calculated by geNorm^PLUS^. Increasing stability of the candidate genes is determined by a decrease in the M value.

Analyses with geNorm^PLUS^ were performed to rank the expression stability of the 10 candidate reference genes in the tissues and conditions analyzed (Fig. 1). According to geNorm^PLUS^, TFIIA ranks among the most stable genes in roots, leaves and stems, but interestingly this gene is not among the most stably expressed when all the tissues are grouped together (Fig. 1). However NormFinder ranks TFIIA among the 4 most stable genes when all the tissues are taken into account (Table 1). The gene eIF4A is very stable in leaves and stems, while it is among the least stable in roots (Fig. 1). The most stably expressed genes when all the tissues are grouped together are ADF1 and PAB4 (Fig. 1). Actin is among the least stable genes in all the conditions tested (Fig. 1). These results show that care should be taken when choosing candidate reference genes for expression analysis in different plant tissues, as stable genes in a specific tissue might not be suitable for normalization of expression data in another one. In the literature, several studies have shown the importance of determining the stability of the reference genes in the different plant tissues, in order to use the most reliable ones in the condition examined e.g. [11], [20]. The stability data obtained with geNorm^PLUS^ have been compared to the rankings generated via the other widely used software, i.e. NormFinder. Ranking lists were generated for each single tissue under all the conditions tested, for all the tissues together under each single condition (which can be of particular interest when a specific abiotic stress is studied), as well as for all the tissues and conditions together (Table 1). From the rankings it is possible to confirm the expression stability of TFIIA in the single tissues under the different stress treatments; in particular the pairs ADF1/TFIIA and eIF4a/TFIIA are confirmed as the most suitable genes for normalization in roots and stems respectively (Table 1). PAB4 ranks always among the 5 most stable genes when all the tissues are analyzed together in each of the conditions tested, while the high stability of eIF5A and PAB4 is confirmed when all the tissues and conditions are taken into consideration (Table 1). Both geNorm^PLUS^ and NormFinder show how unsuitable tubulin and actin are for data normalization in our experimental system: this is quite important, as these genes, although suitable for normalization in some instances [41]–[42], might not be ideal in others [8], [20], [43]. The best gene pairs identified by NormFinder are different in some tissues from those identified by geNorm^PLUS^, a finding which has already been reported in other studies e.g. [20] and might be due to the different ranking methodology used by the two softwares: normalization in the leaves requires TFIIA and eIF4A according to geNorm^PLUS^, while NormFinder suggests ADF1 and PAB4, which in the geNorm^PLUS^ ranking are the 4^th^ and 7^th^ least stable gene, respectively (Fig. 1). Similarly, if all the tissues and stresses are considered, the best gene pair is GAPDH/PAB4 according to NormFinder, however geNorm^PLUS^ ranks GAPDH as the most unstable gene in this configuration (Fig. 1).

**Table 1:** Ranking of candidate reference genes according to NormFinder.

	Leaves (all treatments)		Roots(all treatments)		Stems(all treatments)		Control(all tissues)		Cold(all tissues)		Heat(all tissues)		Salt(all tissues)		All tissues and treatments	
	Gene	Stability	Gene	Stability	Gene	Stability	Gene	Stability	Gene	Stability	Gene	Stability	Gene	Stability	Gene	Stability
	Act	0.150	Act	0.251	Act	0.306	Act	0.117	Tub	0.148	Tub	0.280	ADF1	0.154	Tub	0.168
	Tub	0.126	Tub	0.211	ADF1	0.153	TFIIA	0.109	eIF5A	0.125	Act	0.202	Act	0.105	Act	0.133
	eIF5A	0.103	Cyclo	0.139	Tub	0.143	Tub	0.108	Act	0.106	eIF5A	0.118	Cyclo	0.080	UBC13	0.084
	GAPDH	0.091	UBC13	0.138	Cyclo	0.128	eIF5A	0.104	TFIIA	0.093	Cyclo	0.116	Tub	0.068	eIF4A	0.076
	UBC13	0.087	eIF5A	0.110	UBC13	0.127	GAPDH	0.059	GAPDH	0.081	UBC13	0.112	eIF5A	0.073	Cyclo	0.076
	Cyclo	0.086	PAB4	0.102	eIF5A	0.102	Cyclo	0.053	ADF1	0.064	GAPDH	0.109	TFIIA	0.050	ADF1	0.074
	ADF1	0.072	eIF4A	0.094	PAB4	0.081	eIF4A	0.053	eIF4A	0.054	PAB4	0.085	GAPDH	0.049	TFIIA	0.067
	eIF4A	0.056	GAPDH	0.089	eIF4A	0.067	UBC13	0.051	UBC13	0.051	eIF4A	0.080	PAB4	0.046	eIF5A	0.058
	TFIIA	0.051	TFIIA	0.071	GAPDH	0.062	ADF1	0.040	PAB4	0.047	ADF1	0.074	UBC13	0.041	GAPDH	0.055
**Best gene**	PAB4	0.043	ADF1	0.036	TFIIA	0.046	PAB4	0.033	Cyclo	0.041	TFIIA	0.040	eIF4A	0.029	PAB4	0.050
**Best pair**	ADF1/PAB4	0.033	ADF1/TFIIA	0.040	TFIIA/eIF4A	0.040	Cyclo/eIF4A	0.019	Cyclo/PAB4	0.033	Cyclo/GAPDH	0.042	GAPDH/UBC13	0.020	GAPDH/PAB4	0.010

The best gene and the best combination of genes are shown. The analysis has been carried out to find the most stable reference genes in the different tissues under all the treatments tested, in all the tissues under different treatments and when all the tissues and treatments studied are grouped together. Abbreviations here used: Act (actin), Tub (tubulin), GAPDH, Cyclo (cyclophilin).

Nevertheless, taking into account the rankings of NormFinder and geNorm^PLUS^, it emerges that for expression studies in alfalfa tissues (and possibly in other legume crops) actin and tubulin are not ideal, whereas a suitable panel of reference genes should include eIF4A, PAB4, ADF1 and TFIIA, as they rank among the most stable genes according to the two softwares. This result can be of particular interest when studying gene expression in different plant tissues subjected to a specific treatment: if a tissue-maximization strategy is selected in the experimental design, it is helpful to know *a priori* which panel of candidates to include for stability test.

### Optimal number of reference genes for normalization in *M. sativa* tissues using geNorm^PLUS^ ranking

**Figure 2:**
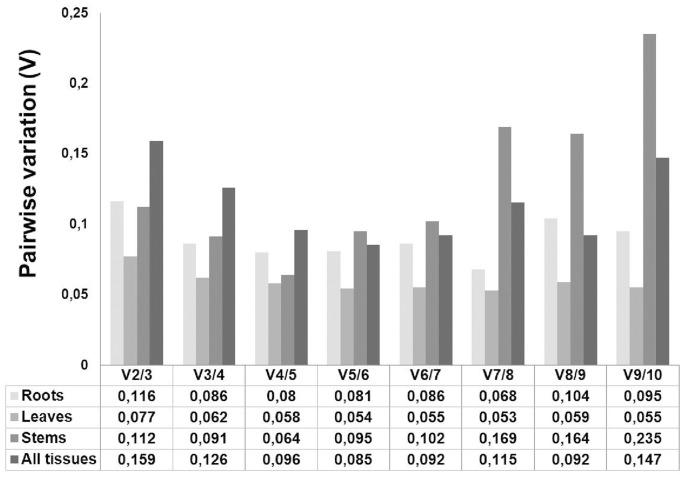
Determination of the appropriate number of reference genes for data normalization in *M. sativa* tissues under abiotic stress conditions, as computed by geNorm^PLUS^. The pairwise variation (Vn/Vn+1) was calculated between the normalization factors NFn and NFn+1. The recommended cut-off threshold of 0.15 was kept in the present study.

In order to calculate the appropriate number of reference genes for data normalization in alfalfa, we used geNorm^PLUS^ to compute the pairwise variation (Vn/Vn+1) between two consecutive normalization factors (NFn and NFn+1). The analysis shows that for accurate normalization in roots, stems and leaves, 2 reference genes are required: the addition of a third gene is indeed not necessary, as the V value relative to 2 reference genes is already below the cut-off threshold of 0.15 (Fig. 2). However, if all the tissues are grouped together, the number of genes required for accurate data normalization increases to 3, since the V value relative to 2 genes is above the cut-off threshold (0.159) (Fig. 2).

### Validation of the selected reference genes in different tissues

The validity of the candidate reference genes identified via the geNorm^PLUS^ and NormFinder analyses was tested in the different tissues and conditions by studying the expression profiles of a stress-associated kinase orthologous to *MtSK1* [GenBank: XP_003592980] [44]. This gene is a member of the SnRK group of plant kinases and was shown to be induced upon wounding in cultured tissues [44].

Since SnRKs are involved in stress response in plants e.g. [45], we decided to use this gene both to validate the identified reference genes in the different conditions and to study its expression profile in response to different abiotic stresses in alfalfa tissues. It was assumed that the experimental treatment would not alter the expression of the reference genes, but would instead affect the expression of the stress-associated kinase. The data were analyzed with qBASE^PLUS^ and normalized using ADF1/.

**Figure 3:**
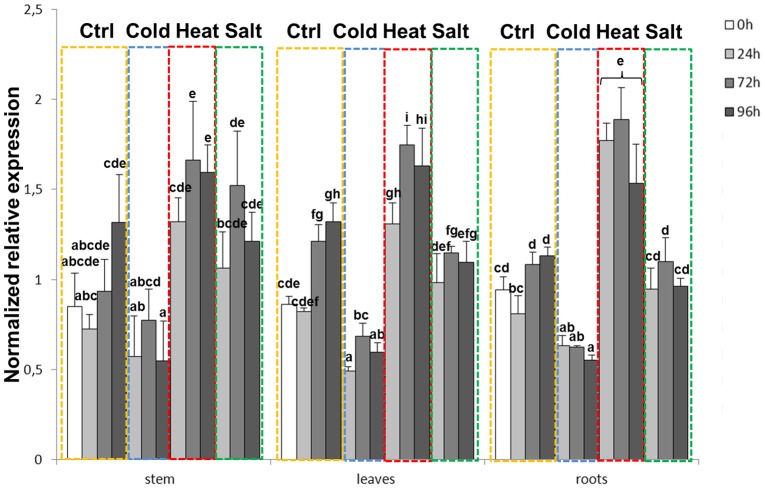
*MsSK1* expression in alfalfa tissues under abiotic stresses. Expression profiles of *MsSK1* in the different tissues under abiotic stresses (yellow dotted frame is control; blue dotted frame is cold stress; red dotted frame is heat stress; green dotted frame is salt stress). The Y-axis indicates NRE (Normalized Relative Expression of *MtSK1*). Data were normalized using ADF1/TFIIA and eIF4A/TFIIA for the roots and stems respectively and TFIIA/eIF4A for the leaves. Means sharing a letter are not significantly different at α = 0.05.

**Figure 4:**
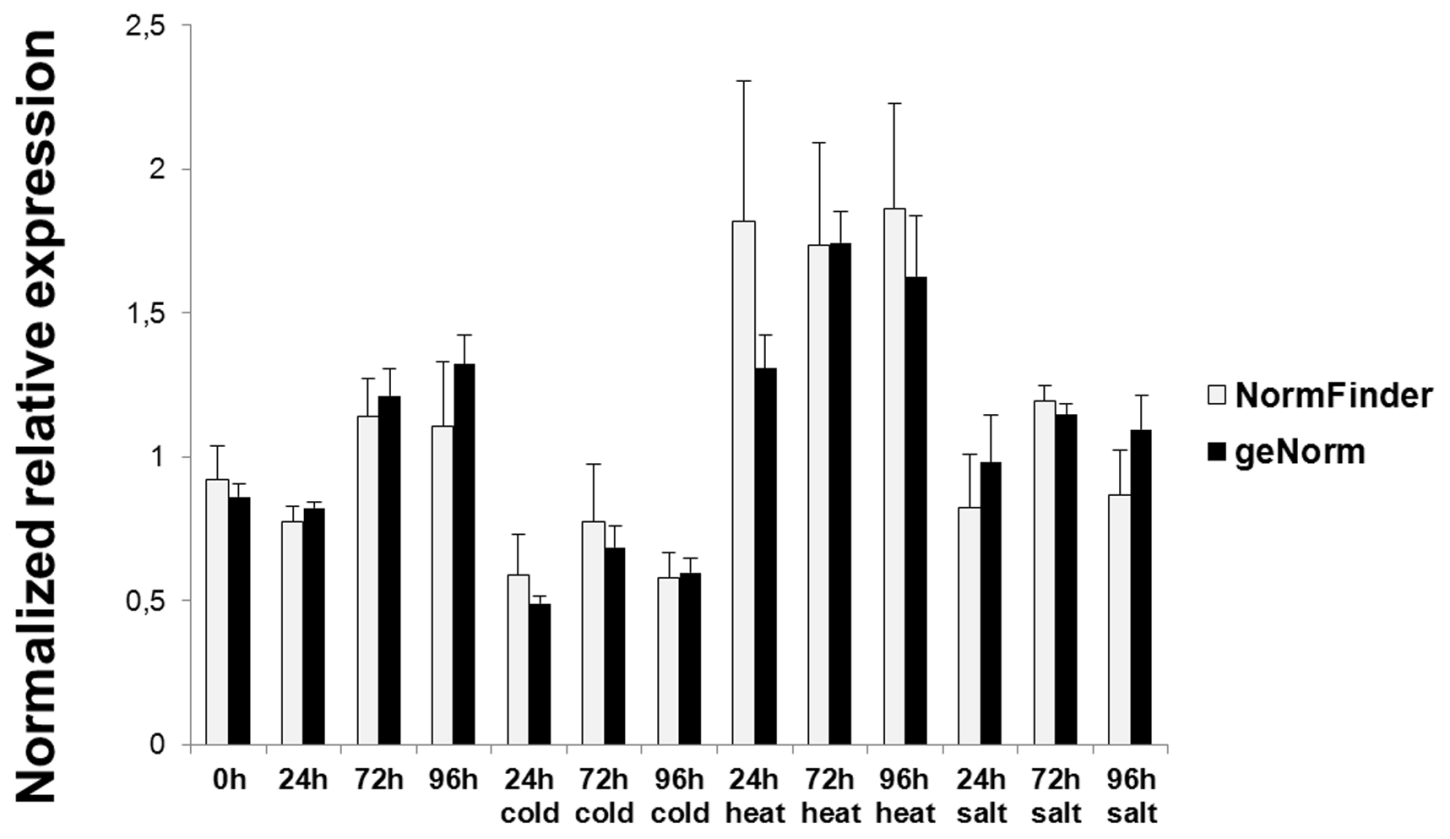
Comparison of NormFinder and geNorm^PLUS^ normalization methods. Comparison of *MsSK1* expression profiles in leaves when normalization is performed using ADF1/PAB4 (according to NormFinder), or TFIIA/eIF4A (according to geNorm^PLUS^).

TFIIA and eIF4A/TFIIA for the roots and stems respectively, since these candidates were selected by both geNorm^PLUS^ and NormFinder, then a comparison of normalization strategies was performed for the leaves (Figs. 3 and 4), since the two softwares chose different candidates (namely ADF1/PAB4 by NormFinder and TFIIA/eIF4A by geNorm^PLUS^; Fig. 1 and Table 1). As can be seen in Fig. 3, the stresses which triggered the most significant changes were cold and heat: in all the tissues examined, a significant decrease in expression could indeed be observed during cold stress treatment, while heat stress induced expression, where the highest increase was present in roots. Salt stress, on the other hand, did not appreciably change the expression of the stress-associated kinase, apart from a mild increase at 24 and 72h in the stems (Fig. 3). This result was unexpected, as it was previously shown that the expression of the ortholog from *M. truncatula* increased in the leaves after salt stress treatment [44], however it should be noted that the analysis was here performed on another species and that fluctuations in expression were observed in control condition over the different time-points (Fig. 3). These fluctuations contribute to make the expression changes not significant.

In order to compare the normalization strategies using the gene pairs recommended by NormFinder and geNorm^PLUS^, we chose to perform a test on the leaves, since for the roots and the stems the two softwares agreed on the best gene pairs (Fig. 1 and Table 1). As can be seen in Fig. 4, the expression trend in response to the different stresses did not change: different normalized relative expression values could be observed for a same time point between the NormFinder and geNorm^PLUS^ normalization (Fig. 4). In particular, higher error bars could be observed at some time points (e.g. 24 h heat, 72 h heat, 96 h heat) for the expression values obtained with NormFinder-based normalization (Fig. 4): this is most likely a reflection of the intrinsic computing differences of the two algorithms. However the Student’s t-test did not show statistically significant differences between the magnitude changes calculated by NormFinder and geNorm^PLUS^ (not shown).

### Identification and phylogenetic analysis of CesAs from *M. truncatula* and *M. sativa*

**Figure 5:**
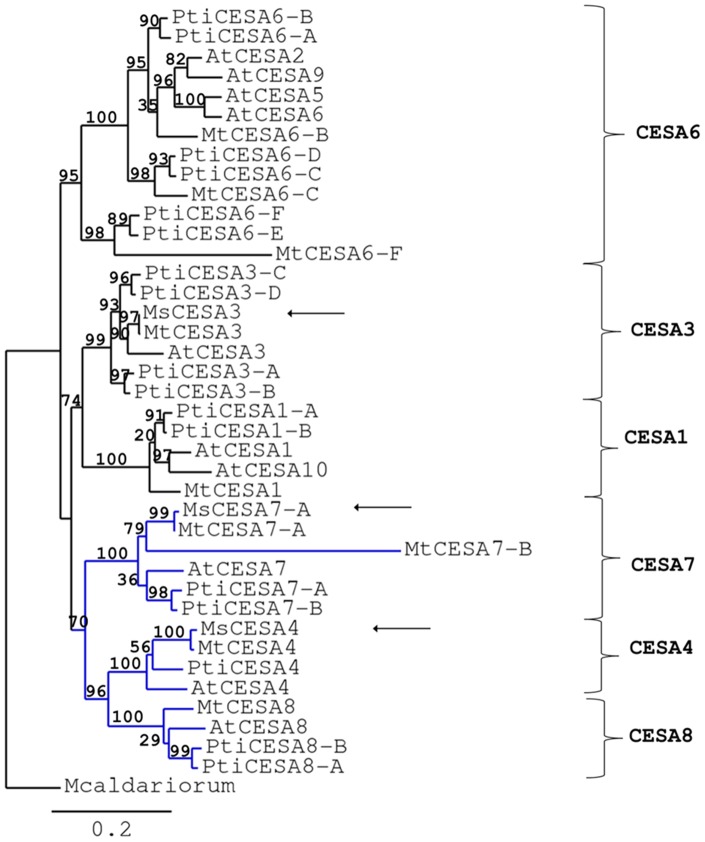
Phylogenetic relationships of CESAs from *M. truncatula*, *M. sativa*, *P. trichocarpa* and *A. thaliana* by maximum-likelihood analysis. Bootstrap = 100. Numbers indicate percentage of branch support values. The *scale bar indicates* an evolutionary distance of 0.2 amino acid substitutions per position*s*. *Mesotaenium caldariorum* CESA1 [GenBank: AAM83096] was used as outgroup to root the tree. The branch of secondary CESAs is indicated in blue. Arrows point to the three full-length CESAs identified in *M. sativa*.

*In silico* analysis of *M. truncatula* genome led to the identification of 9 putative *CesA* genes (Table 2). On the basis of the amino acid sequence identity with the orthologs from *A. thaliana* and poplar, a nomenclature is here proposed (Table 2) which follows the one recently proposed for *Populus* [46]. *M. truncatula* CESAs are between 981 and 1098 amino acids long and show from 6 to 8 transmembrane domains (TMDs; Table S3) according to the parameters of TMHMM [47]. The CESAs showing 6 TMDs actually display the occurrence of 2 additional potential TMDs, which however do not reach the critical threshold of the software (not shown). Therefore, since the CESAs so far described typically show the occurrence of 8 TMDs, the alfalfa proteins might as well all share the same feature. All the proteins show the occurrence of the signature motif typical of processive glycosyltransferases from family 2 (GT2s), i.e. D, D, DxD, QxxRW; MtCESA6-F, however, shows amino acid substitutions in the conserved motif (Fig. S3). The genes also have the zinc-finger domain (CxxC)4 (Fig. S3). Other genes with amino acid substitutions in the processive GT2s motif have been classified as CESAs (i.e. in *Cicer aretinum* and *Phaseolus vulgaris*) [GenBank: XP_004499618.1, ESW20735.1](Fig. S3), moreover phylogenetic and blast analyses both classify MtCESA6-F as a putative CESA and assign it to the primary CESAs clade (Fig. 5). Therefore this gene was assigned to the *CesA6* branch and retained for expression analysis.

**Table 2:** Proposed nomenclature for the *CesA* genes from *M. truncatula* based on amino acid identities with the orthologous proteins from *A. thaliana* and *P. trichocarpa*.

*Populus/Medicago* %identity	*P. trichocarpa*	*M. truncatula*	*A. thaliana*	*Arabidopsis/Medicago* %identity
87	*PtiCesA1-A* estExt_fgenesh4_pm.C_LG_XVIII0125	*MtCesA1* Medtr3g107520	*AtCesA1/RSW1* AT4G32410	84
87	*PtiCesA1-B* fgenesh4_pg.C_LG_VI001789	*MtCesA1* Medtr3g107520	*AtCesA1/RSW1* AT4G32410	84
83	*PtiCesA3-A* eugene3.00060479	*MtCesA3* Medtr3g030040	*AtCesA3/CEV1* AT5G05170	85
84	*PtiCesA3-B* eugene3.00160483	*MtCesA3* Medtr3g030040	*AtCesA3/CEV1* AT5G05170	85
88	*PtiCesA3-C* estExt_fgenesh4_pg.C_LG_IX0979	*MtCesA3* Medtr3g030040	*AtCesA3/CEV1* AT5G05170	85
88	*PtiCesA3-D* estExt_Genewise1_v1.C_LG_I1792	*MtCesA3* Medtr3g030040	*AtCesA3/CEV1* AT5G05170	85
84	*PtiCesA4* eugene3.00002636	*MtCesA4* Medtr2g035780	*AtCesA4/IRX5* AT5G44030	77
87	*PtiCesA6-B* estExt_fgenesh4_pg.C_LG_VII0650	*MtCesA6-B* Medtr8g092590	*AtCesA6/IXR2/PRC1* AT5G64740	82
81	*PtiCesA6-C* estExt_fgenesh4_pg.C_LG_V1107	*MtCesA6-C* Medtr1g098550	*AtCesA6/IXR2/PRC1* AT5G64740	74
68	*PtiCesA6-F* fgenesh4_pg.C_scaffold_133000012	*MtCesA6-F* Medtr3g007770	AtCesA6/IXR2/PRC1 AT5G64740	62
87	*PtiCesA7-A* estExt_Genewise1_v1.C_LG_VI2188	*MtCesA7-A* Medtr4g130510	*AtCesA7/IRX3* AT5G17420	85
63	*PtiCesA7-B* gw1.XVIII.3152.1	*MtCesA7-B* Medtr8g063270	*AtCesA7/IRX3* AT5G17420	64
80	*PtiCesA8-A* gw1.XI.3218.1	*MtCesA8* Medtr8g086600	*AtCesA8/IRX1* AT4G18780	76
77	*PtiCesA8-B* eugene3.00040363	*MtCesA8* Medtr8g086600	*AtCesA8/IRX1* AT4G18780	76

Loci are as reported in the Phytozome web portal [25].

Phylogenetic analysis showed the occurrence of 6 CESA clades with proteins involved in primary and secondary cell wall biosynthesis (Fig. 5). MtCESA1, MtCESA3, MtCESA6-B, MtCESA6-C, MtCESA6-F belong to the primary cell wall clade, while MtCESA4, MtCESA7-A, MtCESA7-B and MtCESA8 belong to the secondary cell wall clade (Fig. 5). Although MtCESA7-B and MtCESA6-F show the lowest % identity (Table 2), the phylogenetic tree classifies them as representatives of the CESA6 and CESA7 clade respectively (Fig. 5). The branches relative to these genes correspond to higher evolutionary distance (Fig. 5), a finding, which might indicate different roles with respect to their paralogs. Nevertheless the branch support values for the CESA6-E/F and CESA7-A/B clades are high (98 and 100%, respectively; Fig. 5).

**Figure 6:**
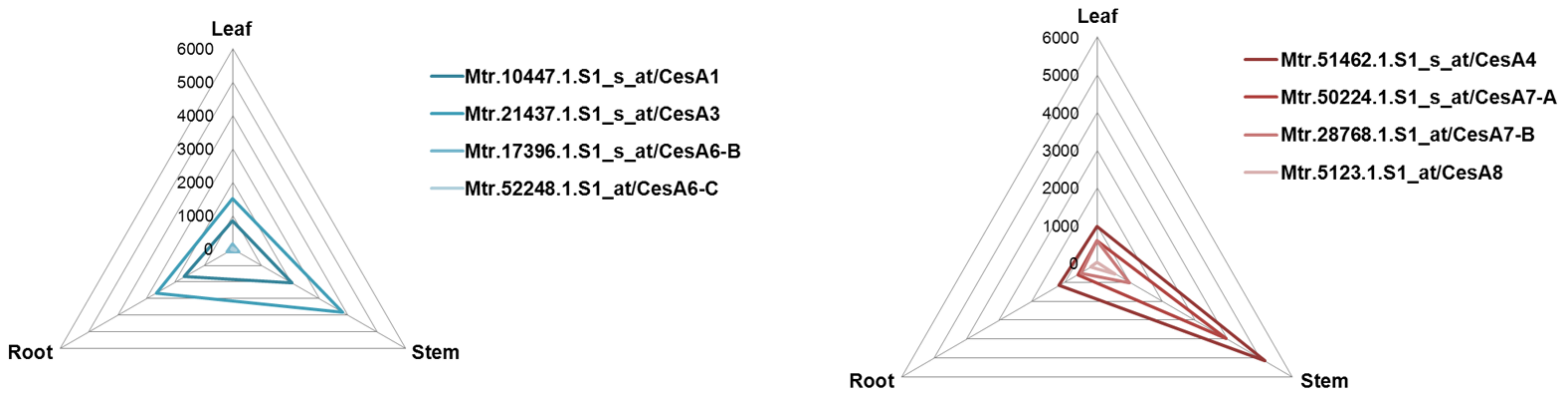
Radar plots of *M. truncatula CesA*s obtained plotting the microarray data retrieved at [31].

The phylogenetic analysis shows, as expected, that orthologous genes from different species are more related than homologs from the same species [48]. Some of the identified CESAs are represented by different genes in *M. truncatula*. Three orthologs of AtCESA6 are present: these genes might display specific roles in primary cell wall biosynthesis, but it is possible that they participate in secondary cell wall biosynthesis too, since poplar CESA6-E and CESA6-F were shown to be part of one of the two types of complexes found in differentiating xylem [49]. The CESA6 members group together with the *A. thaliana* CESA2, CESA5, CESA9: this finding reflects their possible interchangeability in the primary CESA complex [50]–[51]. In the secondary cell wall clade, the occurrence of 2 AtCESA7 orthologs is observed. This is especially interesting if one considers that the presence of 2 *CesA7* and *CesA8* genes is a reported feature for woody angiosperms such as poplar, where the biosynthesis of wood represents an important process. Further functional characterizations are necessary to unveil the role of the 2 CESA7 in *M. truncatula*. However, in the light of the specialization and promiscuity that the different CESAs display, e.g. mucilage or seed coat biosynthesis [52]–[54], involvement in both primary and secondary cell wall biosynthesis or formation of mixed complexes [51],[55]–[56], it is plausible to hypothesize that these 2 proteins co-participate in the assembly of secondary wall complexes and/or possess specific functions in cell wall biosynthesis. The tissue-specific expression of *M. truncatula CesA*s obtained from publicly available microarray data [31] confirmed the annotation of the genes into the primary and secondary clades: as can be seen from Fig. 6, the primary *CesA*s show a homogeneous expression in roots, leaves and stems, while the secondary display a higher expression in the stems. Notably the primary *CesA*s *MtCesA3* and *MtCesA1* show a high level of expression in the stems (Fig. 6 and Fig. S4), a finding which suggests a role for these genes in alfalfa stem cell wall biosynthesis.

Three full-length *CesA* sequences from *M. sativa* have been here obtained [GenBank: KJ398155, KJ398156, KJ398157; Fig. S1]; the phylogenetic analysis classifies them as MsCESA3, MsCESA4 and MsCESA7-A (Fig. 5). Partial sequences have been obtained for the other *CesA*s of *M. sativa* (Figs. S1 and S2).

### Cell wall-related genes from *M. sativa* show two main trends in response to abiotic stresses

**Figure 7:**
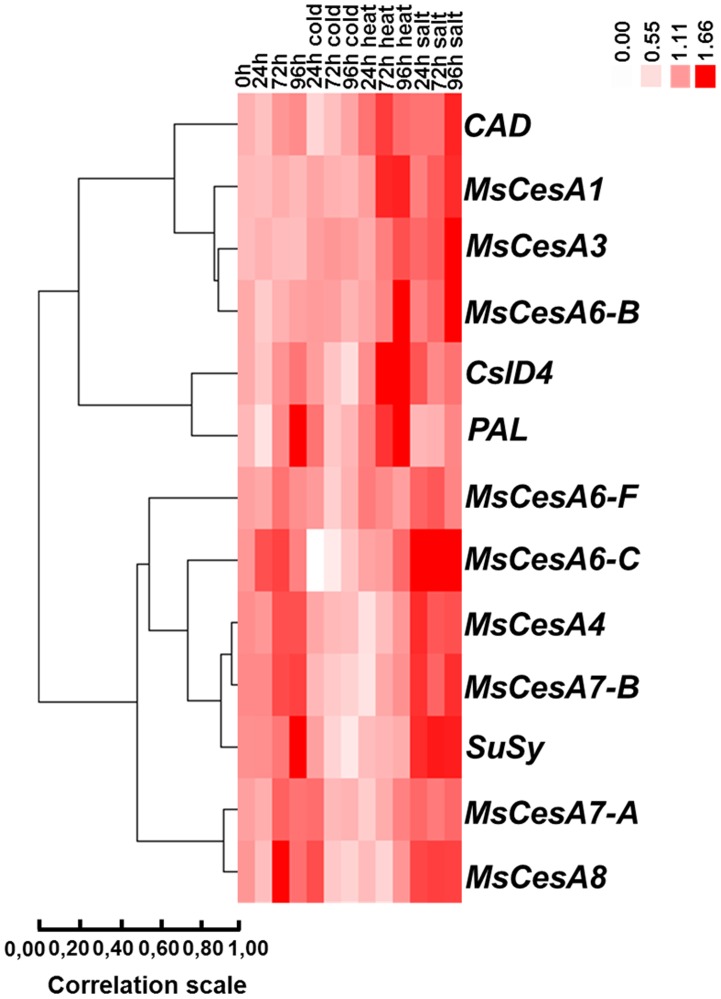
Heat Map representation of the data in Fig. S5 showing the hierarchical clustering of cell wall-related genes in response to the abiotic stresses at the different time points in alfalfa stems. The data collected refer to 3 independent biological replicates, each consisting of a pool of 15 plants. For each stress treatment a control group was always kept for the 24-48 h-96 h time points, for appropriate comparisons. The group clustering was generated with Cluster 3.0 [38] and visualized with Java TreeView [39], as described in Material and Methods.

Variations in the expression pattern in response to abiotic stresses can be observed among the different cell wall-related genes. From the Heat Map visualization, it is possible to discern two main groups: a heat/salt-induced and a cold/heat-repressed group of cell wall genes (Fig. 7). Salt/heat-induced genes are represented by the primary *CesA*s *MsCesA1*, *MsCesA3*, *MsCesA6-B* (with a Pearson correlation coefficient of 0.883) and to this group *CAD* belongs too (although with a lower correlation coefficient of 0.690). *CslD4* and *PAL* are also assigned to this group, although they cluster in a different branch, as their trend is less sharp than the one observed for primary *CesA*s (Fig. 7). The cold/heat-repressed group is represented by the secondary *CesA*s, together with *SuSy* (correlation coefficient of 0.91 for the cluster *SuSy*, *MsCesA4* and *MsCesA7-A*, and of 0.93 for *MsCesA4* and *MsCesA7-B*). The hierarchical clustering assigns to this group also *MsCesA6-C* and *MsCesA6-F* (Fig. 7). The statistical analyses carried out on the RT-qPCR data (Fig. S5) reveal that the changes in expression for *MsCesA1* and *MsCesA6-F* are statistically not significant; however their expression patterns can be interpreted as an overall trend which enables their classification in the heat/salt-induced and cold/heat-repressed group, respectively (Fig. 7).

A more detailed analysis of *MsCesA*s expression profiles shows mild but significant change for *MsCesA3*, with respect to the control, in response to salt stress after 96 h (Fig. S5; Table S4). *MsCesA6-B* displays an increase in expression at late stages of heat and salt application, which reaches a maximum after 96 h of treatment (Fig. S5; Table S4). *MsCesA6-C* shows a noteworthy decrease after 24 and 72 h of cold stress treatment (Fig. S5; Table S4).

*MsCesA4*, *MsCesA7-A and MsCesA7-B* show a trend towards decrease in expression already after 24 h of heat stress, while *CesA8* responds later, 72 h after the application of the stress (Fig. S5; Table S5). The correlation analysis of the wall-related genes performed with qBase^PLUS^ revealed a strong correlation between *MsCesA4* and *MsCesA7-A* in all the conditions tested (Fig. S6). This is not surprising, since these two genes belong both to the secondary CESAs clade, they are necessary for secondary cell wall biosynthesis together with CESA8 [57] and have been shown to interact in *Arabidopsis* [56].

## Discussion

The use of RT-qPCR for gene expression studies is a tool of unanimously recognized value, even in the current scientific era marked by the next generation sequencing revolution. Its utility is indeed unquestionable and necessary for validation of results massively produced via high-throughput methods.

For relative gene expression studies using RT-qPCR, the selection of suitable reference genes is a factor of paramount importance. Several studies in the literature have already undertaken the analysis of a set of candidate reference genes for normalization strategies in different plant species and conditions. Lists of stable genes are already available for relative RT-qPCR studies in plant tissues; however it is important to check their suitability in the experimental set-up adopted.

We have here identified and validated the use of reference genes for expression studies in alfalfa plants under different abiotic stresses. Two well-known and widely-used softwares, geNorm^PLUS^ [2] and NormFinder [33], have been chosen to rank the stability of the selected genes and we show that for some tissues, the best gene pairs identified differed between the 2 methods (Fig. 1 and Table 1). However, we were able to identify and propose a set of reference genes, ranked among the most stable by both softwares, namely eIF4A, PAB4, ADF1 and TFIIA. These genes can therefore be included in a panel of candidates to be tested for RT-qPCR studies in alfalfa and, potentially, in other leguminous plants.

For the validation phase, we have used as a model gene a plant kinase, *SK1*, known for its susceptibility to stresses [44] and we show that the response pattern is similar in the different tissues, where cold and heat stress cause the most pronounced responses, namely reduction and increase of expression, respectively (Fig. 3).

We have subsequently extended our RT-qPCR study to cell wall biosynthetic genes in stems, since our efforts are currently devoted towards understanding the regulation of cell wall biosynthesis dynamics in stems of alfalfa plants. In particular we here show the expression of nine putative *CesA*s, belonging to both primary and secondary wall clades (Fig. 5), together with other wall-related genes (Fig. 7). Although several reports in the literature have shown a link between cell wall biosynthesis/modification and abiotic stresses [58]–[61], a detailed investigation of cell wall gene expression changes in response to different abiotic stresses is lacking.

The main finding of our investigation is the elucidation of the wall-related gene dynamism in alfalfa plants subjected to abiotic stresses. The hierarchical clustering analysis identified two main trends in response to abiotic stresses: a salt/heat-induced and a cold/heat-repressed group of genes. Interestingly, a gene known to be involved in lignin biosynthesis, *CAD*, grouped together with the primary *CesA*s *MsCesA1*, *MsCesA3* and *MsCesA6-B* (Fig. 7): this indicates that these genes, although not strictly related, show a common response mechanism to abiotic stress. In this respect it should be noted that induction of a peroxidase, triggering in its turn an increase in lignin and suberin deposition, has been reported in tomato plants exposed to salt stress [62] and that tomato plants under salt stress show an increased number of lignified cells [63]. In addition to this, a link between miRNAs, abiotic stresses and lignification has been unveiled in *A. thaliana*, as miR397b, a miRNA targeting a laccase (and consequently affecting lignification), was shown to be up-regulated in response to salt stress [64]–[65]. *PAL* and *CslD4* also clustered with the *CAD*-primary *CesA*s group, although with a lower correlation: both genes display a heat and salt-stress responsive trend at later stages of treatment (Fig. 7; Fig. S5; Table S6). Cellulose synthase-like genes belong to the CESA superfamily and several members involved in wall glycan biosynthesis have been identified [66]. Many members of the *Csl* group of genes have not yet been functionally characterized, however representatives of the *CslD* clade are required for tip-growing cells [67] and *CslD1* and *CslD4* have been shown to affect cellulose biosynthesis in pollen tubes [68]. Moreover, another member of the *CslD* clade, *CslD5*, was shown to be required for osmotic stress tolerance in *A. thaliana* [60]. The results shown by the hierarchical clustering (Fig. 7) suggest that *PAL* and *CslD4* might be involved in cell wall remodeling in response to heat stress in alfalfa stems in a pathway likely involving increased lignin biosynthesis and cellulose deposition to strengthen the wall under the adverse condition. Heat stress triggers substantial modifications in plants: changes in ultrastructural anatomy and cell wall polysaccharide composition have been observed in coffee leaves subjected to heat stress, with an increase in monolignol content [69].

The susceptibility of primary *CesA*s to exogenous stresses is a known feature: the *A. thaliana cev1/CesA3* mutant shows constitutive expression of stress responsive genes, together with an increased resistance to fungal attack [70].

The second group of genes identified by the hierarchical clustering is represented by the secondary *CesA*s together with *SuSy*, *MsCesA6-C* and *MsCesA6-F* (Fig. 5). *MsCesA6-C* and *MsCesA6-F* belong to the primary *CesA*s and it is interesting that these genes cluster with secondary *CesA*s. This might indicate that, as already discussed for *CAD*, a similar response mechanism exists between these genes and the secondary *CesA*s, or it can indirectly show that they are more functionally related to secondary *CesA*s. This needs verification, however the presence of multiple *CesA6* genes in alfalfa might indicate overlapping and/or distinct roles in cell wall biosynthesis.

The second group of genes identified by the hierarchical clustering shows down-regulation in response to cold and heat stress. Heat stress is known to cause a decreased expression of *SuSy* in pollen grains (accompanied by a decrease in the expression of other wall-related genes and vacuolar invertases; [71]) and in chickpea leaves [72]. The RT-qPCR analysis performed on alfalfa stems suggests that the decrease observed in secondary *CesA*s expression upon cold and heat stress treatment might be related to an impaired fueling of UDP-glucose by *SuSy*, which, despite not strictly required for cellulose synthesis [73]–[74], might contribute to increase the rate of synthesis, by concentrating the substrate [74].

## Conclusions

The present work constitutes a useful guide for the identification of appropriate reference genes in expression studies on alfalfa, which can be extended to other legume crops for analysis. Through analyses using NormFinder and geNorm^PLUS^, we have identified a set of suitable candidates, which can be included in a panel of reference genes to be tested for differential expression analysis.

The results concerning *CesA*s and a few other wall-related genes confirm an active role played by the cell wall in response to exogenous stimuli and constitute a step forward in delineating the complex pathways fine-tuning the response of plants to abiotic stresses.

## Supporting Information

Figure S1 Nucleotide sequences of *MsCesA*s. Sequence details of the *CesA*s identified in alfalfa.
(DOC)

Figure S2 Alignment of alfalfa partial *CesA* sequences with *M. truncatula CesA*s. Alignment of the *CesA*s from alfalfa with the respective orthologs from *M. truncatula*.
(DOC)

Figure S3 Sequence details of *MtCESA6-F*. Alignment of *MtCESA6-F* with *CesA*s from *C. aretinum* [GenBank: XP_004499618.1] and *P. vulgaris* [GenBank: ESW20735.1] showing the amino acid substitutions in the processive GT2s motif (bold and underlined). The zinc-finger domain (CxxC)4 is highlighted in yellow.
(DOC)

Figure S4 Electronic Fluorescence Pictographic (eFP) representations of *M. truncatula CesA1*, *CesA3*, *CesA6-B*, *CesA6-C*, *CesA4*, *CesA7-A*, *CesA7-B*, *CesA8*.
(TIF)

Figure S5 Gene expression profiles of cell wall-related genes in stems of alfalfa plants subjected to abiotic stress. Data were normalized using eif4A/TFIIA. Means sharing a letter are not significantly different at α = 0.05. NRE indicates Normalized Relative Expression.
(TIF)

Figure S6 *MsCesA7-A* and *MsCesA4* relationship. Correlation between *MsCesA7-A* and *MsCesA4* in stems under abiotic stress conditions. Pearson (log) r = 0.962; Spearman (log) r = 0.961.
(TIF)

Table S1 List of primers used to amplify *MsCesA*s. Name of the primers, with the respective sequences, used to amplify the *CesA*s from *M. sativa*.
(DOC)

Table S2 List of primers used for the RT-qPCR study. Name of the primers used for the RT-qPCR study, with the respective sequences. Details concerning the amplicons details (length, Tm), PCR efficiencies and regression coefficients are included.
(DOC)

Table S3 CESAs from *M. truncatula*. Details concerning number of predicted transmembrane helices (TMHs, according to [47]) and the length of the putative CESAs from *M. truncatula*.
(DOC)

Table S4 Normalized Relative Expression for primary *CesA*s. Normalized Relative Expression values ± standard deviation and significance (Sig.) for the primary *CesA*s. Data were normalized using eif4A/TFIIA.
(DOC)

Table S5 Normalized Relative Expression for secondary *CesA*s. Normalized Relative Expression values ± standard deviation and significance (Sig.) for the secondary *CesA*s. Data were normalized using eif4A/TFIIA.
(DOC)

Table S6 Normalized Relative Expression for for *CAD*, *CslD4*, *PAL* and *SuSy*. Normalized Relative Expression values ± standard deviation and significance (Sig.) for *CAD*, *CslD4*, *PAL* and *SuSy*. Data were normalized using eif4A/TFIIA.
(DOC)
