# Supplementary material for: Alfalfa Cellulose Synthase Gene Expression under Abiotic Stress: A Hitchhiker’s Guide to RT-qPCR Normalization
Source: PLoS One. 2014 Aug 1;9(8):e103808. doi: 10.1371/journal.pone.0103808 (PMC4118957; doi:10.1371/journal.pone.0103808)
Supplement: Figure S1 — Nucleotide sequences of MsCesAs. Sequence details of the CesAs identified in alfalfa. (DOC) [file pone.0103808.s001.doc]

***MsCesA3* KJ398155**

ATGATGGACTCAGAAGGAGAAGCTGGGGATAAGCCGATGAAGACGTTGGGTAGCAAAGTCTGTCAGATTTGTGGTGATAATATCGGTAGTGCTGTTAATGGCGATCCGTTCATTGCTTGTGGTGTTTGTGCCTTCCCGGTTTGTAGGCCGTGTTATGAGTATGAAAGGAAAGATGGGAATCAGTCTTGCCCACAGTGCAAAACTCGATACAATAAGCACAAAGGTAGTCCTGCAATTCTTGGAGATCGTGAAGAGGATGGTGGTGCTGATAATGATGCCAATGACTTCAAGTACAATTCAGAAACTCAGACCCAAAAGCAAAAGATCGCAGAACGCATGTTGAGTTGGCAAATGGCGTATGGGCGAGGTGAGGAGGTTGATGCTCCAAATTATGATAAGGAAGTTTCTCACAATCACATTCCTCGGCTAACCGGTGGACAAGAGACATCTGGAGAATTATCTGCAGCCTCACCTGAGAGGATGTCAATGGCATCTCCTGTAAATGCTCGCGGGAAACGAGTTCATAATCATCCATCATATTCGTCTGATCTTAATCAATCACCAAACATCAGGGTTGCTGAACCAGGATTGGGTAATGTAGCTTGGAAAGAAAGAGTTGATGGCTGGAAAATGAAGCACGATAATAAGAATACTGCTCCAATGAGCACAGGTCAAGCTACTTCTGAAAGAGGAATAGGAGATATTGATGCCAGTACTGATGTGCTTTTCGATGATTCCTTGTTGAATGATGAAGCTCGACAACCACTTTCGAGGAAGGTTTCTATTCCATCTTCTAGGATAAATCCATACCGTATGGTCATTATTCTGCGGCTTGTTATCCTTGTCATTTTCTTGCACTACCGAATTACAAATCCCGTACCCAATGCTTATGCGTTGTGGTTAATATCAGTTATATGTGAAGTTTGGTTTGCCTTTTCTTGGATATTGGATCAGTTCCCCAAATGGCTTCCTGTGAACCGTGAAACATATCTGGACAGGCTTTCATTAAGATATGATCGGGAAGGGGAACCTTCACAGTTAGCAGCCGTTGACATTTTCGTCAGTACTGTTGATCCGTTAAAGGAGCCCCCAATTGTGACAGCCAATACTGTACTATCAATTCTTGCTGTTGACTACCCAGTGGATAAGGTCTCATGTTATGTCTCTGATGATGGTGCTGCTATGTTGACATTTGAAGCTCTTGCCGAGACATCAGAATTTGCAAGGAAATGGGTTCCTTTCTGCAAGAAATATGAAATTGAACCACGAGCACCTGAGTGGTACTTCTCAAAGAAAATTGACTACTTGAAAGATAAGGTCCAAGCATCATTTGTCAAAGATCGTAGAGCAATGAAGAGAGAATATGAAGAATTCAAAATTCGTATCAATGGACTTGTTGCAAAAGCAGTGAAAGTCCCTGAAGAAGGATGGGTGATGCAAGATGGTACACCTTGGCCTGGAAACAACACCAGAGACCATCCAGGAATGATCCAGGTTTTCTTGGGCCAAAGTGGAGGACTTGATACTGATGGTAATGAACTTCCACGTTTAGTCTATGTTTCTCGTGAAAAGCGTCCAGGGTTCCAACATCACAAGAAGGCCGGTGCCATGAATGCACTTGTTCGTGTATCAGCTGTGCTTACTAATGGACCTTTCTTATTGAATCTTGATTGTGATCATTACATAAACAACAGCAAAGCCTTGAGGGAAGCTATGTGTTTTATGATGGATCCCAACCTTGGAAAAAATGTTTGCTATGTTCAATTTCCACAGAGGTTTGATGGTATTGATAGGAACGATCGATATGCCAATCGTAATACTGTTTTCTTTGATATAAACTTGAGAGGGTTGGATGGCATTCAAGGTCCCGTTTATGTGGGTACTGGATGTGTCTTCAATAGAACTGCTTTATATGGTTATGACCCTCCTATCAAACCCAAGCATAAAAAGCCTAGTCTTGTTTCTTCACTTTGCGGTGGAGATCGAAACAAGAGCTCAAAATCTAGCAAGAAAGACTCAAAAAAGAATAAATCTAGCAAGCATGTTGATCCAACTGTGCCCGTCTTTAGTCTAGAGGATATAGAAGAAGGGGTGGAAGGTGCTGGATTTGATGATGAAAAATCACTACTCATGTCACAAATGAGCCTTGAGAGAAGGTTTGGTCAGTCTGCTGTTTTTGTTGCCTCTACACTTATGGAAAATGGTGGCGTTCCTCAGTCTGCAACTCCAGAAACTCTTCTCAAGGAGGCAATTCATGTTATCAGTTGTGGTTATGAAGATAAATCAGAATGGGGAACTGAGATTGGATGGATCTATGGTTCTGTCACAGAAGATATTCTTACTGGATTTAAGATGCACGCCCGTGGTTGGAGGTCTATATATTGCATGCCCAAGCTTGCTGCATTTAAAGGTTCAGCTCCTATCAATCTTTCCGATCGTTTGAACCAAGTGCTTCGATGGGCTTTAGGTTCAGTGGAAATTCTACTAAGTCGACATTGTCCCATCTGGTACGGTTATAGTGGAAGGTTAAAGTGGCTTGAGAGGTTTGCGTATATAAACACCACAATCTATCCAATCACTTCCATTCCTCTTCTCATGTATTGTACCTTACCTGCCGTCTGTCTCCTGACTAACAAGTTCATTATTCCACAGATTAGTAACATTGCAAGTATATGGTTCATATCTCTCTTTCTTTCCATCTTTGCAACTGGTATCCTTGAGATGAGGTGGAGTGGTGTTGGAATTGATGAATGGTGGAGAAATGAACAATTTTGGGTTATTGGTGGTGTTTCAGCTCATCTTTTTGCTGTGTTCCAAGGTTTACTCAAAGTACTTGCTGGAATTGACACAAACTTCACTGTTACCTCAAAAGCTTCAGATGAAGATGGAGACTCCGCAGAACTATACATGTTTAAATGGACAACACTTCTCATTCCACCAACAACTCTTCTCATTATAAACCTTGTCGGAGTTGTTGCAGGAATCTCTTATGCTGTTAACAGTGGCTACCAATCATGGGGACCACTCTTTGGTAAACTTTTCTTTGCATTTTGGGTGATCATCCATCTCTACCCCTTCCTTAAAGGTCTTATGGGACGCCAGAACCGAACGCCAACCATCGTGGTTGTTTGGTCCATTCTTCTTGCATCCATTTTTTCACTTTTATGGGTTCGAATCGACCCTTTTACGACAAGAGTCACTGGTCCCAAATCTGAGATGTGTGGAATCAACTGCTAG

***MsCesA4* KJ398156**

ATGGCTGGCTTGATCACTGGCTCTAATTCTCATTTCTCACATGACTCTGATGAGCATAAGCCTCCTCCTGCTAATAAATCCTCATCGAAAATATGTCGAGTTTGTGGTGATGAGATTGGATATAAGGAAAATGGAGAGCTTTTTGTGGCTTGTCATGTGTGTTCATTTCCAGTTTGTAAACCTTGTTATGAGTATGAGAGGAGTGAAGGGAATCAATGTTGTCCTCAATGCAACTCTCGCTATAAGCGCCATAAAGGTTGTCCAAGAGTGGTTGGAGATGAAGATGAGAACCTTGATGGAGATGATTTTGAAGATGAGTTTCCGGTCAAGAATCATCATGATGATCTAGACCAGAACCGCGATGTTAATCATGTGGAAAGTGTGGATTACAATCAACAGAAATTGCATACTTTTTCTTCAGCAGGAAGTGTTACTGGCAAGGATTTGGAAGGGGAGAAAGAATTCTACAGTAATGAAGAATGGCAAGAAAGAGTAGAGAAATGGAAAGTAAGACAAGAAAAGAGAGGTCTGCTAAACAAAGAGGATGGTAAAGAAGACCAAGGTGAAGAAGATGAGTACCTTATGGCTGAAGCAAGACAACCATTATGGCGCAAAGTTCCAATACCATCAAGCTTAATAAATCCATACCGAATAGTAATTGTGATGAGACTTGTAATCCTAGCTTTCTTCTTCCGTTTCCGTATCTTAACACCTGCATATGATGCATACCCTCTATGGCTCATATCAGTAATATGTGAAATATGGTTTGCACTATCATGGATACTTGATCAGTTTCCAAAATGGCTACCAATCACACGCGAAACTTACTTAGACCGTTTATCTATAAGATTTGAACGTGAAGGCGAACCAAACCAGCTTTCGCCTGTTGATGTTTTTGTTAGTTCTGTTGACCCTTTGAAAGAACCGCCTATTATAACAGCAAACACTGTTCTGTCCATACTTTCTGTTGACTACCCTGTTGAGAAAGTTACATGCTATGTATCAGATGATGGTGCTTCTATGCTTTTGTTTGATTGTTTGGCTGAAACTTCTGAGTTTGCTCGAAGGTGGGTTCCTTTTTGTAAAAAGTATAGTATTGAACCAAGGGCACCTGAGTATTACTTTAATGAGAAGATTGATTACTTGAAGGATAAGGTTGAGCCTACTTTTGTCAAGGAAAGAAGGTCTATGAAGAGAGAATATGAAGAATTTAAAGTGAAAATTAATGCTTTGGTGGCAAAGGCTCTGAAGAAACCTGAGGAGGGTTGGGTTATGCAAGATGGAACTCCATGGCCTGGTAACAACACTCGTGATCATCCAGGGATGATTCAGGTATACTTAGGAAGTGCTGGTGCACTAGACGTGGAAGGCAAGGAACTGCCAAAACTCGTGTATATTTCACGTGAAAAACGTCCCGGTTATCCACATCACAAGAAAGCCGGTGCCATGAATGCTTTAGTTCGAGTTTCTGCTGTGCTTACAAATGCACCATTCATGTTGAATCTCGATTGTGATCACTACATCAACAATAGCAAGGCTATTAGAGAGGCCATGTGCTTCCTAATGGATCCTCAACTAGGGAAGAAACTCTGCTATGTCCAATTTCCTCAAAGATTTGATGGTATTGATCGTCACGATCGATATGCTAATCGCAACACTGTCTTCTTTGATATCAATATGAAAGGGCTTGATGGTATTCAAGGTCCAGTGTATGTCGGTACCGGAACTGTGTTCAACAGACAAGCATTGTATGGATATGATCCACCAGTTTCCGAGAAAAGGCCAAAGATGACATGTGATTGTTGGCCTAAATGGTGTTGCTTTTGTTGTGGTTCAAGGAAAACCAAATCAAAAAAGAAGTCAGGAACAAATGGAAGAAGTCTCTTTAGTAGATTGTACAAGAAGAAGAAAATGGGAGGAAAAGATTATGTTAGAAAAGGATCTGGTTCCATGTTTGATCTCGAAGAGATTGAAGAAGGACTTGAAGGGTACGAGGAGCTAGAAAAGTCAACACTCATGTCACAGAAAAACTTCGAGAAGCGATTCGGTCAGTCTCCGGTTTTCATTGCTTCAACTTTGATGGAAAATGGAGGACTTCCTGAAGGCACAAATACACAATCATTGGTTAAGGAAGCCATTCATAACATAAGTTGTGGCTATGAAGAGAAGACTGACTGGGGAAAAGAGATTGGATGGATTTATGGCTCAGTCACAGAAGATATTTTGACTGGTTTCAAAATGCATTGCCATGGTTGGCGGTCTGTGTATTGTATCCCTAAGCGGCCTGCTTTCAAGGGGTCTGCACCTATCAATCTGTCAGATAGGTTGCACCAAGTTCTGAGATGGGCTCTAGGTTCTGTTGAGATTTTCCTTAGTCGCCATTGTCCGTTGTGGTATGGTTATGGAGGAAAACTAAAGTATCTTGAGAGGTTGGCTTATACTAATACCATTGTTTATCCATTCACTTCAATCCCTTTGCTTGCATACTGCACAATTCCAGCTGTGTGTCTCCTCACTGGAAAGTTCATCATTCCTACTCTGACCAACCTTGCTAGTATTTGGTTTATGGCACTATTTATCTCCATCATTTTAACCGGTGTACTCGAGCTTCGATGGAGTGGAGTTGCGATCGAAGACTGGTGGAGAAACGAGCAATTCTGGGTGATTGGAGGTGTATCAGCACATCTTTTTGCAGTGTTTCAAGGTTTCCTCAAAGTTCTAGCTGGAGTAGACACAAACTTTACTGTCACAGCAAAAGCAGCAGATGATGCTGAATTCGGAGAGTTATATCTCTTCAAATGGACAACACTTCTCATTCCACCAACCACTCTTATAATCTTGAACATTGTTGGCGTTGTTGCTGGTGTCTCTGATGCTATTAACAGTGGTAGTGGTTCATGGGGACCTTTATTTGGGAAGCTATTCTTTGCATTTTGGGTCATTGTTCATTTATATCCTTTCCTTAAAGGTCTTATGGGAAAACAAAACAGGACTCCTACTATTGTGGTACTTTGGTCAATCCTTTTGGCATCAATTTTCTCATTGATTTGGGTTAGGATTGATCCTTTCTTGCCTAAGCAAACTGGTCCCATTCTCAAACAATGTGGTGTAGAATGCTGA

***MsCesA7-A* KJ398157**

ATGGAAGCCAGCGCCGGACTAGTCGCTGGTTCTCACAACCGGAACGAGCTTGTTGTCATTCATGGCCATGAAGAGCACAAGCCTTTGAAGAACTTGGACGGTCAAGTGTGTGAGATATGTGGTGATGATGTTGGACTTACTGTGGATGGAGATTTGTTTGTGGCATGTAACGAGTGTGGTTTTCCGGTGTGCCGGCCTTGCTATGAGTATGAAAGAAGGGAGGGGAGACAACTTTGTCCTCAGTGCAAGACCAGATACAAGCGTCTCAAAGGGAGCCCTCGGGTTGAGGGAGACGATGATGAGGAGGATGTGGACGATATTGAACATGAATTCAAGATTGAAGACAAAATGAACAATCATGATCATTCTGCTGAGGCCATGCTGCATGGGAAGATGAGCTATGGAAGAGGTCCTGAAGATGACGAGAATGCACACTTTCCGGCTGTTATTGCTGGCGGTCGTTCTCGGAATGTGAGTGGTGAGTTCCCAATATCATCTCATAGTTATGGGGAGCAGATGCTATCTTCACTGCATAAAAGAGTGCATCCATATTCCGCTTCCGATCCTCGAAATGCAGGGTGGGATGAAAGGAGAGAAGATGGATCATATGATAGAATGGATGACTGGAAATTGCAGCAAGGAAATTTGGGACCTGAACCTGATGAAGATCTAGATGCAAACATGTCAGATGAAGCAAGACAACCACTGTCAAGGAAGGTACCAATAGCATCTAGCAAAATCAATCCATATAGGATGGTGATCGTGGCACGGCTTGTTATTCTTGGCTTCTTCCTCCGATACAGACTTATGAACCCAGTACATGATGCAATGGGGCTATGGCTAACCTCAATTATATGTGAAATCTGGTTTGCTATATCATGGATCCTTGATCAGTTCCCCAAATGGTATCCTATTGATAGAGAAACATACCTTGATCGTCTTTCACTCAGGTATGAGCGTGAAGGCGAACCCAATATGCTTGCTCCTGTAGATGTGTTTGTTAGTACTGTGGATCCCTTGAAGGAACCTCCTCTGAATACAGCCAACACAGTTCTTTCAATCCTGGCAATGGACTACCCCATTGATAAGATATCATGCTACATTTCTGATGACGGAGCTTCAATGTGTACATTTGAAGCCCTGTCGGAAACGGCAGAGTTTGCTAGGAAATGGGTACCATTTTGTAAGAAATTTTTGATAGAACCTCGTGCACCAGAGATGTACTTCTCTGAGAAAATTGACTATCTAAAGGACAAGGTGCAGCCAACTTTTGTCAAAGAACGTCGGTCCATGAAGAGAGAATATGAAGAGTTTAAGGTTAGGATCAATGCACTTGTGGCAAAAGCTCAAAAGGTTCCTGCAGGAGGGTGGATTATGCAGGATGGGACACCATGGCCAGGAAACAATACTAAGGATCATCCTGGTATGATTCAAGTTTTTCTTGGTCACAGTGGAGGTCATGATAGTGAAGGAAACCAGCTTCCTCGCCTTGTTTATGTATCTAGAGAGAAAAGGCCAGGATTTCAACACCACAAGAAAGCCGGTGCCATGAATGCTCTGGTACGGGTCTCTGCAGTGCTTACAAATGCTCCTTTCATGCTGAACTTGGATTGTGATCATTATATCAATAACAGCAAGGCTGTCCGAGAGGCCATGTGCTTCTTGATGGACCCCCAAACAGGGAAGAAAGTCTGCTATGTCCAGTTTCCTCAAAGATTCGATGGCATTGATGCGCATGATCGATATGCTAATAGAAACACAGTTTTCTTTGATATTAACATGAAGGGTCTAGATGGTATTCAGGGTCCTGTATATGTCGGCACAGGGTGTGTATTTAGAAGGCAGGCTTTATATGGATATAATCCTCCTAAGGGTCCCAAGCGTCCAAAAATGGTAAGTTGTGATTGCTGCCCATGTTTTGGAAGGCGCAAGAAGGTTAAGCATGCAATGAACGACGCAAATGGAGAAGCTGCAGGCCTAAGAGGAATGGAAGATGACAAAGAGTTACTGATGTCCCAGATGAATTTTGAGAAGAAATTTGGGCAGTCATCAATTTTTGTGACTTCAGTCTTGATGGAAGAGGGTGGTGTACCTCCTTCGTCAAGTCCAGCAAGCCAACTTAAAGAAGCCATTCATGTAATCAGTTGCGGATATGAAGATAAAACTGAATGGGGGATTGAGCTTGGTTGGATTTATGGGTCCATTACAGAGGATATTTTGACAGGCTTTAAGATGCATTGCCGTGGTTGGAGATCCATTTACTGTATGCCAAAGAGAGTAGCATTCAAGGGTACTGCTCCTATCAACTTGTCAGATAGACTCAACCAGGTGCTTCGTTGGGCTCTTGGCTCCATTGAGATCTTCTTCAGTCATCATTGCCCTTTATGGTATGGCCACAAGGAAGGGAATCTGAAGTGGCTGGAGCGATTTGCCTATGCAAACACAACCGTCTACCCCTTCACCTCCATACCTCTAGTTGCCTACTGTATTCTTCCAGCTGTCTGCTTACTCACTGACAAATTCATCATGCCACCGATAAGCACTTTTGCAAGTTTGTACTTTGTTGCTCTCTTCTCTTCAATCATGGCAACAGGCATTCTTGAGTTGAAATGGAGTGGAGTCAGCATTGAGGAATGGTGGAAAAATGAGCAATTCTGGGTCATTGGTGGTGTATCACCACATCTCTTTGCTGTCATACAAGGGCTTCTGAAGGTTCTTGCTGGAATTGACACCAACTTCACTGTTACATCCAAGGCAACAAATGATGAGGAATTTGGAGAATTATACGCCATCAAGTGGACCACTCTCCTAATTCCTCCAACTACTATCTTAATAATCAATATTGTTGGGGTTGTTGCTGGAATCTCAAACGCCATAAACAATGGTTACCAATCATGGGGACCTCTATTTGGAAAACTCTTCTTTTCCTTCTGGGTGATTGTCCATCTATATCCATTCCTTAAAGGTTTGATGGGTCGGCAAAACCGCACCCCCACCATTGTTGTGATATGGTCAGTGTTGTTGGCTTCCATTTTCTCTTTGTTATGGGTAAGAATTGACCCATTCGTGATGAAAACTAAGGGACCTGATACCAAGCTATGCGGAATCAACTGTTAA

***MsCesA1***

AATAATAATAAGTCAAGGAGACAGTGGGATGATTCAGACAGATCAGCTTCATCTTCTAGACGCGAATATCAGCAACCTCCTCTTCTCACCAATGGCCAAACTATGTCTGGCGAGATNCCTACACCTGATAATCAATCTGTCCGAACTACTTCTGGTCCTTTGGGCCCATCTGAGAAAGCTCACTCACTTCCCTATATTGATCCAAGGCAACCACTTCCGGTGAGAATTGTGGATCCATCAAAGGACTTAAACTCATATGGTTTGGGAAATGTTGACTGGAAGGAAAGGGTTGAAGGTTGGAAGCTGAAGCACGAGAAAAATATGGTACAGATGACTGGTAGATATGCTGATGGGAAAAGCGGAGGAGGAGATATTGAAGGGACTGGTTCTAATGGAGAAGAACTTCAAATGGTTGATGATGCTCGACAACCTATGAGTCGGATTGTACCCATTTCTTCATCTCAGCTGACCCCTTATCGTGTTGTCATCGTGTTCCGGCTGATAGTTCTTGGTTTCTTCTTGCAATATCGTGTAACTCACCCGGTAAAAGATGCTTACCCACTGTGGTTGACTTCAGTTATCTGTGAGATTTGGTTTGCATTTTCCTGGATTTTGGATCAGTTTCCAAAATGGTCTCCCATTAACCGTGAGACTTATCTGGAGAGGCTTGCCATAAGATATGATCGTGATGGAGAACCATCACAGTTGGCTCCTGTCGACGTATTTGTCAGTACAGTGGACCCTCTCAAAGAGCCACCTATTGTAACTGCAAACACTGTTTTGTCTATACTCGCTGTTGACTACCCTGTCGACAAGGTTTCTTGCTATGTATCTGATGATGGTTCAGCTATGTTGAGTTTTGAAGCCTTATCTGAAACAGCCGAGTTTGCAAAGATGTGGGTGCCCTTTTGCAAAAAACACAGTATTGAGCCAAGAGCACCTGAATTTTATTTTCTTCAGAAGATTGATTACTTAAAGGACAAGGTTCAACCCTCTTTTGTTAAGGAGCGACGAGCAATGAAGAGACAATATGAAGAATTCAAAGTAAGGATCAATGCCTATGTTGCTAAAGCTCAGAAGATGCCAGAGGAAGGTTGGACAATGCAGGATGGAACTCCTTGGCCTGGAAATAATCCCAGGGATCATCCTGGAATGATTCAGGTGTTCTTAGGACATAGTGGAGGGCTTGATACAGATGGCAATGAACTTCCCAGGCTTGTTTATGTGTCTCGTGAAAAGCGACCAGGCTTCCAACATCACAAGAAGGCTGGAGCTATGAATGCTTTGATTCGAGTTTCTGCTGTCTTGACCAACGGTGCATATCTTTTGAATGTCGATTGTGATCATTATTTCAATAATAGCAAAGCTCTTAAGGAGGCTATGTGTTTCATGATGGATCCTGCTTATGGAAAGAAGACATGCTATGTTCAATTTCCGCAGAGATTTGATGGCATTGATTTGCACGATCGATATGCCAATCGCAATATTGTCTTCTTCGATATCAACTTGAAAGGTCAGGATGGTATTCAGGGCCCTGTCTATGTGGGAACTGGTTGCTGTTTCAATAGGCAAGCTTTGTATGGTTATGATCCTGTTTTGACTGAGGAAGATCTCGAACCTAACATTATTGTTAAGAGTTGTTGGGGTTCTAGAAAGAAAGGAAAGGGTGGGAATAAGAAGTACGGTGACAAGAAGAGGGGAGTTAAAAGAACTGAATCCACCATTCCCATATTTAATATGGAGGATATAGAGGAGGGTGTCGAAGGTTATGATGATGAAAGGTCACTTCTAATGTCTCAAAA

***MsCesA6-B***

TTGCAAGTCTTGTGTTCATGGCCCTCTTCATATCCATCGCAGCAACCGGTATCCTTGAGATGCAATGGGGTGGTGTTGGAATAGATGATTGGTGGAGGAATGAACAGTTTTGGGTGATTGGAGGTGCTTCATCACATTTTTTTGCCCTTTTCCAAGGTTTGCTCAAGGTTTTAGCTGGTGTCGACACAAACTTCACTGTTACATCAAAAGCAGCCGACGATGGAGAATTCTCGGAGCTCTACGTATTCAAATGGACTTCGCTATTAATCCCTCCAATGACGCTATTAATCATGAATATTGTGGGCGTGATTGTTGGTGTCTCCGATGCGATCAATAATGGTTATGACTCATGGGGACCTCTGTTCGGTAGATTATTCTTTGCCCTTTGGGTTATCATACATCTTTATCCATTCCTCAAGGGTTTGCTTGGGAAACAGGATAGGATGCCAACCATTGTTTTGGTTTGGTCAATCTTGCTAGCTTCCATCTTGACTCTCTTGTGGGTTAGAGTTAACCCTTTTGT

***MsCesA6-C*** ATGGACACTAATGGAAGATTAGTTGCAGGATCACATAACAGGAATGAGTTTGTTCTTATCAATGCTGATGACACTGCAAGAGTGAATGCTGTGACAGAATTGAGTGGACAAATTTGCCAGATCTGCGGGGATGAGATAGAGCTTACAGTGGATGATGAACCTTTTGTTGCTTGCAATGAATGTGCATTCCCTGTGTGTAGACCCTGCTATGAGTATGAAAGAAGAGAAGGGAATCAAGCTTGTCCTCACTGCAAAACTAAATACAAACGCATAAAGGGTAGTCCCAGAGTTGAGGGTGATGAAGAAGAGGACGGTATTGATGATTTGGAAAATGAGTTTGACATTGGAAGCAATATCAAACATGACTCTCATCACATTACCGATGCTATGTTCTTCGCTCGCCTCAACAATATTGGCCAGAGTTCACTAATGAATGCTTCAGGAATCACTACACCATCGGAGTTTGATGCAGCTTCTATGGCTGCTGATATACCCTTCCTGACATACGATCATGAGGATCTTGGAATTTCTTCTGATAAACATGCTCTGATTATCCCTCCGTGCAAGCCCCACGGGAAACGGGTTCATCCTATGCCTTTTCCTGATTCATTTGTGCCGGTTCTACCAAGACCTATGGATCCTAATAAAGATTTGGCTGTTTATGGCTATGGAAGTGTTGCATGGAAAGAAAGAGTGGAGGAATGGAAGAAAAGGCAGAATGAAAAATTAGAGGTGGTTAAGCACGGGGTTGATAACAATGTTGATGAGTTTAATGATCCCGATTTGCCAAAAATGGATGAA

***MsCesA6-F***

ATCCGTACCGTATGATCATAATAATAAGACTGATTGTTCTTGGATTCTTCTTCCAATATAGAATTATGAATCCAGTAGACAATGCATATGCTTTGTGGCTTGTGTCAGTAATATGTGAGATTTGGTTCACTCTTTCATGGATTCTTGATCAGTTCTCAAAGTGGTTTCCTGTCATGAGGGAAACCTATCTTGACAGGCTTTCCTTAAGGTATGAAAAGGAAGGTCAACCATCACAACTTTCACCAATTGATATATTTGTGACTACAAATGATCCATTAAAAGAGTCTCCTCTAGTGACAGCAAACACAGTTCTATCAATTCTAGCAATAGATTACCCTGCTGAAAAGGTGTCATGTTATGTTTCTGATGATGGAGCAGCAATGTTAACATTTGAGGCTTTATCTGAAACTTCTGAATTTGCAAGGAAATGGGTTCCTTTTTGTAAGAAGTTCAACATTGAACCTAGAGCTCCAGAATGGTATTTTGCTGAGAAAATAAATTATTTAAAAGATAAGGTTCATTCATCATTTGTGAAAGAGAGAAGAGCAATGAAGTTGCAAAGGCTAAGAAGGTTCCAGAAGAAGGGTGGACAATGCAAGATGGAATGTTGTGGCCTGGAAATAATATTCGTGACCATCCAGGAATG

***MsCesA7-B*** AATTGGACTTTGGTTCATATCAGTATCATGTGAAATCTGGCTTGCATTGTCATGGATACTTGATCAGATTCCCAAATGGTTTCCCATCGATCGCGAGACATACCTTGACCGCCTTTCAGTCAGGTTTGAGCCGGAAAACAAGCCTAATATGCTTTCTCCAATAGATATCTTTATAACAACCGCAGATCCAATCAAGGAACCACCTCTTGTTACAGCAAATACTGTTCTTTCAATTTTGGCACTAGATTATCCTGCAAACAAAATTTCATGTTACGTTTCTGATGACGGCGCTTCCATGCTCACCTTTGAAGCACTTCAAGAAACAGCTGAATTTGCGCAAAAATGGGTACCTTTCTGTAAACAATTCTCTACCGAGCCGCGTGCACCTGAGAAGTATTTCTCTGAGAAGATAGACTTTCTTAAGGATAAGCTTCAACCGACATACGTAAAAGAACGCCGTGCTATGAAGAGAGAATATGAAGAGTTTAAGGTGAGAATAAATGCGCTTGTGGCTAAATCTATGAGAGTTCCATCAGAAGGTTGGAGTATGAAAGATGAAACACCATGGCCAGGAAACAACACAAAAGATCATCCAAGTATGATACAAATACTTCTTGGTCACAATGGAGGAGACAATGAAGGAAATGAACTTCCATCTCTNGTCTACATTTCTAGAGAGAAAAGACCTGCATTTCAACATCACACAAAAGCCGGTGCAATGAACGCCTTGCTTCGTGTATCGGCAGTATTGAGCAATGCTCCTTTTGTGCTCAACTTGGACTGCAATCATTATGTGAATTACAGCAAAGTTGTGAGAGAAGCCATGTGTTTCTTTATGGACATTCAACTTGGGAATAGTATTGCTTTTGTTCAGTTTCCACTGAGATTTGATAGTCTTGATAGGAACGATCGTTATGCCAACAAAAACACTATTTTATTTGATATCAACTTGAGGTGTCTAGATGGAATTCAAGGACCTGTTTATATTGGATCAGGTTGTATATTCAGAAGGAAAGCTTTAAATGGCTTT

***MsCesA8***

ACTTCCTAGGCTAGTTTATGTTTCTAGAGAGAAAAGACCAGGATACCAACACCACAAGAAAGCAGGTGCTGAAAACGCACTGGTGAGGGTGTCTGCAGTTCTCACAAATGCTCCCTTCATTCTCAATCTTGATTGTGATCATTATGTTAACAACAGCAAGGCTGTTCGAGAAGCAATGTGTTTTCTCATGGATCCAGAAGTTGGTAGAGATGTTTGTTATGTACAATTCCCCCAAAGATTTGATGGTATTGACCGTAGTGATCGATATGCCAACCGCAATACAGTTTTCTTTGATGTAAACATGAGAGGACTTGATGGCATTCAAGGACCAATGTATGTGGGGACTGGTTGTGTTTTCAATCGGCAAGCACTTTATGGCTATAGCCCACCTTCTATGGTCAATTCACCAATGTCTTCATGCTGTTGCTGCCCCTCCACCAAAGAAGTGTCACGGGTTTCTAGAGATGGAAAAAGGGCAGAACTTGATGCTGCAATTTATAATCTCAGGGAGATTGATAATTATGATGAGAATGAGAGGTCAATGCTAATTTCACAAATGAGCTTTGAAAAAACTTTTGGCTTGTCTACTGTTTTCATTGAATCTGCATTAATGGAGAATGGAGGAGGGGTACCAGAATCTGCAGATCCTTCAATGCTGATCAAGGAGGCCATTCATGTAATTAGCTGTGGGTATGAAGAGAAGACTGAATGGGGAAAAGAGATTGGTTGGATTTATGGTTCAGTTACTGAGGATATCTT

AACAGGGTTCAAGATGCA

**Figure S1**
