# Supplementary material for: Alfalfa Cellulose Synthase Gene Expression under Abiotic Stress: A Hitchhiker’s Guide to RT-qPCR Normalization
Source: PLoS One. 2014 Aug 1;9(8):e103808. doi: 10.1371/journal.pone.0103808 (PMC4118957; doi:10.1371/journal.pone.0103808)
Supplement: Figure S2 — Alignment of alfalfa partial CesA sequences with M. truncatula CesAs. Alignment of the CesAs from alfalfa with the respective orthologs from M. truncatula. (DOC) [file pone.0103808.s002.doc]

MtCesA1 ATGGAAGCCTCTTCCGGCATGGTTGCTGGCTCCCACAATCGTAACGAGCTTGTTCGTATT 60

MsCesA1 ------------------------------------------------------------

MtCesA1 CGTCACGATTCTGCCGACAGCGGGCCGAAACCATTGAAGAATTTGAATGGACAAGTCTGT 120

MsCesA1 ------------------------------------------------------------

MtCesA1 CATATATGTGGTGAGGATGTTGGAACAACACCCACCGGTGATGTGTTTGTGGCTTGTAAT 180

MsCesA1 ------------------------------------------------------------

MtCesA1 GAATGTGGTTACCCTGTTTGTCGAGATTGTTATGAATATGAGCGAAAAGAAGGCAATAAA 240

MsCesA1 ------------------------------------------------------------

MtCesA1 TCTTGTCCTCAATGCAAAACTAGATACAAAAGGCTCAGAGGGAGTCCTAGAGTGGATGGA 300

MsCesA1 ------------------------------------------------------------

MtCesA1 GACGATGAAGAAGACGATGTTGATGATATCGAGAACGAGTTCAATTATCGACAAGGAAAT 360

MsCesA1 ------------------------------------------------------------

MtCesA1 AATAATAATAATAAGTCAAGGAGACAGTGGGATGATTCAGACAGATCAGCTTCATCTTCT 420

MsCesA1 ---AATAATAATAAGTCAAGGAGACAGTGGGATGATTCAGACAGATCAGCTTCATCTTCT 57

*********************************************************

MtCesA1 AGACGCGAATATCAGCAACCTCCTCTTCTCACCAATGGCCAAACTATGTCTGGCGAGATC 480

MsCesA1 AGACGCGAATATCAGCAACCTCCTCTTCTCACCAATGGCCAAACTATGTCTGGCGAGATN 117

***********************************************************

MtCesA1 CCTACACCTGATAATCAATCTGTCCGAACTACTTCTGGTCCTTTGGGCCCATCTGAGAAA 540

MsCesA1 CCTACACCTGATAATCAATCTGTCCGAACTACTTCTGGTCCTTTGGGCCCATCTGAGAAA 177

************************************************************

MtCesA1 GCTCACTCACTTCCCTATATCGATCCAAGGCAACCAGTTCCGGTGAGAATTGTGGATCCA 600

MsCesA1 GCTCACTCACTTCCCTATATTGATCCAAGGCAACCACTTCCGGTGAGAATTGTGGATCCA 237

******************** *************** ***********************

MtCesA1 TCAAAGGACTTAAACTCATATGGTTTGGGAAACGTTGACTGGAAGGAAAGGGTTGAAGGT 660

MsCesA1 TCAAAGGACTTAAACTCATATGGTTTGGGAAATGTTGACTGGAAGGAAAGGGTTGAAGGT 297

******************************** ***************************

MtCesA1 TGGAAGCTGAAGCACGAGAAAAATATGGTACAGATGACCGGTAGATATGCTGATGGGAAA 720

MsCesA1 TGGAAGCTGAAGCACGAGAAAAATATGGTACAGATGACTGGTAGATATGCTGATGGGAAA 357

************************************** *********************

MtCesA1 AGCGGAGGAGGAGATATTGAAGGGACTGGTTCTAATGGAGAAGAACTTCAAATGGTTGAT 780

MsCesA1 AGCGGAGGAGGAGATATTGAAGGGACTGGTTCTAATGGAGAAGAACTTCAAATGGTTGAT 417

************************************************************

MtCesA1 GATGCTCGACAACCTATGAGTCGGATTGTACCTATTTCTTCATCTCAGCTGACCCCTTAT 840

MsCesA1 GATGCTCGACAACCTATGAGTCGGATTGTACCCATTTCTTCATCTCAGCTGACCCCTTAT 477

******************************** ***************************

MtCesA1 CGTGTTGTCATCGTGTTCCGGCTGATAGTTCTTGGTTTCTTCTTGCAATATCGTGTAACT 900

MsCesA1 CGTGTTGTCATCGTGTTCCGGCTGATAGTTCTTGGTTTCTTCTTGCAATATCGTGTAACT 537

************************************************************

MtCesA1 CACCCGGTAAAAGATGCTTACCCACTGTGGTTGACTTCAGTTATCTGTGAGATTTGGTTT 960

MsCesA1 CACCCGGTAAAAGATGCTTACCCACTGTGGTTGACTTCAGTTATCTGTGAGATTTGGTTT 597

************************************************************

MtCesA1 GCATTTTCCTGGATTTTGGATCAGTTTCCAAAATGGTCTCCCATTAACCGTGAGACTTAT 1020

MsCesA1 GCATTTTCCTGGATTTTGGATCAGTTTCCAAAATGGTCTCCCATTAACCGTGAGACTTAT 657

************************************************************

MtCesA1 CTGGAGAGGCTTGCCATAAGATATGATCGTGATGGAGAGCCATCACAGTTGGCTCCTGTT 1080

MsCesA1 CTGGAGAGGCTTGCCATAAGATATGATCGTGATGGAGAACCATCACAGTTGGCTCCTGTC 717

**************************************.********************

MtCesA1 GACGTATTTGTCAGTACAGTGGACCCTCTCAAAGAGCCACCTATTGTAACTGCAAACACT 1140

MsCesA1 GACGTATTTGTCAGTACAGTGGACCCTCTCAAAGAGCCACCTATTGTAACTGCAAACACT 777

************************************************************

MtCesA1 GTTTTGTCTATACTTGCCGTTGATTACCCTGTCGACAAGGTTTCTTGCTATGTATCTGAT 1200

MsCesA1 GTTTTGTCTATACTCGCTGTTGACTACCCTGTCGACAAGGTTTCTTGCTATGTATCTGAT 837

************** ** ***** ************************************

MtCesA1 GATGGTTCAGCTATGTTGAGTTTTGAAGCCTTATCTGAAACAGCCGAGTTTGCAAAGATG 1260

MsCesA1 GATGGTTCAGCTATGTTGAGTTTTGAAGCCTTATCTGAAACAGCCGAGTTTGCAAAGATG 897

************************************************************

MtCesA1 TGGGTGCCCTTTTGCAAAAAACACAGTATTGAGCCAAGAGCACCTGAATTTTATTTTCTT 1320

MsCesA1 TGGGTGCCCTTTTGCAAAAAACACAGTATTGAGCCAAGAGCACCTGAATTTTATTTTCTT 957

************************************************************

MtCesA1 CAGAAGATTGATTACTTAAAGGACAAGGTTCAACCCTCTTTTGTTAAGGAGCGACGAGCA 1380

MsCesA1 CAGAAGATTGATTACTTAAAGGACAAGGTTCAACCCTCTTTTGTTAAGGAGCGACGAGCA 1017

************************************************************

MtCesA1 ATGAAGAGACAATATGAAGAATTCAAAGTAAGGATCAATGCCTATGTGGCCAAAGCTCAG 1440

MsCesA1 ATGAAGAGACAATATGAAGAATTCAAAGTAAGGATCAATGCCTATGTTGCTAAAGCTCAG 1077

*********************************************** ** *********

MtCesA1 AAGATGCCAGAGGAAGGTTGGACAATGCAGGATGGAACTCCTTGGCCTGGAAATAATCCC 1500

MsCesA1 AAGATGCCAGAGGAAGGTTGGACAATGCAGGATGGAACTCCTTGGCCTGGAAATAATCCC 1137

************************************************************

MtCesA1 AGGGATCATCCTGGAATGATTCAGGTGTTTTTAGGTCATAGTGGAGGGCTTGATACAGAT 1560

MsCesA1 AGGGATCATCCTGGAATGATTCAGGTGTTCTTAGGACATAGTGGAGGGCTTGATACAGAT 1197

***************************** *****:************************

MtCesA1 GGCAATGAACTTCCCAGGCTTGTTTATGTGTCTCGTGAAAAGCGACCAGGCTTCCAACAT 1620

MsCesA1 GGCAATGAACTTCCCAGGCTTGTTTATGTGTCTCGTGAAAAGCGACCAGGCTTCCAACAT 1257

************************************************************

MtCesA1 CACAAGAAGGCTGGAGCTATGAATGCTTTGATTCGAGTTTCTGCTGTCTTGACCAACGGT 1680

MsCesA1 CACAAGAAGGCTGGAGCTATGAATGCTTTGATTCGAGTTTCTGCTGTCTTGACCAACGGT 1317

************************************************************

MtCesA1 GCATATCTTTTGAATGTCGATTGTGATCATTATTTCAATAATAGCAAAGCTCTTAAGGAG 1740

MsCesA1 GCATATCTTTTGAATGTCGATTGTGATCATTATTTCAATAATAGCAAAGCTCTTAAGGAG 1377

************************************************************

MtCesA1 GCTATGTGTTTCATGATGGATCCTGCTTACGGAAAGAAGACATGCTATGTTCAATTTCCG 1800

MsCesA1 GCTATGTGTTTCATGATGGATCCTGCTTATGGAAAGAAGACATGCTATGTTCAATTTCCG 1437

***************************** ******************************

MtCesA1 CAGAGATTTGATGGCATTGATTTGCACGATCGATATGCCAATCGCAATATTGTCTTCTTC 1860

MsCesA1 CAGAGATTTGATGGCATTGATTTGCACGATCGATATGCCAATCGCAATATTGTCTTCTTC 1497

************************************************************

MtCesA1 GATATCAACTTGAAAGGCCAGGATGGTATTCAGGGCCCTGTCTATGTGGGAACTGGTTGC 1920

MsCesA1 GATATCAACTTGAAAGGTCAGGATGGTATTCAGGGCCCTGTCTATGTGGGAACTGGTTGC 1557

***************** ******************************************

MtCesA1 TGTTTCAATAGGCAAGCTTTGTATGGTTATGATCCTGTTTTGACTGAGGAAGATCTCGAA 1980

MsCesA1 TGTTTCAATAGGCAAGCTTTGTATGGTTATGATCCTGTTTTGACTGAGGAAGATCTCGAA 1617

************************************************************

MtCesA1 CCTAACATTATTGTTAAGAGTTGTTGGGGTTCTAGAAAGAAAGGAAAGGGTGGGAATAAG 2040

MsCesA1 CCTAACATTATTGTTAAGAGTTGTTGGGGTTCTAGAAAGAAAGGAAAGGGTGGGAATAAG 1677

************************************************************

MtCesA1 AAGTACGGTGACAAGAAGAGGGGAGTTAAAAGAACTGAATCCACCATTCCTATATTTAAT 2100

MsCesA1 AAGTACGGTGACAAGAAGAGGGGAGTTAAAAGAACTGAATCCACCATTCCCATATTTAAT 1737

************************************************** *********

MtCesA1 ATGGAGGATATAGAGGAGGGTGTCGAAGGTTATGATGATGAAAGGTCACTACTAATGTCT 2160

MsCesA1 ATGGAGGATATAGAGGAGGGTGTCGAAGGTTATGATGATGAAAGGTCACTTCTAATGTCT 1797

**************************************************:*********

MtCesA1 CAGAAGAGCTTGGAGAAGCGTTTCGGACAGTCTCCAGTTTTTATTGCTGCCACATTCATG 2220

MsCesA1 CAAAA------------------------------------------------------- 1802

**.**

MtCesA1 GAGCAAGGTGGCCTTCCACCTTCAACCAATTCTACAACTCTTCTTAAGGAAGCAATCCAT 2280

MsCesA1 ------------------------------------------------------------

MtCesA1 GTTATCAGCTGTGGTTATGAAGACAAGACAGAATGGGGCAAAGAGATTGGATGGATCTAT 2340

MsCesA1 ------------------------------------------------------------

MtCesA1 GGTTCTGTGACTGAAGATATCCTGACTGGGTTTAAGATGCATGCACGTGGTTGGATTTCC 2400

MsCesA1 ------------------------------------------------------------

MtCesA1 GTCTATTGCATGCCACCTCGTCCAGCATTTAAGGGTTCTGCTCCCATCAATCTTTCAGAC 2460

MsCesA1 ------------------------------------------------------------

MtCesA1 CGTCTCAATCAGGTGCTTCGGTGGGCCTTGGGTTCAATAGAGATCTTCCTTAGCAGGCAT 2520

MsCesA1 ------------------------------------------------------------

MtCesA1 TGTCCTTTATGGTATGGCTACAATGGAAGGATGAGGCCTCTCATGAGGCTGGCTTATATC 2580

MsCesA1 ------------------------------------------------------------

MtCesA1 AACACAATTATCTACCCCTTTACCTCAATCCCATTGCTTGCGTACTGTGTGCTTCCTGCA 2640

MsCesA1 ------------------------------------------------------------

MtCesA1 TTTTGTCTTCTCACAAACAAATTTATTATTCCCGAGATAAGTAACTTTGCAAGCATGTGG 2700

MsCesA1 ------------------------------------------------------------

MtCesA1 TTTATTCTCCTCTTTACCTCCATTTTCACTACTTCAATTCTGGAGCTTAGGTGGAGTGGT 2760

MsCesA1 ------------------------------------------------------------

MtCesA1 GTTGGTATAGAAGACTGGTGGAGAAACGAGCAGTTTTGGGTTATCGGTGGAACATCTGCA 2820

MsCesA1 ------------------------------------------------------------

MtCesA1 CATCTTTTCGCCGTGTTCCAAGGGCTTCTCAAAGTGCTTGCAGGGATTGACACAAATTTT 2880

MsCesA1 ------------------------------------------------------------

MtCesA1 ACCGTTACATCAAAGGCAAATGACGAAGACGGGGACTTTGCCGAGCTATACGTGTTCAAA 2940

MsCesA1 ------------------------------------------------------------

MtCesA1 TGGACATCACTTCTTATCCCTCCTACAACAGTACTTATTGTGAATTTGATAGGTATTGTG 3000

MsCesA1 ------------------------------------------------------------

MtCesA1 GCTGGTGTGTCTTTTGCCATAAACAGTGGTTACCAATCTTGGGGTCCACTATTTGGAAAG 3060

MsCesA1 ------------------------------------------------------------

MtCesA1 CTCTTCTTTGCTATATGGGTCATTGCTCATTTATACCCTTTCTTGAAGGGTCTTTTGGGG 3120

MsCesA1 ------------------------------------------------------------

MtCesA1 AAATCGAATCGCACACCAACTATTGTTATTGTCTGGGCCGTTCTCCTTGCTTCTATATTC 3180

MsCesA1 ------------------------------------------------------------

MtCesA1 TCTTTGTTGTGGGTGAGGATTGACCCCTTCATCTCTGATCCCAACAAATCATCATCCAAT 3240

MsCesA1 ------------------------------------------------------------

MtCesA1 AGTCAGTGTGGTATCAACTGCTAG 3264

MsCesA1 ------------------------

MtCesA6-B ATGCATACCGGTGGTAGACTCATTGCTGGTTCTCACAACAGGAACGAGTTTGTTCTCATC 60

MsCesA6-B ------------------------------------------------------------

MtCesA6-B AATGCTGAAGAAAATGGAAGGATTAAGTCGGTTCGCGAACTGAGTGGACAGATATGTATG 120

MsCesA6-B ------------------------------------------------------------

MtCesA6-B ATCTGTGGAGATGAGATTGAGGTTACTGTGGACGGGGAGCCCTTTGTTGCTTGCAATGAG 180

MsCesA6-B ------------------------------------------------------------

MtCesA6-B TGTGCTTTCCCTGTTTGCCGGCCTTGCTACGAGTACGAGCGAAAAGAAGGGAATCAGGCC 240

MsCesA6-B ------------------------------------------------------------

MtCesA6-B TGCCCTCAGTGCAAGACTAGATACAAACGCCTCAAAGGTAGTCCAAGGGTCGAAGGTGAT 300

MsCesA6-B ------------------------------------------------------------

MtCesA6-B GAAGAAGAGGATGGCGATGACGATCTAGACAATGAGTTTGATTATGATCTTGATGATATG 360

MsCesA6-B ------------------------------------------------------------

MtCesA6-B GGCCAGCAAGCACATTCAGATTCATTGTTTTCCGGCCGCCTTAACACCGGTCGTGGTTCT 420

MsCesA6-B ------------------------------------------------------------

MtCesA6-B AATACCAATATATCTGGTGCAAATTCGGAACATGGATCACCTCCTCTCAATCCTGAAATC 480

MsCesA6-B ------------------------------------------------------------

MtCesA6-B CCACTCCTGACTTATGGGGAGGAGGATCCTGAGATATCTTCTGATAGACATGCTCTAATT 540

MsCesA6-B ------------------------------------------------------------

MtCesA6-B GTTCCACCATATATGAATCATGGAAACCGGGTTCATCCCATGCCTTATACTGATCCATCG 600

MsCesA6-B ------------------------------------------------------------

MtCesA6-B ATTCCATTGCAACCAAGACCGATGGTTCCGAAGAAAGACATTGCGGTATATGGATATGGA 660

MsCesA6-B ------------------------------------------------------------

MtCesA6-B AGTGTGGCTTGGAAAGACCGAATGGAGGAATGGAAGAAAAGGCAAAGTGACAAACTTCAG 720

MsCesA6-B ------------------------------------------------------------

MtCesA6-B GTGGTGAAGCATGAAGGTGATAACAATGATGGAAGTGGAAGTTTCGGAGATGACTTCGAT 780

MsCesA6-B ------------------------------------------------------------

MtCesA6-B GACCCTGATCTGCCCATGATGGACGAAGGAAGGCAGCCACTTTCTCGGAAACTACCTATC 840

MsCesA6-B ------------------------------------------------------------

MtCesA6-B CCTTCAAGCAAGATAAATCCATACAGGATTATCATAGTTCTTCGTCTTGTTATTCTTGGG 900

MsCesA6-B ------------------------------------------------------------

MtCesA6-B CTTTTTTTCCATTATAGAATTCTCCATCCAGTTAATGATGCATATGGCTTGTGGCTGACA 960

MsCesA6-B ------------------------------------------------------------

MtCesA6-B TCAGTCATATGTGAAATATGGTTTGCTGTATCTTGGATAATGGATCAGTTTCCAAAATGG 1020

MsCesA6-B ------------------------------------------------------------

MtCesA6-B TACCCAATAACGCGAGAAACGTACCTTGACCGTCTTTCACTGAGGTATGAGAAAGAAGGG 1080

MsCesA6-B ------------------------------------------------------------

MtCesA6-B AAACCCTCCCAGTTGGCAAGTGTTGATGTCTTTGTCAGTACTGTTGATCCTATGAAAGAA 1140

MsCesA6-B ------------------------------------------------------------

MtCesA6-B CCTCCACTGATCACAGCAAACACTGTTCTATCTATCCTTGCTGTTGACTATCCAGTTGAT 1200

MsCesA6-B ------------------------------------------------------------

MtCesA6-B AAAGTTGCATGCTATGTTTCGGATGATGGTGCTGCTATGCTAACTTTTGAAGCACTTTCT 1260

MsCesA6-B ------------------------------------------------------------

MtCesA6-B GAGACATCTGAATTTGCTAGGAAGTGGGTTCCGTTTTGCAAGAAATATAATATTGAGCCC 1320

MsCesA6-B ------------------------------------------------------------

MtCesA6-B CGTGCCCCAGAATGGTATTTCGGTCAGAAGATGGACTATCTGAAAAATAAAGTGCATCCC 1380

MsCesA6-B ------------------------------------------------------------

MtCesA6-B GCGTTTGTTAGGGAAAGAAGAGCAATGAAGAGAGATTATGAAGAATTTAAGGTGAGGATT 1440

MsCesA6-B ------------------------------------------------------------

MtCesA6-B AATAGTTTGGTGGCAACGGCACAGAAGGTTCCCGAGGATGGATGGACTATGCAGGATGGG 1500

MsCesA6-B ------------------------------------------------------------

MtCesA6-B ACTCCGTGGCCTGGAAATGATGTGAGGGATCATCCTGGCATGATTCAGGTCTTCCTTGGT 1560

MsCesA6-B ------------------------------------------------------------

MtCesA6-B CATGATGGTGTTCGTGATGTTGAGGGAAATGAGTTACCTCGCTTAGTTTATGTTTCTAGA 1620

MsCesA6-B ------------------------------------------------------------

MtCesA6-B GAAAAAAGGCCTGGGTTTGATCACCACAAAAAGGCTGGAGCCATGAATTCTCTGGTACGA 1680

MsCesA6-B ------------------------------------------------------------

MtCesA6-B GCGGCGGCAATTATCACAAACGCACCTTATATTCTGAATGTTGACTGTGATCATTACATC 1740

MsCesA6-B ------------------------------------------------------------

MtCesA6-B AATAATAGCAAGGCTCTTAGAGAGGCCATGTGTTTTATGATGGACCCTCAACTTGGCAAG 1800

MsCesA6-B ------------------------------------------------------------

MtCesA6-B AAGATCTGCTATGTACAATTTCCACAAAGATTTGATGGAATTGATAGACATGACAGATAC 1860

MsCesA6-B ------------------------------------------------------------

MtCesA6-B TCCAACAGAAATGTTGTATTTTTTGATATAAACATGAAAGGATTGGATGGATTACAAGGT 1920

MsCesA6-B ------------------------------------------------------------

MtCesA6-B CCAATTTATGTTGGAACTGGATGTGTTTTCAGAAGGTATGCACTCTACGGATATGATGCA 1980

MsCesA6-B ------------------------------------------------------------

MtCesA6-B CCTGTCAAGAAGAAGCCGCCAAGCAAAACCTGCAACTGTTTGCCAAAATGGTGCTGCTGG 2040

MsCesA6-B ------------------------------------------------------------

MtCesA6-B TGCTGTGGCTCTAGAAAGAAAAAGAATCTCAATAATAAGAAAGATAAAAAGAAGAAGGTG 2100

MsCesA6-B ------------------------------------------------------------

MtCesA6-B AAGCATAGTGAAGCATCAAAGCAAATTCACGCACTTGAAAATATTGAGGCAGGGAATGAA 2160

MsCesA6-B ------------------------------------------------------------

MtCesA6-B GGAGCCATCGTCGAGAAATCATCCAATTTGACTCAATTGAAAATGGAGAAGAGGTTTGGA 2220

MsCesA6-B ------------------------------------------------------------

MtCesA6-B CAGTCTCCAGTGTTTGTTGCCTCTACACTTCTGGATAATGGTGGAATTCCACCTGGAGTG 2280

MsCesA6-B ------------------------------------------------------------

MtCesA6-B AGTCCTGCATCGCTGTTAAAAGAAGCCATTCAAGTTATCAGTTGTGGTTATGAAGACAAA 2340

MsCesA6-B ------------------------------------------------------------

MtCesA6-B ACCGAATGGGGAAAAGAGGTTGGATGGATCTATGGTTCTGTGACTGAGGATATCTTGACT 2400

MsCesA6-B ------------------------------------------------------------

MtCesA6-B GGTTTCAAAATGCATTGCCATGGTTGGCGGTCTGTGTATTGTATCCCTAAGAGGCCTGCA 2460

MsCesA6-B ------------------------------------------------------------

MtCesA6-B TTCAAGGGTTCAGCACCCATCAATCTTTCAGATCGTCTACACCAAGTTCTTCGTTGGGCT 2520

MsCesA6-B ------------------------------------------------------------

MtCesA6-B CTTGGTTCTGTTGAGATCTTTTTCAGCAAACATTGTCCAATTTGGTACGGCTACGGTGGT 2580

MsCesA6-B ------------------------------------------------------------

MtCesA6-B GGGTTGAAGTTGTTGGAACGTTTTTCCTACATAAATTCAGTCGTGTATCCTTGGACTTCC 2640

MsCesA6-B ------------------------------------------------------------

MtCesA6-B CTCCCATTGATTGTCTACTGTACTCTACCGGCCATATGTCTTCTGACCGGAAAATTTATT 2700

MsCesA6-B ------------------------------------------------------------

MtCesA6-B GTTCCTGAGATAAGCAACTATGCAAGTCTTGTGTTCATGGCCCTCTTCATATCGATCGCA 2760

MsCesA6-B -------------------TTGCAAGTCTTGTGTTCATGGCCCTCTTCATATCCATCGCA 41

:********************************* ******

MtCesA6-B GCAACTGGTATCCTTGAGATGCAATGGGGTGGTGTTGGAATCGATGATTGGTGGAGGAAT 2820

MsCesA6-B GCAACCGGTATCCTTGAGATGCAATGGGGTGGTGTTGGAATAGATGATTGGTGGAGGAAT 101

***** ***********************************.******************

MtCesA6-B GAACAGTTTTGGGTGATTGGAGGTGCTTCATCACATCTTTTTGCCCTTTTCCAAGGTTTG 2880

MsCesA6-B GAACAGTTTTGGGTGATTGGAGGTGCTTCATCACATTTTTTTGCCCTTTTCCAAGGTTTG 161

************************************ ***********************

MtCesA6-B CTCAAGGTTTTAGCTGGTGTCGACACAAACTTCACTGTTACATCAAAAGCAGCCGACGAT 2940

MsCesA6-B CTCAAGGTTTTAGCTGGTGTCGACACAAACTTCACTGTTACATCAAAAGCAGCCGACGAT 221

************************************************************

MtCesA6-B GGAGAATTCTCGGAGCTCTATGTATTCAAATGGACTTCACTATTAATCCCTCCAATGACG 3000

MsCesA6-B GGAGAATTCTCGGAGCTCTACGTATTCAAATGGACTTCGCTATTAATCCCTCCAATGACG 281

******************** *****************.*********************

MtCesA6-B CTATTAATCATGAATATTGTGGGCGTGATTGTTGGTGTCTCCGATGCGATCAATAATGGT 3060

MsCesA6-B CTATTAATCATGAATATTGTGGGCGTGATTGTTGGTGTCTCCGATGCGATCAATAATGGT 341

************************************************************

MtCesA6-B TATGACTCATGGGGACCTCTGTTCGGTAGATTATTCTTTGCCCTTTGGGTCATCATACAT 3120

MsCesA6-B TATGACTCATGGGGACCTCTGTTCGGTAGATTATTCTTTGCCCTTTGGGTTATCATACAT 401

************************************************** *********

MtCesA6-B CTTTATCCATTCCTCAAGGGTTTGCTTGGGAAACAGGATAGGATGCCAACCATTGTTTTG 3180

MsCesA6-B CTTTATCCATTCCTCAAGGGTTTGCTTGGGAAACAGGATAGGATGCCAACCATTGTTTTG 461

************************************************************

MtCesA6-B GTTTGGTCAATCTTGCTAGCTTCCATCTTGACTCTCTTGTGGGTTAGAGTTAACCCTTTT 3240

MsCesA6-B GTTTGGTCAATCTTGCTAGCTTCCATCTTGACTCTCTTGTGGGTTAGAGTTAACCCTTTT 521

************************************************************

MtCesA6-B GTATCAAGAGATGGTCCTGTCTTAGAAATTTGTGGATTGAATTGTGAGGATACATGA 3297

MsCesA6-B GT------------------------------------------------------- 523

**

MtCesA6-C ATGGACACTAATGGAAGATTAGTTGCAGGATCACATAACAGGAATGAGTTTGTTCTTATC 60

MsCesA6-C ATGGACACTAATGGAAGATTAGTTGCAGGATCACATAACAGGAATGAGTTTGTTCTTATC 60

************************************************************

MtCesA6-C AATGCTGATGACACTGCAAGAGTGAGTGTGAATGCTGTGACAGAATTGAGTGGACAAATT 120

MsCesA6-C AATGCTGATGACACTGCAAGAGT------GAATGCTGTGACAGAATTGAGTGGACAAATT 114

*********************** *******************************

MtCesA6-C TGCCAGATCTGTGGGGATGAGATAGAGTTTACAGTGGATGATGAACCTTTTGTTGCTTGC 180

MsCesA6-C TGCCAGATCTGCGGGGATGAGATAGAGCTTACAGTGGATGATGAACCTTTTGTTGCTTGC 174

*********** *************** ********************************

MtCesA6-C AATGAATGTGCATTCCCTGTGTGTAGACCTTGCTATGAGTATGAAAGACGAGAAGGGAAC 240

MsCesA6-C AATGAATGTGCATTCCCTGTGTGTAGACCCTGCTATGAGTATGAAAGAAGAGAAGGGAAT 234

***************************** ******************.**********

MtCesA6-C CAAGCTTGTCCTCACTGCAAAACTAAATACAAACGCATAAAGGGTAAGCCTTCAACCGAC 300

MsCesA6-C CAAGCTTGTCCTCACTGCAAAACTAAATACAAACGCATAAAGG----------------- 277

*******************************************

MtCesA6-C TTTTTTTTTCTTTACTTTGTTATATATAAGTGGAAGAAAAAGTTTTGGCTAACAAAGCAG 360

MsCesA6-C ------------------------------------------------------------

MtCesA6-C AGCCAATATTTGGGTATGGCACTCTGCAGTTGGAGACGTAGTCCAAGAGTTGAGGGTGAC 420

MsCesA6-C -------------------------------------GTAGTCCCAGAGTTGAGGGTGAT 300

*******.**************

MtCesA6-C GAAGAAGAGGATGGGATTGATGATTTGGAAAATGAGTTTGATATTGGAAGCAATATCAAA 480

MsCesA6-C GAAGAAGAGGACGGTATTGATGATTTGGAAAATGAGTTTGACATTGGAAGCAATATCAAA 360

*********** ** ************************** ******************

MtCesA6-C CATGACCCTCATCACATTACTGAGGCCATGTTCTTTTCTCACCTCAACAATATCGGTCGG 540

MsCesA6-C CATGACTCTCATCACATTACCGATGCTATGTTCTTCGCTCGCCTCAACAATATTGGCCAG 420

****** ************* ** ** ******** ***.************ ** *.*

MtCesA6-C AGTTCACAAATGAATGCTTCAAGAATCACCACGCCATCAGAGTTTGACACGGCTTCTGTG 600

MsCesA6-C AGTTCACTAATGAATGCTTCAGGAATCACTACACCATCGGAGTTTGATGCAGCTTCTATG 480

*******:*************.******* **.*****.******** .*.******.**

MtCesA6-C GCTGCTGATATCTCTCTCCTGACATATGATCATGAGGTTGAGGATCCTGGGATTTCTTCT 660

MsCesA6-C GCTGCTGATATACCCTTCCTGACATACGATCATGAG------GATCTTGGAATTTCTTCT 534

***********. * ********** ********* **** ***.*********

MtCesA6-C GATAAACATGCTCTCATTATCCCTCCATACAAGCTCCGCGGGAAACGGGTTCATCCTATG 720

MsCesA6-C GATAAACATGCTCTGATTATCCCTCCGTGCAAGCCCCACGGGAAACGGGTTCATCCTATG 594

************** ***********.*.***** **.**********************

MtCesA6-C CCTTTTCCTGATTCATTTGTGCCGGTTCTACCAAGACCCATGGATCCTAATAAAGATTTG 780

MsCesA6-C CCTTTTCCTGATTCATTTGTGCCGGTTCTACCAAGACCTATGGATCCTAATAAAGATTTG 654

************************************** *********************

MtCesA6-C GCTGTTTATGGCTATGGAAGTGTTGCATGGAAAGAAAGAGTGGAGGAATGGAAGAAAAAG 840

MsCesA6-C GCTGTTTATGGCTATGGAAGTGTTGCATGGAAAGAAAGAGTGGAGGAATGGAAGAAAAGG 714

**********************************************************.*

MtCesA6-C CAGAATGAAAAATTAGAGGTGGTTAAGCACGAAGGTGACAACAATGTTGATGAGTTTAAT 900

MsCesA6-C CAGAATGAAAAATTAGAGGTGGTTAAGCACGGGGTTGATAACAATGTTGATGAGTTTAAT 774

*******************************..* *** *********************

MtCesA6-C GATCCCGATTTGCCAAAAATGGATGAAGGAAGGCAACCGCTATGGAGGAAGTTACCAATC 960

MsCesA6-C GATCCCGATTTGCCAAAAATGGATGAA--------------------------------- 801

***************************

MtCesA6-C AGTCCAAGCAAGATAAATCCATATCGAATCATTATAGTACTCCGGATCGCTGTTCTTGCT 1020

MsCesA6-C ------------------------------------------------------------

MtCesA6-C CTCTTTTTTCATTACAGAATTCTCCATCCGGTCAACGATGCATATGCATTGTGGTTGACA 1080

MsCesA6-C ------------------------------------------------------------

MtCesA6-C TCAGTAATATGTGAAATTTGGTTTGCTGTATCATGGATTTTGGATCAGTTTCCAAAATGG 1140

MsCesA6-C ------------------------------------------------------------

MtCesA6-C TCTCCAGTTGAGCGAGAAACATACCTTGATCGTTTATCACTGAGGTACGAGAAAGAAGGA 1200

MsCesA6-C ------------------------------------------------------------

MtCesA6-C AAGCCGTGTGAATTATCCGATATTGACATATTTGTTAGTACTGTGGATCCTATGAAAGAG 1260

MsCesA6-C ------------------------------------------------------------

MtCesA6-C CCTCCTCTTATAACTGCAAACACAGTTCTGTCCATCCTAGCAGTAGATTATCCGGTGGAA 1320

MsCesA6-C ------------------------------------------------------------

MtCesA6-C AAAGTGGCGTGCTATGTCTCAGACGACGGTGCAGCTATGCTAACATTTGAAGCCCTTTCA 1380

MsCesA6-C ------------------------------------------------------------

MtCesA6-C GAAACTTCAGAGTTTGCAAGAAAATGGGTTCCATTCTGCAAGAAGTTCAGCATTGAACCG 1440

MsCesA6-C ------------------------------------------------------------

MtCesA6-C CGGGCTCCTGAGTGGTATTTTGCTCAGAAGGTTGACTATCTAAAAGACAAAGTGGACGCG 1500

MsCesA6-C ------------------------------------------------------------

MtCesA6-C GCTTTCATCAAGGAGCGTCGTGCTATTAAGAGGGACTATGAAGAGTTAAAAGTGAGGATT 1560

MsCesA6-C ------------------------------------------------------------

MtCesA6-C AATGCATTGGTTGCAATGGCGCAAAAGGTTCCTGAGGATGGATGGACAATGCAAGATGGG 1620

MsCesA6-C ------------------------------------------------------------

MtCesA6-C ACACCTTGGCCTGGAAACAATGTCAACGATCATCCTGGAATGATTCAGGTTTTCCTTGGA 1680

MsCesA6-C ------------------------------------------------------------

MtCesA6-C CAAAATGGTGTTCGCGATATTGATGGGAACGAGTTACCTCGTCTTGTTTATGTGTCTCGT 1740

MsCesA6-C ------------------------------------------------------------

MtCesA6-C GAAAAAAGACCTGGATTCGAACACCACAAAAAAGCTGGTGCTATGAATGCCTTGGTGCGA 1800

MsCesA6-C ------------------------------------------------------------

MtCesA6-C GTCTCGGCAGTCATATCAAATGCTCCTTACATGCTAAATGTTGATTGTGACCACTACATA 1860

MsCesA6-C ------------------------------------------------------------

MtCesA6-C AACAACAGTAAGGCCCTTCGTGAAGCCATGTGTTTCATGATGGATCCTACATCAGGGAAA 1920

MsCesA6-C ------------------------------------------------------------

MtCesA6-C AAAATATGCTATGTGCAATTTCCTCAAAGATTTGATGGAATTGATCGTCATGATAGATAC 1980

MsCesA6-C ------------------------------------------------------------

MtCesA6-C TCAAATCGTAATGTTGTATTCTTTGATATCAATATGAAAGGCTTAGATGGCATCCAAGGA 2040

MsCesA6-C ------------------------------------------------------------

MtCesA6-C CCAATATATGTGGGAACTGGGTGTGTCTTCAGGAGGCAAGCACTCTATGGATATGATGCT 2100

MsCesA6-C ------------------------------------------------------------

MtCesA6-C CCTGCCAAGAAGAAACCACCAGGGAAGACATGTAATTGTTGGCCAAAATGGTGCTTCATG 2160

MsCesA6-C ------------------------------------------------------------

MtCesA6-C TGTTGTGGATCAAGAAAGAAGAATAGGAAAGTGAACTCCGGTCCAAGAAAGAAGATAAGG 2220

MsCesA6-C ------------------------------------------------------------

MtCesA6-C GATAAGGACGTCGCAAAACAAATACATGCACTAAAAAATATAGAAGAGGGAATTGAAGGA 2280

MsCesA6-C ------------------------------------------------------------

MtCesA6-C ATTGACAAGAAAAAGTCACCGCTAATATCGCAACTAAAATTTGAGAAATTTTTTGGACAA 2340

MsCesA6-C ------------------------------------------------------------

MtCesA6-C TCGTCTGTTTTCATAGCTTCAACGCTTATGGAAGATGGAGGCATTCTAAAAGCAGCAACT 2400

MsCesA6-C ------------------------------------------------------------

MtCesA6-C TCTGCATCACTCTTGAAAGAAGCCATCCATGTTATCAGTTGCGGTTACGAAGACAAGACT 2460

MsCesA6-C ------------------------------------------------------------

MtCesA6-C GAGTGGGGAAAAGAGGTTGGATGGATATATGGTTCCGTCACAGAAGATATTTTAACAGGT 2520

MsCesA6-C ------------------------------------------------------------

MtCesA6-C TTTAAGATGCATAGTCATGGTTGGCGATCGGTATACTGCATGCCTAAAAGGCCTGCTTTT 2580

MsCesA6-C ------------------------------------------------------------

MtCesA6-C AAAGGTTCAGCTCCTATAAACCTCTCGGATCGTCTGCATCAAGTTCTTCGGTGGGCGCTT 2640

MsCesA6-C ------------------------------------------------------------

MtCesA6-C GGATCTGTTGAGATTCTGTTAAGTAGGCACTGTCCTATTTGGTATGGATATGGCTGTGGC 2700

MsCesA6-C ------------------------------------------------------------

MtCesA6-C TTGAAATGGTTGGAACGTTTGTCTTACATAAACTCGGTTGTTTATCCTTTGACGTCAATT 2760

MsCesA6-C ------------------------------------------------------------

MtCesA6-C CCCTTGATTGTCTACTGCACCTTGCCAGCTGTGTGTCTTCTAACTGGGAAGTTCATAGTT 2820

MsCesA6-C ------------------------------------------------------------

MtCesA6-C CCTGAGATTAGCAACTATGCTAGTATCATTTTCATTGCACTTTTCATCTCTATAGCTGCA 2880

MsCesA6-C ------------------------------------------------------------

MtCesA6-C ACCGGCATCCTAGAAATGCAATGGGGAGGTGTTGGTATACAAGACTGGTGGAGGAATGAA 2940

MsCesA6-C ------------------------------------------------------------

MtCesA6-C CAATTCTGGGTCATCGGGGGCGCCTCCTCACACCTTTTTGCTCTCTTTCAAGGTTTGCTT 3000

MsCesA6-C ------------------------------------------------------------

MtCesA6-C AAGGTTCTAGCAGGGGTTAACACAAACTTCACTGTCACATCCAAAGCCGCCGATGATGGA 3060

MsCesA6-C ------------------------------------------------------------

MtCesA6-C GACTTTGCTGACCTCTACATCTTCAAGTGGACATCATTGTTAATCCCTCCTTTAACCTTG 3120

MsCesA6-C ------------------------------------------------------------

MtCesA6-C CTTATCCTAAACATAATTGGAGTTATTGTTGGTGTTTCAGATGCAATAAACAATGGCTAT 3180

MsCesA6-C ------------------------------------------------------------

MtCesA6-C GATTCATGGGGTCCATTATTTGGCAAGTTATTTTTTGCTCTATGGGTTATTTTACATCTT 3240

MsCesA6-C ------------------------------------------------------------

MtCesA6-C TATCCATTTCTTAAGGGTGTTATGGGAAAACAAGAAGGTGTTCCTACCATCATTTTGGTT 3300

MsCesA6-C ------------------------------------------------------------

MtCesA6-C TGGGCTATTCTTCTGGCTTCAATCTTTTCACTTCTATGGGTTAGGATCAACCCCTTTGTG 3360

MsCesA6-C ------------------------------------------------------------

MtCesA6-C TCCAAAAATGACATTGTGTTGGAACTTTGTGGGTTGAATTGTGATTGA 3408

MsCesA6-C ------------------------------------------------

MtCesA6-F ATGGAAACCAATTTTGGGTTAGTTGCAGGCTCTCACAACAAGAATGAATTCATTATCATA 60

MsCesA6-F ------------------------------------------------------------

MtCesA6-F CGTCAAGATGGTGACTATGCTAGAACAGATTTGCAAGAGTTAGATGGTGATACATGTCAA 120

MsCesA6-F ------------------------------------------------------------

MtCesA6-F CTATGTGGAGAAGACATAGGGGTTAATGCAGATGGTGACCCCTTTGTGGCTTGCAATGAA 180

MsCesA6-F ------------------------------------------------------------

MtCesA6-F TGTGCATTTCCTGTTTGTAGAAATTGTTATGAATATGAAAGAAGGGAGGGAAACCAAGTT 240

MsCesA6-F ------------------------------------------------------------

MtCesA6-F TGTCCTCAATGCAAAACCAAATTTAAGCGTCTTAAGGGGTGTGCTAGAGTTGAAGGAGAT 300

MsCesA6-F ------------------------------------------------------------

MtCesA6-F GAAGAGGAAGATGACATTGATGATTTAGAGAATGAATTCGATGAAGGGCGAAATGAACAA 360

MsCesA6-F ------------------------------------------------------------

MtCesA6-F GATATGCAAATCCCCATGTCTCCTGAGGGTGAGGAACTGTCGTCTGAAGAGCATCATGCT 420

MsCesA6-F ------------------------------------------------------------

MtCesA6-F ATAGTTCCTTTAATAAATTCAACAATTATGAGAAAAGAGATAACTTTATTGCAAGCAAGA 480

MsCesA6-F ------------------------------------------------------------

MtCesA6-F CCAATGGATCCTTCCAAGGACCTAGCTGCTTATGGCTATGGAAGTGTTGCTTGGAAGGAT 540

MsCesA6-F ------------------------------------------------------------

MtCesA6-F AGGATGGAGCTATGGAAGCAAAGACAGAACCAACTTGGTAATATGAGAAAAGATGATAAT 600

MsCesA6-F ------------------------------------------------------------

MtCesA6-F GAAGACTTGGATAAGAGTGTGGATGATGACAATGAATTTCCTTTAATGGATGAATCAAGG 660

MsCesA6-F ------------------------------------------------------------

MtCesA6-F CAACCATTGTCAAGGAAGTTGCCAATTCCATCAAGTCAAATCAATCCTTACCGTATGATC 720

MsCesA6-F -------------------------------------------ATCCGTACCGTATGATC 17

**** ************

MtCesA6-F ATAATAATAAGACTGATTGTTCTTGGATTCTTCTTCCAATATAGAATTATGCATCCAGTA 780

MsCesA6-F ATAATAATAAGACTGATTGTTCTTGGATTCTTCTTCCAATATAGAATTATGAATCCAGTA 77

***************************************************.********

MtCesA6-F GACAATGCTTATGCTTTGTGGCTTGTGTCAGTAATATGTGAGATTTGGTTCACTCTTTCA 840

MsCesA6-F GACAATGCATATGCTTTGTGGCTTGTGTCAGTAATATGTGAGATTTGGTTCACTCTTTCA 137

********:***************************************************

MtCesA6-F TGGATTCTTGATCAGTTCTCAAAGTGGTTTCCTGTCATGAGGGAAACCTATCTTGACAGA 900

MsCesA6-F TGGATTCTTGATCAGTTCTCAAAGTGGTTTCCTGTCATGAGGGAAACCTATCTTGACAGG 197

***********************************************************.

MtCesA6-F CTTTCCTTAAGG------CAAGAAGGTCAACCATCACAACTTTCACCAATTGATATATTT 954

MsCesA6-F CTTTCCTTAAGGTATGAAAAGGAAGGTCAACCATCACAACTTTCACCAATTGATATATTT 257

************ .*.***************************************

MtCesA6-F GTGACTACAAATGATCCATTAAAAGAATCTCCTCTAGTGACAGCAAACACAGTTCTATCA 1014

MsCesA6-F GTGACTACAAATGATCCATTAAAAGAGTCTCCTCTAGTGACAGCAAACACAGTTCTATCA 317

**************************.*********************************

MtCesA6-F ATTCTAGCAATAGATTACCCTGCTGAAAAGGTGTCATGTTATGTTTCTGATGATGGAGCA 1074

MsCesA6-F ATTCTAGCAATAGATTACCCTGCTGAAAAGGTGTCATGTTATGTTTCTGATGATGGAGCA 377

************************************************************

MtCesA6-F GCAATGTTAACATTTGAGGCATTATCTGAAACTTCTGAATTTGCAAGGAAATGGGTTCCT 1134

MsCesA6-F GCAATGTTAACATTTGAGGCTTTATCTGAAACTTCTGAATTTGCAAGGAAATGGGTTCCT 437

********************:***************************************

MtCesA6-F TTTTGTAAGAAGTTCAACATTGAACCTAGAGCTCCAGAATGGTATTTTCATGAGAAAATA 1194

MsCesA6-F TTTTGTAAGAAGTTCAACATTGAACCTAGAGCTCCAGAATGGTATTTTGCTGAGAAAATA 497

************************************************ .**********

MtCesA6-F AATTATTTAAAAGATAAGGTGCATTCATCATTTGTCAAAGAGAGAAGAGCAATGAAGAGA 1254

MsCesA6-F AATTATTTAAAAGATAAGGTTCATTCATCATTTGTGAAAGAGAGAAGAGCAATGAAG--- 554

******************** ************** *********************

MtCesA6-F GAATATGAAGAATTTAAAGTTAGAATAAATTCTCTAGTTGCAAAGGCTAAGAAGGTTCCA 1314

MsCesA6-F -------------------------------------TTGCAAAGGCTAAGAAGGTTCCA 577

***********************

MtCesA6-F GAAGAAGGGTGGACAATGCAAGATGGAATGTTATGGCCTGGAAATAATATTCGTGATCAT 1374

MsCesA6-F GAAGAAGGGTGGACAATGCAAGATGGAATGTTGTGGCCTGGAAATAATATTCGTGACCAT 637

********************************.*********************** ***

MtCesA6-F CCAGGAATGATACAGGTTTTTTTAGGTGAAAATGGGGGATGTGACATGGATGGAAATGAA 1434

MsCesA6-F CCAGGAATG--------------------------------------------------- 646

*********

MtCesA6-F TTGCCACGTTTGGTGTATGTTTCTAGAGAAAAGAGGCCTAACTTCAATCACCAAAAAAAA 1494

MsCesA6-F ------------------------------------------------------------

MtCesA6-F GCAGGAGCTCTAAATGCACTGGTTAGAGTGTCTTCTGTGCTTTCCAATGCACCTTTTGTG 1554

MsCesA6-F ------------------------------------------------------------

MtCesA6-F TTGAACTTCGACTATAATCATTACATCAACAATAGCAAGGCTATTAGAGAAGCAATGTGT 1614

MsCesA6-F ------------------------------------------------------------

MtCesA6-F TTCATGATGGATCCATTAGTAGGAAAAAGGATTTGCTATGTTCAATTCTCCCAAAGATTT 1674

MsCesA6-F ------------------------------------------------------------

MtCesA6-F GATGGGATTGACAGTAATGACCAATATGCCAATCAAACAAATACATTTGTTGATATAAAC 1734

MsCesA6-F ------------------------------------------------------------

MtCesA6-F ATGAAAGGTTTGGATGGTATTCAAGGACCTACATATGTTGGAACAGGATGTGTATTTAGA 1794

MsCesA6-F ------------------------------------------------------------

MtCesA6-F AGACAAGCACTTTATGGTTTTGATGCTCCTAGGAAAAAGAAGGCACAAAACAAGACATGT 1854

MsCesA6-F ------------------------------------------------------------

MtCesA6-F AATTGTTGGCTAAAGTGTTGTTGTTGTGGATTGTGTTGTATGGGAAAGAGGAAGAAGAAA 1914

MsCesA6-F ------------------------------------------------------------

MtCesA6-F AAGATGAAGAAGTCTAAGTTTGAGTTAATGGATAGTACTCATAGAAAAGTACATTCTGAA 1974

MsCesA6-F ------------------------------------------------------------

MtCesA6-F TCATCTGTTGCTGGGAGTACTAAAGGAAATGAAAATGAAGATGGCTTGTCTATAATCTCA 2034

MsCesA6-F ------------------------------------------------------------

MtCesA6-F AGTCAAAAGTTAGTCAAGAAATTTGGAGAATCTCCAATTTTCATTGCATCTACTCAATTG 2094

MsCesA6-F ------------------------------------------------------------

MtCesA6-F GTTGATGGTGAGACACTAAAGCATGGTGGGATTGCTTCTCAACTTACAGAAGCAATACAT 2154

MsCesA6-F ------------------------------------------------------------

MtCesA6-F GTCATTAGTTGTGGCTATGAAGAGAAAACAGAATGGGGAAAAGAGGTTGGATGGATCTAT 2214

MsCesA6-F ------------------------------------------------------------

MtCesA6-F GGTTCAGTTACAGAAGATATATTAACAGGTTTCAAAATGCATTGTCATGGATGGAGATCT 2274

MsCesA6-F ------------------------------------------------------------

MtCesA6-F ATATATTGCATTCCTGAAAGAACTGCATTTAAAGTATCTTCATCAAATAACCTTTCTAAT 2334

MsCesA6-F ------------------------------------------------------------

MtCesA6-F GGTCTACAACAAGTCTTTCAATGGGCCCTTGGATCCATTGACATATTCATGAGTAAACAT 2394

MsCesA6-F ------------------------------------------------------------

MtCesA6-F TGTCCTATTTGGTATGGATATAAAGGAGGATTAAAGTGGTTAGAAAGAATTTCTTACATA 2454

MsCesA6-F ------------------------------------------------------------

MtCesA6-F AATGCTATTGTATATCCATGGACATCAATACCTTTGGTTGCATATTGTACTCTTCCTGCT 2514

MsCesA6-F ------------------------------------------------------------

MtCesA6-F GTTTGCTTGCTTACTGGAAAATTTATCATTCCTGAGCTTAGTAATACAGCTGGAATGTGG 2574

MsCesA6-F ------------------------------------------------------------

MtCesA6-F TTTATTTCTCTTTTCATTTGCATTTTCACAACAAGTATGCTAGAGATGAGGTGGAGTGGA 2634

MsCesA6-F ------------------------------------------------------------

MtCesA6-F GTTACAATTGATGAATGGTGGAGAAATGAGCAATTTTGGGTAATTGGAGGTGTTTCAGCA 2694

MsCesA6-F ------------------------------------------------------------

MtCesA6-F AATTTATATGCTGTATTTGTAGGTCTATTCAAAGTCCTAACAGGTGTAAATAGCAACTTC 2754

MsCesA6-F ------------------------------------------------------------

MtCesA6-F ATTGTAACATCAAAATCAACAAGAGATGATGAAGACAAAGAACACAATGAGATAATGTTT 2814

MsCesA6-F ------------------------------------------------------------

MtCesA6-F GGTTTGAAGTGGACAACATTATTAATCATACCAACAACATTGCTTATATTAAACATAATT 2874

MsCesA6-F ------------------------------------------------------------

MtCesA6-F GCTATGGTTGCTGGATTATCACATGCAATTAACAATGGTTTTGAATCATGGGGACCATTA 2934

MsCesA6-F ------------------------------------------------------------

MtCesA6-F TTTGGGAAACTAATGTTTTCCTTTTGGGTGATTGTTCACTTGTTTCCATTCCTTAAAGGT 2994

MsCesA6-F ------------------------------------------------------------

MtCesA6-F ATGACTGGAAGGAATAATAGAACTCCTACCATTGTTCTTGTTTGGTCAATCTTACTTGCA 3054

MsCesA6-F ------------------------------------------------------------

MtCesA6-F TCTTTCTTCTCTGTCTTGTGGGTAAAAATTGATCCATTTTTACCCAAGTCAACTGGTCCA 3114

MsCesA6-F ------------------------------------------------------------

MtCesA6-F ATTTTAGAAGAATGTGGATTGGATTGTAATTAG 3147

MsCesA6-F ---------------------------------

MtCesA7-B ATGGAAGCTAAGAGTGGTCTTTTTGCAGGGTCTCTTAACAGCAATGAACTTGTGGTCATC 60

MsCesA7-B ------------------------------------------------------------

MtCesA7-B CAAAAACAAAATGAGCCTAAGGCAGTGAAAAACTTGGATGGTCAAGATTGTGAGATATGT 120

MsCesA7-B ------------------------------------------------------------

MtCesA7-B GGTGATTCTGTAGGGCGTACAGTAGAAGGAGACTTATTTGTAGCTTGTGAAGAGTGTGGC 180

MsCesA7-B ------------------------------------------------------------

MtCesA7-B TTCCCTGTTTGCAGGCCATGCTATGAGTATGAAAGGAAAGAAGGGTCTCAAAATTGCCCT 240

MsCesA7-B ------------------------------------------------------------

MtCesA7-B CAATGCCATACAAGATACAAGCGTATCAAAGGAAGTCCAAGAGTGGAGGGAGATGAAGAT 300

MsCesA7-B ------------------------------------------------------------

MtCesA7-B GAAGAAGATGTGGATGATATTGAACAAGAATTTAAAATGGAAGAGGAAAAGTACAAGCTT 360

MsCesA7-B ------------------------------------------------------------

MtCesA7-B ATGCATCAAGATAATATGAATAGCATTGATGATGATGATACAAAATATAGGGAGCAACCC 420

MsCesA7-B ------------------------------------------------------------

MtCesA7-B CTATATTCTCACAGTATTGGAGAAAATTATGGTGCAAAATTAGATAACAAGGAGAAAACA 480

MsCesA7-B ------------------------------------------------------------

MtCesA7-B GATGAGTGGAAGCAGCAAGGAAATCTGTTGATTGAAACTGACGCGGTTGATCCAGAAAAG 540

MsCesA7-B ------------------------------------------------------------

MtCesA7-B GCCATGAAAGATGAAACTAGACAGCCACTATCAAGGAAAGTAGCAATACCTTCAGGAAGA 600

MsCesA7-B ------------------------------------------------------------

MtCesA7-B CTCAGTCCTTATAGAATGATGGTTGTGGCTAGGCTCATCCTTCTATTACTCTTCTTTGAG 660

MsCesA7-B ------------------------------------------------------------

MtCesA7-B TACAGAATCTCCCATCCAGTACCTGATGCAATTGGACTTTGGTTTATATCGGTATCATGT 720

MsCesA7-B -----------------------------AATTGGACTTTGGTTCATATCAGTATCATGT 31

*************** *****.*********

MtCesA7-B GAAATCTGGCTTGCATTGTCATGGATAGTTGATCAGATTCCCAAATGGTTTCCCATCGAT 780

MsCesA7-B GAAATCTGGCTTGCATTGTCATGGATACTTGATCAGATTCCCAAATGGTTTCCCATCGAT 91

*************************** ********************************

MtCesA7-B CGCGAGACATACCTTGACCGCCTTTCAGTCAGGTTTGAGCCGGAAAACAAGCCCAACATG 840

MsCesA7-B CGCGAGACATACCTTGACCGCCTTTCAGTCAGGTTTGAGCCGGAAAACAAGCCTAATATG 151

***************************************************** ** ***

MtCesA7-B CTTTCTCCAATAGATATTTTTATAACAACCGCAGATCCAATCAAGGAACCACCTCTTGTT 900

MsCesA7-B CTTTCTCCAATAGATATCTTTATAACAACCGCAGATCCAATCAAGGAACCACCTCTTGTT 211

***************** ******************************************

MtCesA7-B ACAGCAAATACTGTTCTTTCAATTTTGGCACTAGATTATCCTGCAAACAAAATTTCATGT 960

MsCesA7-B ACAGCAAATACTGTTCTTTCAATTTTGGCACTAGATTATCCTGCAAACAAAATTTCATGT 271

************************************************************

MtCesA7-B TACGTTTCTGATGACGGCGCTTCCATGCTCACCTTTGAAGCTCTTCAAGAAACAGCTGAA 1020

MsCesA7-B TACGTTTCTGATGACGGCGCTTCCATGCTCACCTTTGAAGCACTTCAAGAAACAGCTGAA 331

*****************************************:******************

MtCesA7-B TTCGCGCAAAAATGGGTACCTTTCTGTAAACAATTCTCTACAGAGCCGCGAGCACCTGAG 1080

MsCesA7-B TTTGCGCAAAAATGGGTACCTTTCTGTAAACAATTCTCTACCGAGCCGCGTGCACCTGAG 391

** **************************************.********:*********

MtCesA7-B AAGTATTTCTCTGAGAAGATAGACTTTCTTAAGGATAAGCTTCAACCCACATACGTAAAA 1140

MsCesA7-B AAGTATTTCTCTGAGAAGATAGACTTTCTTAAGGATAAGCTTCAACCGACATACGTAAAA 451

*********************************************** ************

MtCesA7-B GAACGCCGTGCTATGAAGAGAGAATATGAAGAGTTTAAGGTGAGAATAAATGCGCTTGTG 1200

MsCesA7-B GAACGCCGTGCTATGAAGAGAGAATATGAAGAGTTTAAGGTGAGAATAAATGCGCTTGTG 511

************************************************************

MtCesA7-B GCTAAATCTATGAGAGTTCCATCAGAAGGTTGGAGTATGAAGGATGAAACACCATGGCCG 1260

MsCesA7-B GCTAAATCTATGAGAGTTCCATCAGAAGGTTGGAGTATGAAAGATGAAACACCATGGCCA 571

*****************************************.*****************.

MtCesA7-B GGAAACAATACAAAAGATCATCCAAGTATGATACAAATACTTCTTGGTCACAATGGAGGA 1320

MsCesA7-B GGAAACAACACAAAAGATCATCCAAGTATGATACAAATACTTCTTGGTCACAATGGAGGA 631

******** ***************************************************

MtCesA7-B GACAGTGAAGGAAATGAACTTCCATCTCTTGTCTACATTTCTAGAGAAAAAAGACCTGCA 1380

MsCesA7-B GACAATGAAGGAAATGAACTTCCATCTCTNGTCTACATTTCTAGAGAGAAAAGACCTGCA 691

****.************************.*****************.************

MtCesA7-B TTTCAACATCACACAAAGGCCGGTGCAATGAACGCCTTGCTTCGTGTATCGGCAGTATTG 1440

MsCesA7-B TTTCAACATCACACAAAAGCCGGTGCAATGAACGCCTTGCTTCGTGTATCGGCAGTATTG 751

*****************.******************************************

MtCesA7-B AGCAATGCTCCTTTTGTGCTCAACTTGGACTGCAATCATTATGTGAATTACAGCAAAGTT 1500

MsCesA7-B AGCAATGCTCCTTTTGTGCTCAACTTGGACTGCAATCATTATGTGAATTACAGCAAAGTT 811

************************************************************

MtCesA7-B GTGAGAGAAGCCATGTGTTTCTTTATGGACATTCAACTTGGGAATAGTATTGCCTTTGTT 1560

MsCesA7-B GTGAGAGAAGCCATGTGTTTCTTTATGGACATTCAACTTGGGAATAGTATTGCTTTTGTT 871

***************************************************** ******

MtCesA7-B CAGTTTCCACTAAGATTTGATAGTCTTGATAGGAATGATCGTTATGCCAACAAAAACACT 1620

MsCesA7-B CAGTTTCCACTGAGATTTGATAGTCTTGATAGGAACGATCGTTATGCCAACAAAAACACT 931

***********.*********************** ************************

MtCesA7-B ATTTTATTTGATATCAACTTGAGGTGCCTAGATGGAATTCAAGGACCTGTTTATATTGGA 1680

MsCesA7-B ATTTTATTTGATATCAACTTGAGGTGTCTAGATGGAATTCAAGGACCTGTTTATATTGGA 991

************************** *********************************

MtCesA7-B TCAGGTTGTATATTCAGAAGGAAAGCTTTAAATGGCTTTGATCCTCCTAAGGCGTCAAAA 1740

MsCesA7-B TCAGGTTGTATATTCAGAAGGAAAGCTTTAAATGGCTTT--------------------- 1030

***************************************

MtCesA7-B CGCTCTCGAGTCGTACAAGTTCACTCAAAACAGGATGAAAATGAAGAGGATGGGAGCATT 1800

MsCesA7-B ------------------------------------------------------------

MtCesA7-B ATAGAAGCAACTGATGAAGAGAAGCAGCCATTGCAATTAGATAAGGATACTGAAAACAAA 1860

MsCesA7-B ------------------------------------------------------------

MtCesA7-B TTTGGAAAGTCTACACTCTTCATGAATTCTTCATTGACAGAAGAAGGTGGCGTAGATCCT 1920

MsCesA7-B ------------------------------------------------------------

MtCesA7-B TCTTCAACTCAAGAAGTCCTGCTTAAAGAGGCCATTCATGTCATGAGTTGTAGCTACGAA 1980

MsCesA7-B ------------------------------------------------------------

MtCesA7-B GACCGGACACTGTGGGGATACGAGGTTGGTATGAGCTATGGATCTATAGCATCAGATATT 2040

MsCesA7-B ------------------------------------------------------------

MtCesA7-B CTAACAAGTTTAAAGATGCATACTCGTGGTTGGAGATCGGTATACTGCATGCCGAAAAGA 2100

MsCesA7-B ------------------------------------------------------------

MtCesA7-B GCTCCTTTTAGGGGAACAGCCCCAATCAATCTTACAGAAAGACTTAACCAAGTTCTTAGA 2160

MsCesA7-B ------------------------------------------------------------

MtCesA7-B TGGGCAGTAGGATCACTTGAGATTCTATTCAGCCACCACTGTCCAATATGGTATGGTTTT 2220

MsCesA7-B ------------------------------------------------------------

MtCesA7-B AAAGAAGGAAGACTCAAGCTGCTCCAAAGGATTGCCTATATTAACAGCACTGTCTATCCT 2280

MsCesA7-B ------------------------------------------------------------

MtCesA7-B TTTAGTGCATTGCCTCTCATAATATATTGTATCGTTCCGGCTGTCTGCTTGCTCACTGAT 2340

MsCesA7-B ------------------------------------------------------------

MtCesA7-B AAATTCATCACACCATCGGTAGGAACTTTTGCAAGTCTGGTATTCATTTCTCTATTCATA 2400

MsCesA7-B ------------------------------------------------------------

MtCesA7-B TCAATCTTCGCCTCTTCTATTCTTGAATTGAGATGGAGTGGAGTTAGTCTTGAGGAATGG 2460

MsCesA7-B ------------------------------------------------------------

MtCesA7-B TGGAGAAATCAACAATTTTGGGTCATTGGAAGTATATCAGCACATCTCTTTGCAATTGTA 2520

MsCesA7-B ------------------------------------------------------------

MtCesA7-B CAAGGCCTAATGGGAAGATTCCTTGGTAGATTTAATGCACATTTCAACATTGTATCGAAG 2580

MsCesA7-B ------------------------------------------------------------

MtCesA7-B GCACCAGACGATGATGGCGAGTTTAACGAACTGTACACCATTAGATGGACAGTACTACTA 2640

MsCesA7-B ------------------------------------------------------------

MtCesA7-B ATACCTCCAACCACAGTGACTATATTCAACATTATCGGTATTGTTGCTGGTTTCACAGAT 2700

MsCesA7-B ------------------------------------------------------------

MtCesA7-B GCTATAAATAGTGGTGAACATGAATGGGGAGCATTGATTGGGAAACTGTTCTTTTCTTCA 2760

MsCesA7-B ------------------------------------------------------------

MtCesA7-B TGGGTTATTGCTCATCTTTATCCTTTCCTTAAAGGACTAATGGGAAGACAGAATAGAACA 2820

MsCesA7-B ------------------------------------------------------------

MtCesA7-B CCAACTCTTGTTGTCATTTGGTCTGTGCTTTTGGCTTCTATCTTTTCTTTGGTTTGGGTA 2880

MsCesA7-B ------------------------------------------------------------

MtCesA7-B AGAATCGACCCTTTTGTGTTGAAAACTAAGGGGCCTGATGTTAAGCAATGTGGAATTAGT 2940

MsCesA7-B ------------------------------------------------------------

MtCesA7-B TGTTGA 2946

MsCesA7-B ------

MsCesA8 ------------------------------------------------------------

MtCesA8 ATGATGCCATCTGGTGCTTCCCTCTGCAACATTTGTGGGGAACAACTAGTGCTTAGTGAG 60

MsCesA8 ------------------------------------------------------------

MtCesA8 AATGGGGAATTGTTTGTGGCTTGTCATGAGTGTAGCTACCCAATTTGTAAGGCCTGTTTT 120

MsCesA8 ------------------------------------------------------------

MtCesA8 GAACATGAAATCAATGAGGGACATAAGGTCTGTCTTAAGTGTGGCACTCCCTATGAAGGG 180

MsCesA8 ------------------------------------------------------------

MtCesA8 AGAACAAATAATGATAATGTTGATGATGAGCGCGAAGACGACGACGATGACATCATGGTT 240

MsCesA8 ------------------------------------------------------------

MtCesA8 CATGAAAATCCATCTACAATGGCTTCCCAGATCAATAACTCTGAGGATGGTGGGGGACTT 300

MsCesA8 ------------------------------------------------------------

MtCesA8 CATGCTAGACATATCAGTACAGTGTCCTCACTTGATATTGAAGAAGTAAATGAAGAATCT 360

MsCesA8 ------------------------------------------------------------

MtCesA8 GGGAATTCAAAATGGAAAAATAGAATGAAAGGCTGGAAAGGGAAAGGGAAGGGGAAAGGG 420

MsCesA8 ------------------------------------------------------------

MtCesA8 AAGGGGAAAGACAAAAAGAACAAGACTAAAAAGGATGCACCTACAGCTGAAAATGAGGCT 480

MsCesA8 ------------------------------------------------------------

MtCesA8 GCAGTTCCACCGGAGCAGCAGATGGAAGAAATTCGGTCCACAGATGCTGCTGCTCTGCCA 540

MsCesA8 ------------------------------------------------------------

MtCesA8 CTTTCAGTACTTATGCCAATAGTAAAGTCCAAACTCGCACCATACAGAACTGTGATAATT 600

MsCesA8 ------------------------------------------------------------

MtCesA8 GTGCGGCTAGTAATCTTGGGTCTTTTCTTCCATTATCGAGTTACAAATCCTGTTGAAAGT 660

MsCesA8 ------------------------------------------------------------

MtCesA8 GCTTTTCCTCTGTGGTTGACATCTATTATATGTGAGATTTGGTTTGCATTTTCCTGGGTG 720

MsCesA8 ------------------------------------------------------------

MtCesA8 TTGGATCAGTTCCCTAAATGGTCTCCAGTTAATCGACACACTTATATTGAGAACCTCTCT 780

MsCesA8 ------------------------------------------------------------

MtCesA8 GCAAGGTTTGAAAGAGAGGGTGAACCCTCTGGACTTGCTTCTGTTGATTTTTTTGTCAGT 840

MsCesA8 ------------------------------------------------------------

MtCesA8 ACAGTCGATCCTTTGAAAGAACCACCACTGATTACAGCTAATACAGTGCTTTCTATCCTT 900

MsCesA8 ------------------------------------------------------------

MtCesA8 GCGGTTGACTATCCAGTAGATAAAGTATCTTGTTATGTGTCAGATGATGGAGCAGCAATG 960

MsCesA8 ------------------------------------------------------------

MtCesA8 CTCACATTTGAATCTCTTGTGGAGACAGCTGAATTTGCAAAGAAGTGGGTGCCATTTTGC 1020

MsCesA8 ------------------------------------------------------------

MtCesA8 AAAAAGTTTTCAATTGAACCTAGAGCACCTGAGTATTACTTCTCACAAAAAATTGACTAC 1080

MsCesA8 ------------------------------------------------------------

MtCesA8 CTTAAAGATAAAGTGCAACCTTCTTTTGTAAAAGAACGTAGAGCAATGAAGAGAGAATAT 1140

MsCesA8 ------------------------------------------------------------

MtCesA8 GAAGAGTATAAAGTACGTGTTAATGCTATGGTAGCTAAGGCCCAGAAAACACCAGAAGAA 1200

MsCesA8 ------------------------------------------------------------

MtCesA8 GGATGGACGATGCAAGATGGAACACCTTGGCCTGGCAATAACTCACGTGATCACCCTGGA 1260

MsCesA8 -----------------------------------------------------ACTTCCT 7

MtCesA8 ATGATTCAGGTTTTCCTCGGACACAGTGGTGCTCGTGATATCGAAGGAAATGAACTTCCT 1320

*******

MsCesA8 AGGCTAGTTTATGTTTCTAGAGAGAAAAGACCAGGATACCAACACCACAAGAAAGCAGGT 67

MtCesA8 AGGCTAGTTTATGTTTCTAGAGAGAAAAGACCAGGATACCAACATCACAAGAAAGCAGGT 1380

******************************************** ***************

MsCesA8 GCTGAAAACGCACTGGTGAGGGTGTCTGCAGTTCTCACAAATGCTCCCTTCATTCTCAAT 127

MtCesA8 GCTGAAAATGCACTGGTGAGGGTGTCTGCAGTTCTCACAAATGCTCCCTTCATTCTCAAT 1440

******** ***************************************************

MsCesA8 CTTGATTGTGATCATTATGTTAACAACAGCAAGGCTGTTCGAGAAGCAATGTGTTTTCTC 187

MtCesA8 CTTGACTGTGATCATTATGTTAACAACAGCAAGGCTGTCCGAGAAGCAATGTGTTTTCTC 1500

***** ******************************** *********************

MsCesA8 ATGGATCCAGAAGTTGGTAGAGATGTTTGTTATGTACAATTCCCCCAAAGATTTGATGGT 247

MtCesA8 ATGGATCCAGAAGTTGGTAGAGACGTTTGTTATGTACAATTCCCCCAAAGATTTGATGGT 1560

*********************** ************************************

MsCesA8 ATTGACCGTAGTGATCGATATGCCAACCGCAATACAGTTTTCTTTGATGTAAACATGAGA 307

MtCesA8 ATTGATCGCAGTGATCGATATGCCAACCGCAATACAGTTTTCTTTGATGTAAACATGAGA 1620

***** ** ***************************************************

MsCesA8 GGACTTGATGGCATTCAAGGACCAATGTATGTGGGGACTGGTTGTGTTTTCAATCGGCAA 367

MtCesA8 GGACTTGATGGCATTCAAGGACCAATGTATGTGGGGACTGGATGTGTTTTCAACCGACAA 1680

*****************************************:*********** **.***

MsCesA8 GCACTTTATGGCTATAGCCCACCTTCTATGGTCAATTCACCAATGTCTTCATGCTGTTGC 427

MtCesA8 GCACTTTATGGCTATAGCCCACCTTCTATGGTCAATTCACCAATATCATCATGCTGTTGC 1740

********************************************.**:************

MsCesA8 TGCCCCTCCACCAAAGAAGTGTCACGGGTTTCTAGAGATGGAAAAAGGGCAGAACTTGAT 487

MtCesA8 TGCCCCTCCAGCAAAGAAGTGTCAAGGGTTTCTAGAGATGGAAAGAGGGCAGAACTTGAT 1800

********** *************.*******************.***************

MsCesA8 GCTGCAATTTATAATCTCAGGGAGATTGATAATTATGATGAGAATGAGAGGTCAATGCTA 547

MtCesA8 GCTGCAATTTATAATCTCAGGGAGATTGACAATTATGATGAGAATGAGAGGTCAATGCTA 1860

***************************** ******************************

MsCesA8 ATTTCACAAATGAGCTTTGAAAAAACTTTTGGCTTGTCTACTGTTTTCATTGAATCTGCA 607

MtCesA8 ATTTCACAAATGAGCTTTGAAAAAACTTTTGGCTTGTCTACTGTTTTCATTGAATCTGCA 1920

************************************************************

MsCesA8 TTAATGGAGAATGGAGGAGGGGTACCAGAATCTGCAGATCCTTCAATGCTGATCAAGGAG 667

MtCesA8 TTAATGGAGAACGGAGGAGGGGTACCAGAATCTGCAGATCCTTCAATGCTGATCAAGGAG 1980

*********** ************************************************

MsCesA8 GCCATTCATGTAATTAGCTGTGGGTATGAAGAGAAGACTGAATGGGGAAAAGAGATTGGT 727

MtCesA8 GCCATTCATGTAATTAGCTGTGGGTATGAAGAGAAGACTGAATGGGGGAAAGAGATTGGT 2040

***********************************************.************

MsCesA8 TGGATTTATGGTTCAGTTACTGAGGATATCTTAACAGGGTTCAAGATGCA---------- 777

MtCesA8 TGGATTTATGGTTCAGTTACTGAGGATATCTTAACAGGATTCAAGATGCAGTGCCGAGGA 2100

**************************************.***********

MsCesA8 ------------------------------------------------------------

MtCesA8 TGGAGGTCGATCTACTGCATGCCCTTAAGGCCTGCATTCAAAGGGTCAGCACCTATCAAC 2160

MsCesA8 ------------------------------------------------------------

MtCesA8 TTGTCTGATCGGTTGCACCAAGTCCTTCGATGGGCGCTTGGATCAGTTGAAATTTTCTTA 2220

MsCesA8 ------------------------------------------------------------

MtCesA8 AGTAGACATTGCCCCCTTTGGTATGCAGTTGGAGGAGGCCGTCTCAAATGGCTGCAGAGA 2280

MsCesA8 ------------------------------------------------------------

MtCesA8 TTGGCTTATATAAACACTATTGTTTATCCTTTTACATCCCTTCCTCTAGTTGCTTATTGT 2340

MsCesA8 ------------------------------------------------------------

MtCesA8 ACTTTGCCAGCAATTTGCCTTCTCACAGGAAAATTTATCATACCAACGCTTACCAACGTT 2400

MsCesA8 ------------------------------------------------------------

MtCesA8 GCAAGTATTCTCTTTCTTGGACTTTTCCTCTCCATTATAGTGACAAGTGTGCTTGAGCTG 2460

MsCesA8 ------------------------------------------------------------

MtCesA8 CGTTGGAGTGGTGTTTGTATTGAAGATTTATGGCGTAATGAGCAGTTTTGGGTGATTGGA 2520

MsCesA8 ------------------------------------------------------------

MtCesA8 GGTTCTTCAGCTCATCTGTTTGCTGTGTTCCAAGGATTTCTTAAGATGTTGGCTGGTGTT 2580

MsCesA8 ------------------------------------------------------------

MtCesA8 GATACCAACTTCACTGTCACTGCCAAGGCTGCTGAGGATACCGAGTTTGGTGAACTTTAT 2640

MsCesA8 ------------------------------------------------------------

MtCesA8 ATTATCAAGTGGACTACTCTCTTGATTCCACCTACAACTCTTATCATTATTAACATGGTT 2700

MsCesA8 ------------------------------------------------------------

MtCesA8 GGTGTTGTTGCTGGATTTTCTGATGCACTTAATGGAGGATATGAGTCTTGGGGACCTCTC 2760

MsCesA8 ------------------------------------------------------------

MtCesA8 TTTGGGAAGGTTTTCTTTGCCTTCTGGGTGATTTTTCATCTCTATCCATTCCTCAAGGGT 2820

MsCesA8 ------------------------------------------------------------

MtCesA8 CTCATGGGGCGCCAAAACCGCACTCCTACCATTGTTATTCTGTGGTCAGTGTTGTTAGCC 2880

MsCesA8 ------------------------------------------------------------

MtCesA8 TCTGTCTTTTCTATTATTTGGGTCAAGATAGACCCATTTGTGAACAAAGTTGACAGTGAG 2940

MsCesA8 ------------------------------------

MtCesA8 ACCATAGCTGAAACTTGTGTTGCTATAGATTGTTAA 2976

**Figure S2**
