# Supplementary material for: Alfalfa Cellulose Synthase Gene Expression under Abiotic Stress: A Hitchhiker’s Guide to RT-qPCR Normalization
Source: PLoS One. 2014 Aug 1;9(8):e103808. doi: 10.1371/journal.pone.0103808 (PMC4118957; doi:10.1371/journal.pone.0103808)
Supplement: Figure S3 — Sequence details of MtCESA6-F. Alignment of MtCESA6-F with CesAs from C. aretinum [GenBank: XP_004499618.1] and P. vulgaris [GenBank: ESW20735.1] showing the amino acid substitutions in the processive GT2s motif (bold and underlined). The zinc-finger domain (CxxC)4 is highlighted in yellow. (DOC) [file pone.0103808.s003.doc]

XP_004499618.1 METNLGLVAGSHNNNEFIIIRQDGDFARKELQELDGKTCQLCEEEIKVNEEGDPFVACNE 60

MtCESA6-F METNFGLVAGSHNKNEFIIIRQDGDYARTDLQELDGDTCQLCGEDIGVNADGDPFVACNE 60

ESW20735.1 METNLGLVAGSHNSNEFIIIPQDG--VQRRLKEIDGRRCELCGDDIGVNAEGEVFVACNE 58

****:********.****** *** .: *:*:** *:** ::* ** :*: ******

XP_004499618.1 CAFPVCRNCYEYERREGNKVCPQCKTRFKRLKGCARVEGDEEEDGIDDMENEFDFDERNI 120

MtCESA6-F CAFPVCRNCYEYERREGNQVCPQCKTKFKRLKGCARVEGDEEEDDIDDLENEFDEGR--- 117

ESW20735.1 CGFPVCKSCYEYERREGNHVCPQCKTRFKRLKGCARVEGDEEDDDIDELENAEDIDGG-- 116

*.****:.**********:*******:***************:*.**::** * .

XP_004499618.1 DEQEMQIILDHEGEEVS-EEHHAIVPLN--STMKKEITLLQARPMDPSKDLASYGYGSVA 177

MtCESA6-F NEQDMQIPMSPEGEELSSEEHHAIVPLINSTIMRKEITLLQARPMDPSKDLAAYGYGSVA 177

ESW20735.1 NKQDFNTTMSHEDEETC-EEHDALVPSS-STTLSKEMVPLQARSMDPSKDLAAYGYGSVA 174

::*::: :. *.** . ***.*:** : : **:. ****.********:*******

XP_004499618.1 WKERMEIWKQRQDKLGNMRKENN---YDKAMVDDNEFPLMDESRQPLSRKLAIPSSKINP 234

MtCESA6-F WKDRMELWKQRQNQLGNMRKDDNE-DLDKSVDDDNEFPLMDESRQPLSRKLPIPSSQINP 236

ESW20735.1 WKERMKIWKQRQMELGNVRKQNDEEDPNNLVDDDTEFLIMDEGRQPLSRKLSVPSSKINP 234

**:**::***** :***:**::: :: : **.** :***.********.:***:***

XP_004499618.1 YRLIIILRVVVIGFFFHYRIMHPVENAYALWVVSLICEIWFTLSWILHQFSMWFPVTRQT 294

MtCESA6-F YRMIIIIRLIVLGFFFQYRIMHPVDNAYALWLVSVICEIWFTLSWILDQFSKWFPVMRET 296

ESW20735.1 YRMVILMRLVVLGFFFHYRIVHPVEHAYALWLVSIICEIWFTLSWILDQFPKWLPVIRQT 294

**::*::*::*:****:***:***::*****:**:************.**. *:** *:*

XP_004499618.1 YLDRLSLRYEKEGEQSQLCPIDIFVTTMDPLKESPLVTANTVLSILAI**D**YPVEKVSCYVS 354

MtCESA6-F YLDRLSLR--QEGQPSQLSPIDIFVTTNDPLKESPLVTANTVLSILAI**D**YPAEKVSCYVS 354

ESW20735.1 YLDRLSLR--QEGKPSQLSPIDIFVITVDPLKEPPLVTANTVLSILAM**D**YPAEKVSCYVS 352

******** :**: ***.****** * *****.*************:***.********

XP_004499618.1 DDGAAMLTFEALSETYEFARKWVPFCKKFNIEPRAPECYFAEKINYLNDKVHSSFVKERR 414

MtCESA6-F DDGAAMLTFEALSETSEFARKWVPFCKKFNIEPRAPEWYFHEKINYLKDKVHSSFVKERR 414

ESW20735.1 DDGAAMLTFEALSETCEFAKKWVPFCKKFSIEPRAPEWYFAEKINYLNDKVHPSFVKERR 412

*************** ***:*********.******* ** ******:****.*******

XP_004499618.1 AMKREYEEFRVRMNCLVAKAKKVPEEGWTMQDGTPWPGNNISDHPGMIQVFLGENEGYDM 474

MtCESA6-F AMKREYEEFKVRINSLVAKAKKVPEEGWTMQDGMLWPGNNIRDHPGMIQVFLGENGGCDM 474

ESW20735.1 AMKREYEEFRVGINSLVAKSRKVPEEGWTMQDGTPWPGNNVGDHPGMIQVFLGENGGYDM 472

*********:* :*.****::************ *****: ************* * **

XP_004499618.1 NGNELPRLVYVSREKRPNFNHQRKAGALNALVRASAVLSNAPFVLNL**DYN**HCINNSKAIR 534

MtCESA6-F DGNELPRLVYVSREKRPNFNHQKKAGALNALVRVSSVLSNAPFVLNF**DYN**HYINNSKAIR 534

ESW20735.1 DGKELPRLIYVSREKRPKFNHQKKAGALNALVRVSAVLSNAPFVLNL**DYD**HYINNSKVVR 532

:*:*****:********:****:**********.*:**********:**:* *****.:*

XP_004499618.1 EAMCFMMDPLLGKRVCYVQFSQRFDAIHSNDQYANQTNSFVDINMKGLDGIQGPTYVGTG 594

MtCESA6-F EAMCFMMDPLVGKRICYVQFSQRFDGIDSNDQYANQTNTFVDINMKGLDGIQGPTYVGTG 594

ESW20735.1 EAMCFMMDPVVGKRVSYVQFSQRFDGI---EQHANQTNGFFDINMKGLDGIQGPTYVGTG 589

*********::***:.*********.* :*:***** *.*******************

XP_004499618.1 CVFRRQALYGFDAPRKKKSPNKTCNCWLKCCCG-LCCMGKRKKKKLKKSKIEIMEGSHRK 653

MtCESA6-F CVFRRQALYGFDAPRKKKAQNKTCNCWLKCCCCGLCCMGKRKKKKMKKSKFELMDSTHRK 654

ESW20735.1 CVFRRKALYGFDSPRKKKPPTKTCNCWPKWCCC----MGKKKKKKLKKPKFEIMENSHSK 645

*****:******:*****. .****** * ** ***:****:**.*:*:*:.:* *

XP_004499618.1 KEVHSQSSIDGSIKGNE--DVLVSISSQKFVKKFGQSPIFIASTQLLDGETLKNGSLASQ 711

MtCESA6-F --VHSESSVAGSTKGNENEDGLSIISSQKLVKKFGESPIFIASTQLVDGETLKHGGIASQ 712

ESW20735.1 --VHSEASIVEGTEDET----LAHISNPKFAKKFGQSPIFIASTQLVEGETLKHGNLASQ 699

***::*: . :.: * **. *:.****:**********::*****:*.:***

XP_004499618.1 LTEAIHVISCGYEEKTEWGKEVGWIYGSVTE**D**ILTGFKMHCHGWRSIYCIPKRNAFKVSS 771

MtCESA6-F LTEAIHVISCGYEEKTEWGKEVGWIYGSVTE**D**ILTGFKMHCHGWRSIYCIPERTAFKVSS 772

ESW20735.1 LTEAIHVISCGYEEKTEWGKEVGWIYGSVTE**D**ILTGFKMHCHGWRSIYSIPERPGFKVSS 759

************************************************.**:* .*****

XP_004499618.1 PTNLCNGLQ**QVFKW**ALGSIDILMSKHCPIWYGYKGGLKWLERISYINAIVYPLTSIPLVA 831

MtCESA6-F SNNLSNGLQ**QVFQW**ALGSIDIFMSKHCPIWYGYKGGLKWLERISYINAIVYPWTSIPLVA 832

ESW20735.1 PRNLSNGLQ**QVFQW**ALGSMEIFMSKHCPLWYGYGGGLKWLQRISYINVIVYPWTSIPLVV 819

. **.*******:*****::*:******:**** ******:******.**** ******.

XP_004499618.1 YCTLPAVCLLTGKFIIPELNNTAGMWFISLFICIFSTSVLEMRWSGVTIDEWWRNEQFWI 891

MtCESA6-F YCTLPAVCLLTGKFIIPELSNTAGMWFISLFICIFTTSMLEMRWSGVTIDEWWRNEQFWV 892

ESW20735.1 YCTLPAVCLLTGKFIIPELSNTAGMWFVSLFFCIFTTSVLEMRWSGVTVDEWWRNEQFWV 879

*******************.*******:***:***:**:*********:**********:

XP_004499618.1 IGGVSANLFAVFVGLFKLITGVNSNFIVTSKS-REDEEKEHNE-MFGLKWTTLLIIPTTL 949

MtCESA6-F IGGVSANLYAVFVGLFKVLTGVNSNFIVTSKSTRDDEDKEHNEIMFGLKWTTLLIIPTTL 952

ESW20735.1 IGGVSAHFLAVFLGMFKVLAGVNTNFIVTSRE---DDEKQHSD-MFALNWTTLLIIPTTL 935

******:: ***:*:**:::***:******:. *::*:*.: **.*:***********

XP_004499618.1 LILNIIAMVAGVSHAINNGFVSWGPLFGKLIFSFWVILHLYPFLKGMLGRNNRTPTIVLV 1009

MtCESA6-F LILNIIAMVAGLSHAINNGFESWGPLFGKLMFSFWVIVHLFPFLKGMTGRNNRTPTIVLV 1012

ESW20735.1 LILNIIAVVAGISNAINIGFEAWGPLLGKLLFSLWVILHLYPFLKGMVGRHNRTPTIVVV 995

*******:***:*:*** ** :****:***:**:***:**:****** **:*******:*

XP_004499618.1 WSILLASLFSVLWVKIDPFLPKTNGPILEECGLDCN 1045

MtCESA6-F WSILLASFFSVLWVKIDPFLPKSTGPILEECGLDCN 1048

ESW20735.1 WAILLATFFSVLWVKIDPFLPKSSGPILEECGLDCT 1031

*:****::**************:.***********.

**Figure S3**
