# Supplementary figures and images for: Alfalfa Cellulose Synthase Gene Expression under Abiotic Stress: A Hitchhiker’s Guide to RT-qPCR Normalization
Source: PLoS One. 2014 Aug 1;9(8):e103808. doi: 10.1371/journal.pone.0103808 (PMC4118957; doi:10.1371/journal.pone.0103808)

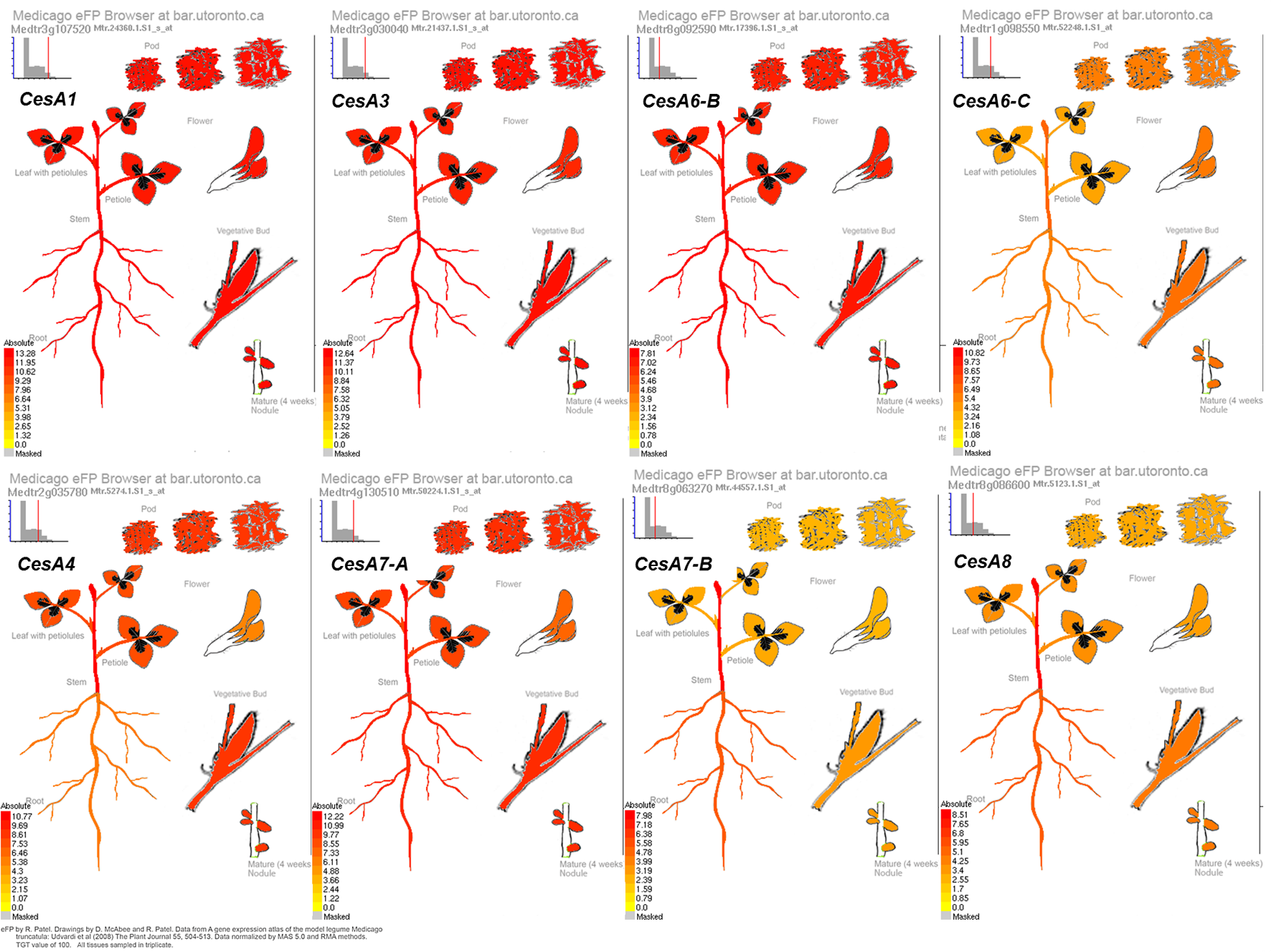

Supplement: Figure S4 — Electronic Fluorescence Pictographic (eFP) representations of M. truncatula CesA1, CesA3, CesA6-B, CesA6-C, CesA4, CesA7-A, CesA7-B, CesA8. (TIF) [file pone.0103808.s004.tif]

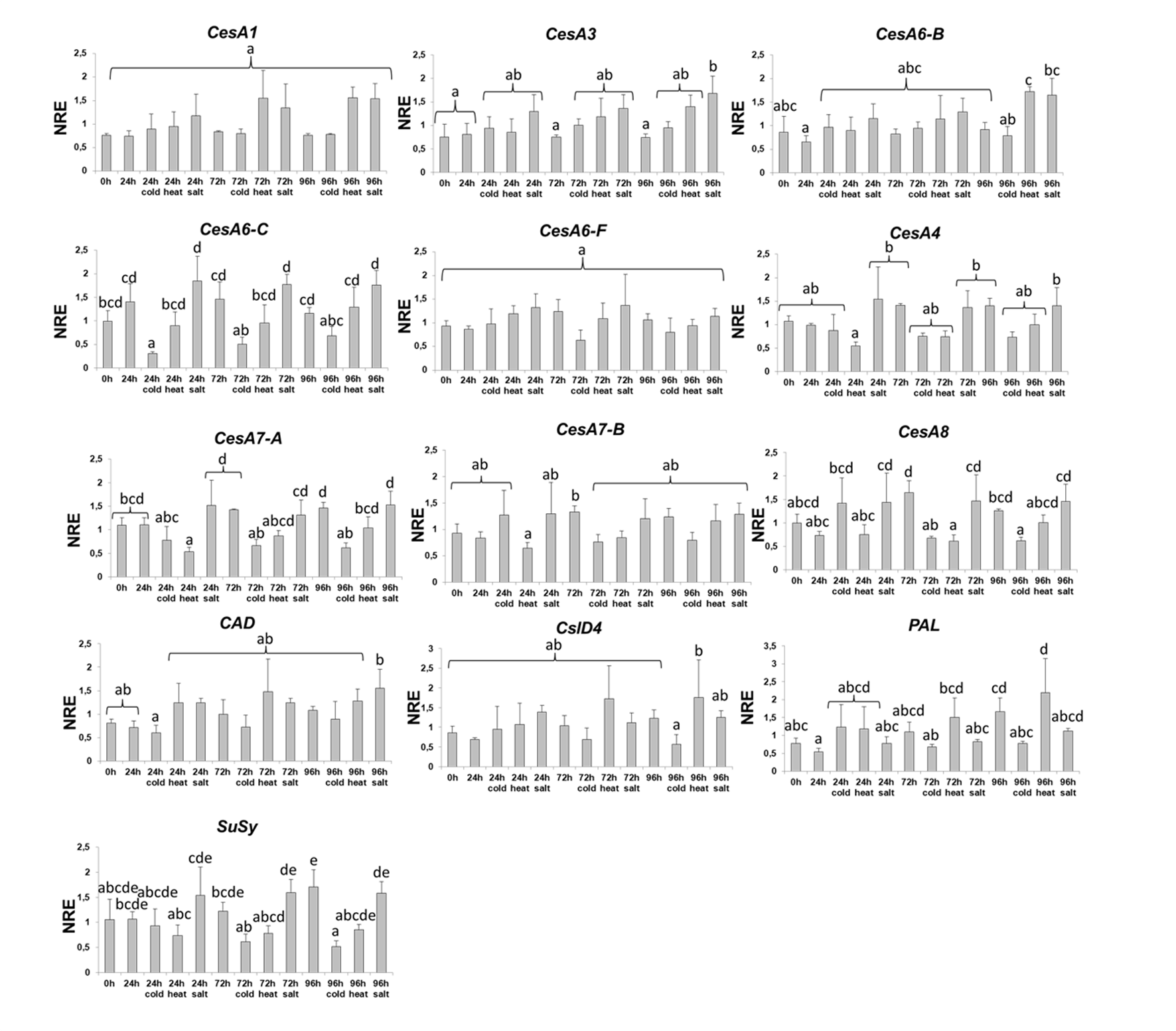

Supplement: Figure S5 — Gene expression profiles of cell wall-related genes in stems of alfalfa plants subjected to abiotic stress. Data were normalized using eif4A/TFIIA. Means sharing a letter are not significantly different at α = 0.05. NRE indicates Normalized Relative Expression. (TIF) [file pone.0103808.s005.tif]

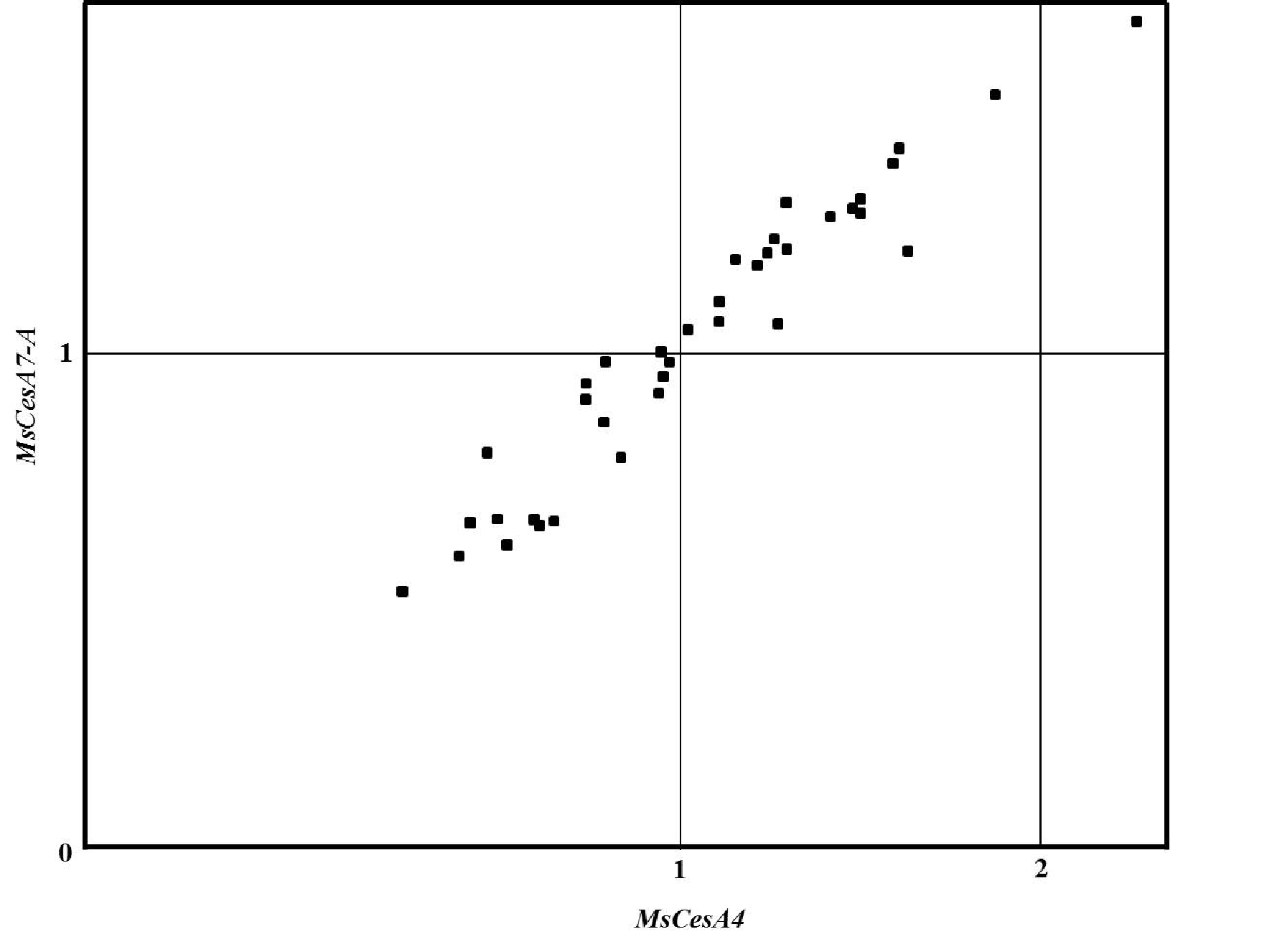

Supplement: Figure S6 — MsCesA7-A and MsCesA4 relationship. Correlation between MsCesA7-A and MsCesA4 in stems under abiotic stress conditions. Pearson (log) r = 0.962; Spearman (log) r = 0.961. (TIF) [file pone.0103808.s006.tif]
