# Supplementary material for: Alfalfa Cellulose Synthase Gene Expression under Abiotic Stress: A Hitchhiker’s Guide to RT-qPCR Normalization
Source: PLoS One. 2014 Aug 1;9(8):e103808. doi: 10.1371/journal.pone.0103808 (PMC4118957; doi:10.1371/journal.pone.0103808)
Supplement: Table S1 — List of primers used to amplify MsCesAs. Name of the primers, with the respective sequences, used to amplify the CesAs from M. sativa. (DOC) [file pone.0103808.s007.doc]

| **Name** | **Sequence (5′→3′)** |
| --- | --- |
| **CesA1PCRFwd** | ATGGAAGCCTCTTCCGGCATGGTTGCTG |
| **CesA1PCRRev** | CTAGCAGTTGATACCACACTGACTATTG |
| **CesA3PCRFwd** | ATGATGGACTCAGAAGGAGAAGCTGGGG |
| **CesA3PCRRev** | CTAGCAGTTGATTCCACACATCTCAGAT |
| **CesA4PCRFwd** | ATGGCTGGCTTGATCACTGGCTCTAATT |
| **CesA4PCRRev** | TCAGCATTCTACACCACATTGTTTGAGA |
| **CesA6BPCRFwd** | ATGCATACCGGTGGTAGACTCATTGCTG |
| **CesA6BPCRRev** | TCATGTATCCTCACAATTCAATCCACAA |
| **CesA6CPCRFwd** | ATGGACACTAATGGAAGATTAGTTGCAG |
| **CesA6CPCRRev** | TCAATCACAATTCAACCCACAAAGTTCC |
| **CesA6FPCRFwd** | ATGGAAACCAATTTTGGGTTAGTTGCAG |
| **CesA6FPCRRev** | CTAATTACAATCCAATCCACATTCTTCT |
| **CesA7APCRFwd** | ATGGAAGCCAGCGCCGGACTAGTCGCTG |
| **CesA7APCRRev** | TTAACAGTTGATTCCGCATAGCTTGGTA |
| **CesA7BPCRFwd** | ATGATGGTTGTGGCTAGGCTCATCCTTC |
| **CesA7BPCRRev** | TCAACAACTAATTCCACATTGCTTAACA |
| **CesA8PCRFwd** | ATGATGCCATCTGGTGCTTCCCTCTGCA |
| **CesA8PCRRev** | TTAACAATCTATAGCAACACAAGTTTCA |

**Table S1**
