# Supplementary material for: Alfalfa Cellulose Synthase Gene Expression under Abiotic Stress: A Hitchhiker’s Guide to RT-qPCR Normalization
Source: PLoS One. 2014 Aug 1;9(8):e103808. doi: 10.1371/journal.pone.0103808 (PMC4118957; doi:10.1371/journal.pone.0103808)
Supplement: Table S2 — List of primers used for the RT-qPCR study. Name of the primers used for the RT-qPCR study, with the respective sequences. Details concerning the amplicons details (length, Tm), PCR efficiencies and regression coefficients are included. (DOC) [file pone.0103808.s008.doc]

| **Name** | **Sequence**  **(5′→3′)** |  | **Amplicon length (bp)** | **Amplicon Tm**  **(°C)** | **PCR efficiency (%)** | **Regression coefficient (R^2^)** |
| --- | --- | --- | --- | --- | --- | --- |
| **CesA1qPCRFwd** | AGGAAGGTTGGACAATGCAG |  | 147 | 81 | 87.2 | 0.999 |
| **CesA1qPCRRev** | CACGAGACACATAAACAAGCC |  |
| **CesA3qPCRFwd** | TCGATGGGCTTTAGGTTCAG |  | 143 | 79.4 | 100 | 0.996 |
| **CesA3qPCRRev** | TGAGAAGAGGAATGGAAGTG |  |
| **CesA4qPCRFwd** | TGTCCTCAATGCAACTCTCG |  | 112 | 79.2 | 91 | 0.997 |
| **CesA4qPCRRev** | TCTTGACCGGAAACTCATCTTC |  |
| **CesA6BqPCRFwd** | CCCTCTTCATATCCATCGCAG |  | 105 | 79.7 | 89.8 | 0.998 |
| **CesA6BqPCRRev** | CACCTCCAATCACCCAAAAC |  |
| **CesA6CqPCRFwd** | CAGGAATCACTACACCATCGG |  | 122 | 78.9 | 94.8 | 0.992 |
| **CesA6CqPCRRev** | CGGAGGGATAATCAGAGCATG |  |
| **CesA6FqPCRFwd** | TGCATATGCTTTGTGGCTTG |  | 100 | 77.4 | 105.6 | 0.984 |
| **CesA6FqPCRRev** | TTCCCTCATGACAGGAAACC |  |
| **CesA7AqPCRFwd** | GATGAAGCAAGACAACCACTG |  | 130 | 79.7 | 85.3 | 0.997 |
| **CesA7AqPCRRev** | CTGGGTTCATAAGTCTGTATCGG |  |
| **CesA7BqPCRFwd** | TTCTTGGTCACAATGGAGGAG |  | 144 | 80.4 | 88.8 | 0.997 |
| **CesA7BqPCRRev** | TGCTCAATACTGCCGATACAC |  |
| **CesA8qPCRFwd** | TCATACCAACGCTTACCAACG |  | 148 | 79.6 | 92.6 | 0.977 |
| **CesA8qPCRRev** | GAACCTCCAATCACCCAAAAC |  |
| **ActinFwd** | ACTGGAATGGTTAAGGCTGG |  | 147 | 82.7 | 103.3 | 0.998 |
| **ActinRev** | CCTCTTAGACTGTGCCTCATC |  |
| **TubulinFwd** | TTCGCCCGTGGTCATTATAC |  | 150 | 81.7 | 89.9 | 0.999 |
| **TubulinRev** | AAGAGAACCCAAACCAGATCC |  |
| **GAPDFwd** | CCCTCTCCCTGTACAAAACTC |  | 90 | 78.8 | 89.7 | 0.999 |
| **GAPDRev** | ACACGTAACACCAACCTTCC |  |
| **eIF5AFwd** | CGCGCAGAGAAACCATCAATC |  | 90 | 81 | 94.9 | 1 |
| **eIF5ARev** | TGAGGGTAGGTTTTGGATGCTC |  |
| **eIF4AFwd** | TGCTAAGTTGCCTGAAACCG |  | 127 | 81.2 | 89.7 | 0.999 |
| **eIF4ARev** | TGCCCATGTTTTCACCTTGC |  |
| **ADF1Fwd** | TCAAGGCGAAAAGGACACAC |  | 149 | 81.3 | 99.3 | 0.998 |
| **ADF1Rev** | AAAACAGCATAGCGGCACTC |  |
| **PAB4Fwd** | GCAAGTTTACGTGGGACCCTTC |  | 70 | 79.2 | 92.6 | 0.998 |
| **PAB4Rev** | GTTGAATTTTGCCCTGTCACCTG |  |
| **CyclophilinFwd** | CAAACTTTCCTGACGAGTCACC |  | 74 | 80.4 | 99.1 | 0.999 |
| **CyclophilinRev** | ACGGTCAGCAATTGCCATTG |  |
| **TFIIAFwd** | GGCATGTGACTCGAAATTGC |  | 145 | 80.4 | 92.2 | 0.998 |
| **TFIIARev** | ATGCTGGTTCCTGCAAGAAC |  |
| **UBC13Fwd** | ATTGGCTGCACATCATGGTG |  | 116 | 78.5 | 98.1 | 0.996 |
| **UBC13Rev** | TGGCCAACTTCCAAAAAGCC |  |
| **SuSyFwd** | TGCTGTCATCCTTTCTCCAC |  | 133 | 81 | 94.5 | 0.998 |
| **SuSyRev** | CTTGATACGTCTGAGCATCTCG |  |
| **PALFwd** | ATGAGGTGAAGCGTATGGTG |  | 141 | 84.4 | 93.5 | 0.997 |
| **PALRev** | CATCCCTAGCAGATTCAGACAG |  |
| **CADFwd** | TTCAAAGACTTTGGCCGAGG |  | 88 | 79.4 | 98.9 | 0.993 |
| **CADRev** | TGCCACCATTGTTGGGTTAAG |  |
| **CslD4Fwd** | AAGACCGCGCATCTTTCTGT |  | 93 | 80.2 | 93.5 | 0.990 |
| **CslD4Rev** | CACCCATCCTTAGTGCCCTG |  |
| **SK1Fwd** | CTCAGGCATCTAGGTCAGTTG |  | 76 | 79.8 | 88.4 | 0.993 |
| **SK1Rev** | GCCTCTGCTTCGTTTATTTCG |  |

**Table S2**
