# Supplementary material for: Alfalfa Cellulose Synthase Gene Expression under Abiotic Stress: A Hitchhiker’s Guide to RT-qPCR Normalization
Source: PLoS One. 2014 Aug 1;9(8):e103808. doi: 10.1371/journal.pone.0103808 (PMC4118957; doi:10.1371/journal.pone.0103808)
Supplement: Table S3 — CESAs from M. truncatula. Details concerning number of predicted transmembrane helices (TMHs, according to [47]) and the length of the putative CESAs from M. truncatula. (DOC) [file pone.0103808.s009.doc]

| ***M. truncatula* CESA** | **TMHs** | **Length (aa)** |
| --- | --- | --- |
| *MtCesA1* Medtr3g107520 | 6 | 1087 |
| *MtCesA3* Medtr3g030040 | 8 | 1078 |
| *MtCesA4* Medtr2g035780 | 8 | 1039 |
| *MtCesA6-B* Medtr8g092590 | 8 | 1098 |
| *MtCesA6-C* Medtr1g098550 | 6 | 1135 |
| *MtCESA6-F* Medtr3g007770 | 8 | 1048 |
| *MtCesA7-A* Medtr4g130510 | 6 | 1038 |
| *MtCesA7-B* Medtr8g063270 | 6 | 981 |
| *MtCesA8*  Medtr8g086600 | 6 | 991 |

**Table S3**
