# Supplementary material for: Alfalfa Cellulose Synthase Gene Expression under Abiotic Stress: A Hitchhiker’s Guide to RT-qPCR Normalization
Source: PLoS One. 2014 Aug 1;9(8):e103808. doi: 10.1371/journal.pone.0103808 (PMC4118957; doi:10.1371/journal.pone.0103808)
Supplement: Table S4 — Normalized Relative Expression for primary CesAs. Normalized Relative Expression values ± standard deviation and significance (Sig.) for the primary CesAs. Data were normalized using eif4A/TFIIA. (DOC) [file pone.0103808.s010.doc]

| **Time** | ***MsCesA1*** | ***Sig.*** | ***MsCesA3*** | ***Sig.*** | ***MsCesA6-B*** | ***Sig.*** | ***MsCesA6-C*** | ***Sig.*** | ***MsCesA6-F*** | ***Sig.*** |
| --- | --- | --- | --- | --- | --- | --- | --- | --- | --- | --- |
| 0h | 0.76±0.04 | a | 0.75±0.27 | a | 0.86±0.34 | abc | 1.00±0.23 | bcd | 0.93±0.12 | a |
| 24h | 0.74±0.12 | a | 0.81±0.24 | a | 0.66±0.13 | a | 1.40±0.39 | cd | 0.87±0.07 | a |
| 24h cold | 0.89±0.32 | a | 0.94±0.24 | ab | 0.96±0.27 | abc | 0.31±0.04 | a | 0.97±0.32 | a |
| 24h heat | 0.95±0.31 | a | 0.85±0.29 | ab | 0.90±0.28 | abc | 0.90±0.29 | bcd | 1.20±0.17 | a |
| 24h salt | 1.18±0.46 | a | 1.30±0.36 | ab | 1.15±0.31 | abc | 1.85±0.52 | d | 1.32±0.29 | a |
| 72h | 0.84±0.02 | a | 0.75±0.05 | a | 0.82±0.11 | abc | 1.46±0.36 | cd | 1.24±0.25 | a |
| 72h cold | 0.80±0.09 | a | 1.01±0.13 | ab | 0.95±0.14 | abc | 0.50±0.15 | ab | 0.63±0.22 | a |
| 72h heat | 1.55±0.59 | a | 1.18±0.39 | ab | 1.14±0.50 | abc | 0.95±0.39 | bcd | 1.09±0.32 | a |
| 72h salt | 1.35±0.51 | a | 1.37±0.29 | ab | 1.29±0.29 | abc | 1.77±0.22 | d | 1.37±0.66 | a |
| 96h | 0.77±0.04 | a | 0.74±0.07 | a | 0.92±0.15 | abc | 1.16±0.12 | cd | 1.06±0.14 | a |
| 96h cold | 0.78±0.02 | a | 0.95±0.14 | ab | 0.79±0.20 | ab | 0.68±0.20 | abc | 0.80±0.30 | a |
| 96h heat | 1.56±0.22 | a | 1.40±0.25 | ab | 1.72±0.11 | c | 1.30±0.42 | cd | 0.94±0.14 | a |
| 96h salt | 1.54±0.32 | a | 1.69±0.37 | b | 1.65±0.35 | bc | 1.77±0.31 | d | 1.14±0.17 | a |

**Table S4**
