# Supplementary material for: Alfalfa Cellulose Synthase Gene Expression under Abiotic Stress: A Hitchhiker’s Guide to RT-qPCR Normalization
Source: PLoS One. 2014 Aug 1;9(8):e103808. doi: 10.1371/journal.pone.0103808 (PMC4118957; doi:10.1371/journal.pone.0103808)
Supplement: Table S5 — Normalized Relative Expression for secondary CesAs. Normalized Relative Expression values ± standard deviation and significance (Sig.) for the secondary CesAs. Data were normalized using eif4A/TFIIA. (DOC) [file pone.0103808.s011.doc]

| **Time** | ***MsCesA4*** | ***Sig.*** | ***MsCesA7-B*** | ***Sig.*** | ***MsCesA7-A*** | ***Sig.*** | ***MsCesA8*** | ***Sig.*** |
| --- | --- | --- | --- | --- | --- | --- | --- | --- |
| 0h | 1.07±0.11 | ab | 0.93±0.18 | ab | 1.10±0.15 | bcd | 1.00±0.19 | abcd |
| 24h | 0.99±0.03 | ab | 0.84±0.12 | ab | 1.11±0.15 | bcd | 0.73±0.09 | abc |
| 24h cold | 0.88±0.34 | ab | 1.30±0.47 | ab | 0.78±0.29 | abc | 1.42±0.54 | bcd |
| 24h heat | 0.55±0.08 | a | 0.65±0.11 | a | 0.54±0.09 | a | 0.75±0.20 | abc |
| 24h salt | 1.54±0.69 | b | 1.30±0.59 | ab | 1.52±0.53 | d | 1.44±0.62 | cd |
| 72h | 1.41±0.04 | b | 1.33±0.12 | b | 1.43±0.02 | d | 1.65±0.25 | d |
| 72h cold | 0.75±0.07 | ab | 0.76±0.14 | ab | 0.66±0.13 | ab | 0.67±0.04 | ab |
| 72h heat | 0.74±0.12 | ab | 0.84±0.13 | ab | 0.87±0.11 | abcd | 0.61±0.13 | a |
| 72h salt | 1.37±0.35 | b | 1.20±0.38 | ab | 1.31±0.32 | cd | 1.47±0.55 | cd |
| 96h | 1.40±0.16 | b | 1.24±0.16 | ab | 1.46±0.13 | d | 1.26±0.03 | bcd |
| 96h cold | 0.73±0.12 | ab | 0.80±0.15 | ab | 0.62±0.10 | ab | 0.62±0.08 | a |
| 96h heat | 1.00±0.23 | ab | 1.17±0.31 | ab | 1.04±0.23 | bcd | 1.01±0.16 | abcd |
| 96h salt | 1.41±0.38 | b | 1.29±0.21 | ab | 1.52±0.29 | d | 1.46±0.37 | cd |

**Table S5**
