# Supplementary material for: Alfalfa Cellulose Synthase Gene Expression under Abiotic Stress: A Hitchhiker’s Guide to RT-qPCR Normalization
Source: PLoS One. 2014 Aug 1;9(8):e103808. doi: 10.1371/journal.pone.0103808 (PMC4118957; doi:10.1371/journal.pone.0103808)
Supplement: Table S6 — Normalized Relative Expression for for CAD, CslD4, PAL and SuSy. Normalized Relative Expression values ± standard deviation and significance (Sig.) for CAD, CslD4, PAL and SuSy. Data were normalized using eif4A/TFIIA. (DOC) [file pone.0103808.s012.doc]

| **Time** | ***CAD*** | ***Sig.*** | ***CslD4*** | ***Sig.*** | ***PAL*** | ***Sig.*** | ***SuSy*** | ***Sig.*** |
| --- | --- | --- | --- | --- | --- | --- | --- | --- |
| 0h | 0.81±0.08 | ab | 0.86±0.17 | ab | 0.78±0.15 | abc | 1.05±0.40 | abcde |
| 24h | 0.72±0.14 | ab | 0.69±0.05 | ab | 0.54±0.10 | a | 1.06±0.16 | bcde |
| 24h cold | 0.60±0.16 | ab | 0.95±0.58 | ab | 1.24±0.63 | abcd | 0.93±0.33 | abcde |
| 24h heat | 1.24±0.41 | ab | 1.07±0.53 | ab | 1.19±0.61 | abcd | 0.74±0.21 | abc |
| 24h salt | 1.24±0.10 | ab | 1.40±0.16 | ab | 0.78±0.18 | abc | 1.54±0.56 | cde |
| 72h | 1.00±0.30 | ab | 1.03±0.27 | ab | 1.10±0.27 | abcd | 1.22±0.18 | bcde |
| 72h cold | 0.73±0.25 | ab | 0.70±0.29 | ab | 0.67±0.08 | ab | 0.61±0.16 | ab |
| 72h heat | 1.50±0.69 | ab | 1.72±0.85 | ab | 1.51±0.53 | bcd | 0.78±0.16 | abcd |
| 72h salt | 1.24±0.09 | ab | 1.11±0.25 | ab | 0.82±0.07 | abc | 1.60±0.26 | de |
| 96h | 1.08±0.08 | ab | 1.23±0.21 | ab | 1.66±0.38 | cd | 1.70±0.35 | e |
| 96h cold | 0.90±0.37 | ab | 0.57±0.24 | a | 0.78±0.06 | abc | 0.51±0.12 | a |
| 96h heat | 1.28±0.25 | ab | 1.76±0.95 | b | 2.20±0.95 | d | 0.85±0.11 | abcde |
| 96h salt | 1.55±0.40 | b | 1.25±0.18 | ab | 1.13±0.08 | abcd | 1.58±0.23 | de |

**Table S6**
